# Modeling decision dynamics disentangles working memory, cognitive control and reinforcement learning and reveals clinical differences

**DOI:** 10.1371/journal.pcbi.1014796

**Published:** 2026-09-30

**Authors:** Krishn Bera, Alexander Fengler, Megan A. Boudewyn, Cameron S. Carter, Molly A. Erickson, James M. Gold, Steven J. Luck, J. Daniel Ragland, Andrew P. Yonelinas, Angus W. MacDonald III, Deanna M. Barch, Michael J. Frank

**Affiliations:** 1 Department of Cognitive & Psychological Sciences, Carney Institute for Brain Science, Brown University, Providence, Rhode Island, United States of America; 2 Department of Psychology, University of California, Santa Cruz, California, United States of America; 3 Department of Psychology, University of California, Irvine, California, United States of America; 4 Department of Psychiatry & Behavioral Neuroscience, University of Chicago, Chicago, Illinois, United States of America; 5 Department of Psychiatry, University of Maryland School of Medicine, Baltimore, Maryland, United States of America; 6 Department of Psychology, University of California, Davis, California, United States of America; 7 Department of Psychology, University of Minnesota, Minneapolis, Minnesota, United States of America; 8 Department of Psychology, Washington University in St. Louis, St. Louis, Missouri, United States of America; Brain and Spine Institute (ICM), FRANCE

## Abstract

Various neurocognitive processes contribute to reinforcement learning (RL) and decision making, requiring careful task designs and computational modeling to disentangle them. People rely on capacity-limited working memory (WM) to rapidly and flexibly adapt behavior during learning, together with slower incremental RL processes that support robust long-term retention. The RLWM paradigm was developed to isolate these contributions by manipulating WM demands during learning. Empirical and computational advances support the separability of such processes, but only imperfectly, because a given choice can often equally be attributed to use of WM vs. RL. Here, we show that jointly modeling decision dynamics – accounting not only for choices but also response time distributions, together with hierarchical Bayesian parameter estimation – improves the ability to disentangle separable learning and cognitive control processes. Despite added complexity, models with decision dynamics improved parameter recovery and yielded accurate out-of-sample prediction for choices in a held-out test phase after learning. In contrast, models fit only to choices produced an inflation of RL learning rates and failed the out-of-sample test. Moreover, joint modeling also revealed a novel neurocognitive process by which participants proactively widen decision boundaries to increase response caution under increased WM load. Applying our model to patients with schizophrenia revealed a deficit in proactive control mechanisms as well as slowed incremental RL, which were masked by previous choice-only models, while replicating previously established WM deficits. In summary, we show how joint modeling enables accurate, model-based and mechanism-oriented computational phenotyping of psychopathologies.

## Author summary

Humans learn from feedback using multiple brain systems in parallel: reinforcement learning, which gradually strengthens successful behaviors through trial and error, and working memory, which supports rapid learning by holding recent information in mind. These systems often drive the same outward behavior, making their contributions notoriously difficult to disentangle. Here, we show that analyzing not only what people choose but how quickly they respond fundamentally changes what we can learn about these processes. We introduce a computational framework that captures both learning and decision dynamics in a single model, and discover that people proactively slow down their responses when they anticipate greater memory demands, a cognitive control strategy not previously identified in this context. As an independent check, we used the model to predict a later memory-free phase it had never been fit to; it succeeded, while choice-only predictions diverged from people’s behavior. This model also reveals that individuals with schizophrenia show both a blunting of gradual learning and a failure of this proactive adjustment, deficits that were entirely hidden from prior analyses based on choices alone. Our approach is broadly extensible, allowing researchers to formulate and compare theories that jointly characterize learning processes and decision dynamics within a unified model.

## Introduction

Humans excel at orchestrating goal-oriented behaviors that depend on learning from past experiences. This learning process is widely understood through the computational framework of reinforcement learning (RL) [1]. The RL framework has contributed immensely toward understanding the computational principles of maximizing expected long-term rewards [2–4] as well as the neural basis of how cortico-striatal circuits learn values using dopaminergic reward prediction errors [5–9]. It is moreover well established that the RL-related computations in the brain are augmented by parallel contributions of multiple neurocognitive systems. Executive functions such as working memory (WM), cognitive control and attention facilitate RL computations by simplifying the state or action space, allowing preferential weighting of features, manipulation of representation abstractions and leveraging predictions/expectations from other non-RL processes [2,10–13]. The incorporation of such component processes in computational frameworks has been essential in explaining human-like learning behaviors.

A central difficulty is that behavior in most tasks described as “reinforcement learning” reflects at least two dissociable processes, and does not, by itself, indicate which one produced a given adaptation. Trial-to-trial improvements can arise from slow, incremental updating driven by reward prediction errors, canonically supported by cortico-striatal plasticity, or from a fast, flexible WM system, supported by prefrontal cortex, that maintains recent stimulus-response-outcome associations [12,14,15]. Because the fast WM component dominates trial-by-trial adaptations, it can mask the slower reinforcement learning signal and confound its estimation. Earlier work showed exactly this: flexible trial-by-trial adjustments during learning are carried largely by WM, whereas the slower RL contribution is more clearly revealed by a separate test phase administered to assess robustness of learned associations when trial-by-trial adjustments are no longer required [15]. This observation motivated the Reinforcement Learning - Working Memory (RLWM) paradigm, which manipulates WM load parametrically during learning, so that the two processes make separable predictions and can be estimated jointly rather than confounded [12,16–18]. Using this approach, slowed learning has been attributed to limitations in WM rather than to incremental RL: in patients with schizophrenia [19], in carriers of genetic variants affecting prefrontal catecholamine function [12], and in healthy aging [20].

This dissociation, however, is not guaranteed by the design alone: RL and WM contributions can remain conflated. Because both systems frequently converge on the same optimal action, their parameters remain partially confusable [21,22]. This residual confound carries two consequences that motivate the present work. First, fitting models to choices alone can systematically bias the recovered RL parameters, so that a genuine clinical difference in the incremental learning rate may go undetected even when the paradigm is otherwise adequate to expose it; overcoming this requires sources of constraint on the learning process beyond the choices themselves. Second, a model that claims to have separated the two processes still requires an independent criterion to confirm that the separation is genuine. One variant of the RLWM paradigm includes a post-learning test phase [23], inspired by earlier work mentioned above [15]. This test phase supplies such a criterion, because it indexes participants’ retention of learned stimulus-reward associations when WM is no longer available (because the test occurs after delay and many blocks of intervening trials with new stimuli). Thus a model that correctly isolates incremental RL processes during learning should predict test phase choices (out-of-sample) without systematic bias. Prior RLWM work has not used the test phase in this way, either omitting out-of-sample validation of the dissociation or obtaining it only by fitting additional free parameters to the test data. We address the first of these two consequences with response time modeling, and the second with an out-of-sample analysis of the held-out test phase.

One source of additional constraint on the accurate characterization of the parallel learning processes, and the one we exploit here, is response time (RT). The vast majority of RL models are fit to choices alone, ignoring the rich information contained in the joint distribution of choice and response times. While choice-only models provide a parsimonious explanation of the rate of learning across trials and in different conditions (e.g., WM loads), they are incomplete accounts of the underlying true generative process. Conversely, RT distributions reflect the shorter-scale dynamics of the underlying decision process and are influenced by the parallel learning contributions from multiple neurocognitive processes. Moreover, joint modeling of choice and RT distributions can in principle better dissociate these contributions from underlying learning processes, because RTs impose additional informative constraints on the parameter estimates. For example, even when predicted choices align between parallel learning components, RL predicts more incremental speed-ups in RT over trials compared to WM. In addition, the decision dynamics and response times during learning are also affected by various factors such as the number of choice options [24], value-based differences [25,26], conflict [27], and policy compression [28], none of which can be captured with a reliance on choice distributions alone.

Although there has been recent theoretical interest and application in jointly modeling learning and decision processes in simple RL scenarios [25,26,29–36], a broader investigation of how doing so might lend insights into component systems governing learning (as in the RLWM paradigm) is traditionally hampered by prohibitively expensive computational procedures in model fitting. The computational cost may arise from a lack of a closed-form likelihood function for the decision process, a more complex learning process account, or a combination of both [37–40]. This limitation has two critical consequences: it restricts the ability to test a larger set of competing hypotheses about learning, and it obscures the specific cognitive processes that generate the behavior.

More specifically, this methodological constraint creates a significant roadblock in understanding within-trial decision dynamics and across-trial adaptations governed by both incremental RL and capacity-limited WM. While one study found that the RLWM model can be combined with a linear ballistic accumulator (LBA) [41] to jointly model choices and RTs [18], this approach has not been adopted widely and did not explore alternative decision process dynamics beyond the standard LBA, which might be important for disentangling component cognitive processes. Moreover, their approach did not apply Bayesian parameter estimation, which allows one to appropriately assign uncertainty in inferring the relative contributions of learning-related RLWM and decision process parameters. One reason for this lacuna is that robustly estimating a combination of learning and decision processes can be prohibitively costly due to computationally expensive evaluations of the learning process and intractable decision process likelihoods [37–39]. Accordingly, the computational modeling in [18] was limited to a standard LBA as the decision process and utilized single point estimation methods for parameter fitting, precluding computation of their uncertainty. Moreover, the authors did not explore how cognitive demand, operationalized as WM load, modulates decision dynamics, nor whether taking these dynamics into account can further disentangle learning processes in various populations.

To address these parallel methodological and theoretical challenges, we develop a new computational approach and apply it to behavioral data from 255 participants spanning four diagnostic groups. Our primary analyses contrast healthy controls (HC, *n* = 87) with patients with schizophrenia (SCZ, *n* = 67); results for bipolar disorder and major depression are reported in the supplement. This work makes three distinct contributions. First, methodologically, we introduce an approach that enables efficient Bayesian inference of model parameters, including their uncertainty, by using learned surrogate likelihood functions (via LANs) for the decision process [37,38] in conjunction with end-to-end differentiable learning process likelihoods. Likelihood Approximation Networks (LANs) are neural networks trained on simulations to approximate the likelihood of decision models that lack a closed-form expression, and are part of the broader family of approaches in simulation-based inference (SBI). Together, this approach enables the use of fast, gradient-based inference methods such as Hamiltonian Monte Carlo or Variational Inference (VI) for a broad class of previously intractable models for arbitrary combinations of learning and decision process models. Our computational approach makes fitting complex joint models computationally feasible using hierarchical Bayesian parameter estimation. Second, theoretically, we apply this framework to the RLWM paradigm to provide the first generative account of how individuals adapt to higher WM load by engaging in proactive cognitive control, specifically by widening their decision boundary to trade speed for accuracy. Notably, we also find that, compared to the choice-only models that dominate this literature, incorporating RTs into model fitting improves parameter estimation and reduces bias of the underlying RL learning rate parameter in spite of the increased model complexity. We confirm this improvement out of sample: RL learning rates estimated from the learning (train) phase alone predict choices in the held-out test phase, a WM-free criterion never used for fitting, whereas choice-only estimates systematically overpredict test phase accuracy. Finally, clinically, we leverage this improved and independently validated estimation to reveal previously undescribed alterations in individuals with schizophrenia: a reduced RL learning rate, masked in prior choice-only analyses of this population, together with a failure of proactive control (decision bound adjustment). We discuss the implications of these findings and their connection to the broader clinical literature in the Discussion.

## Results

Participants performed the RLWM task [12,17,19,42] which requires blockwise learning of a set of 2–5 unique stimulus-response pairs. Within each block, every stimulus was presented 10 times, and on each presentation, participants selected one of three responses (Fig 1A), with reinforcement feedback (correct or incorrect) provided thereafter. The number of unique stimulus-response pairs to be learned within a block is termed set size. Working memory load was varied between blocks by the set size and within blocks by the number of intervening trials (delay) between successive presentations of the same stimulus. This design dissociates the two learning systems because working memory, but not incremental RL, is sensitive to set size and delay: RL strengthens values gradually with reward history regardless of load, whereas WM contributions weaken as more items must be held and as more trials intervene. Each participant performed 10 blocks of this task.

**Fig 1:**
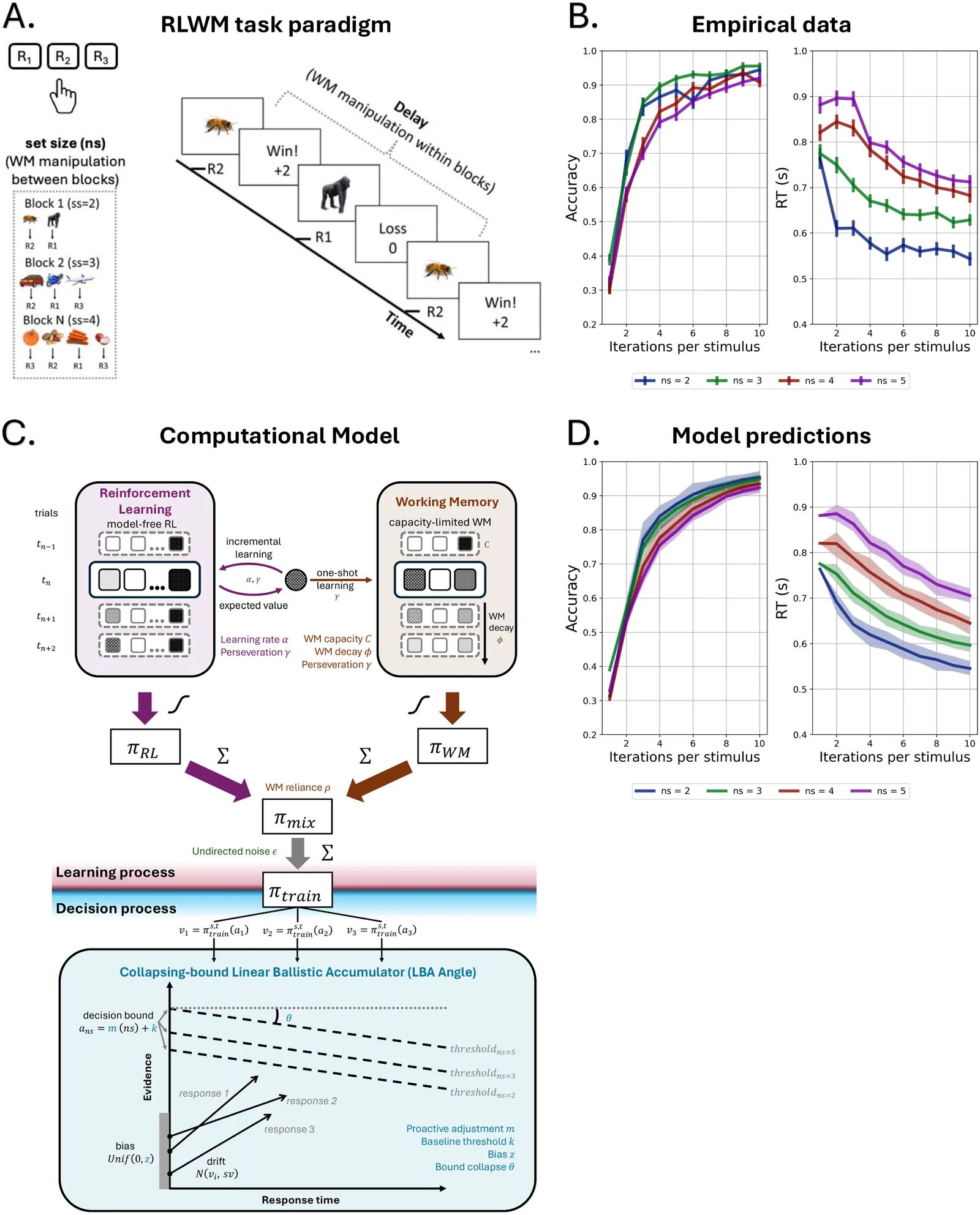
RLWM task, behavior and model. **(A)** RLWM task design. Participants perform an instrumental learning task where they learn a fixed number of stimulus-response pairs per block (set size). Each stimulus is shown 10 times within a block. On every trial, participants choose between a set of three responses. The paradigm contains a number of blocks, with set sizes ranging from 2 to 5. The WM load in the paradigm is manipulated parametrically with between-block set size manipulation and within-block delay (the number of elapsed trials since the last exposure to a given stimulus). Three example blocks are shown alongside a trial sequence. **(B)** Typical behavioral phenomena in the RLWM paradigm. Learning is plotted as a function of iterations per stimulus in terms of accuracy and RT. Higher set sizes exhibit less accurate and slower performance as compared to lower set sizes. Error bars show SEM. **(C)** Schematic overview of the PADDL (joint) model – the learning component is combined with the decision process. The learning component has parallel contributions from two modules – reinforcement learning and working memory. The RL module learns stimulus-response associations via slow, incremental learning from reward prediction errors. RL has persistent memory with no capacity constraints. The WM module allows fast learning via one-shot updating but is capacity-limited and prone to forgetting/interference. The RL and WM policies are derived independently from the respective Q-values, and the policies are combined via a weighted mixture to derive the final trial policy that also incorporates undirected noise. The decision process is a linear ballistic accumulator with collapsing bounds (LBA Angle) parameterized by bias (starting point variability), rate of bound collapse, and a decision bound that is a linear function of set size. The final trial policy informs the drifts of each response accumulator. **(D)** Model predictions using posterior predictive checks (for HC group). Learning curves for each set size plotted in terms of accuracy and RT for the model simulations. The model simulations were generated by sampling the posterior and using the post hoc absolute fit method. Shaded regions show 94% HDI ranges of the group mean. (Fig 1A is adapted from [44], © 2025 The Authors, under CC BY 4.0).

We first examined the learning curves across participants in both groups and as a function of set size. The main behavioral findings replicate those in several previous studies [12,16,17,19,20]. First, accuracy increased monotonically as a function of the number of stimulus iterations (Fig 1B). Such incremental learning, a function of the number of previous correct choices, is a basic marker of the RL process [12,17,42,43]. Second, accuracy and response times both improved more quickly over trials in lower set sizes than higher set sizes. Performance moreover degraded with increasing delay between subsequent stimulus encounters, especially in higher set sizes (Fig 1B). These latter patterns are evidence of WM contributions to learning which canonically degrade with set size and delay. While all groups showed learning in terms of increased accuracy and faster response times with more stimulus iterations, the HC group reached asymptotic performance faster than the schizophrenia group (Figs 1B and 5A). We refer readers to [44] for a detailed statistical treatment of these behavioral findings in the same dataset. Here we focus on the novel computational model analysis of RLWM decision dynamics, assessing the insights gained by jointly modeling RTs and choices.

For computational modeling of the data, we began with a recent version of the RLWM computational model [42] which showed good fits to choice behavior and adequate parameter recovery. We then augmented this model to capture the decision dynamics using the linear ballistic accumulator (LBA), suitable for modeling choices and RTs among multiple response options [41]. In this model, multiple accumulators representing each response option race toward a common decision boundary, with drift rates determining their slopes. The drift rates of each accumulator were adjusted as a function of the RL and WM modules, such that those response options with the greatest reward history or recent updates in WM would have the highest drift rate, leading to faster and more accurate choices. RT distributions emerge from this model due to both learning processes and variability across trials in drift and starting point. We considered multiple variants of this model, including those with decision boundaries that could be adjusted as a function of task demands across blocks as well as adjustments to decision bounds that could occur within a trial (the LBA Angle variant, in which the boundary collapses over the course of a trial; see below). We fit all models with hierarchical Bayesian parameter estimation, offering a principled approach to quantify the full posterior distribution of plausible parameter values and capitalizing on the complete dataset by pooling information from the group to inform estimation of individual subject parameters. All modeling details are presented in section Computational Modeling; here we give a high-level overview.

As in prior RLWM studies, the learning module modeled parallel contributions from RL and WM to the behavioral policy using simple prediction error based learning. The WM system enabled one-shot updating of action values (with learning rate set to 1) and featured an additional forgetting/interference mechanism to account for WM decay from intervening trials. The final behavioral policy was computed as a weighted mixture of RL and WM policies, where the mixture weight accounted for WM capacity limitations (i.e., WM was weighted to a greater extent when the task demands were achievable within one’s estimated capacity).

The joint model, to account for choice and RT distributions, replaces the conventionally used softmax action selection decision policy with the LBA (with potential for additional modulations of decision boundaries). The drift rates of each accumulator were adjusted as a function of the RL and WM modules, leading to changes in choice and RT distributions as noted above. We further tested the hypothesis that people use proactive control by adjusting decision boundaries as a function of anticipated WM load (in higher set sizes), allowing them to mitigate a speed-accuracy trade-off. We refer to our proposed model as Proactive Adaptation of Decision Dynamics in Learning (PADDL). We compare our proposed PADDL model with the choice-only RLWM model that employs a softmax action selection decision policy, typically used to disentangle RL and WM contributions in this paradigm.

We first confirmed in the HC group that model fits reproduced several past findings before turning to the novel results enabled by the PADDL (or joint) model. First, whereas the WM system rapidly updates contingencies (effective learning rate of 1), the RL learning rate was recovered to a much lower value $\mu_{\alpha }^{HC}=0.0073$, reflecting the incremental contribution of RL. (Although these learning rates are numerically small, they exert an appreciable influence on behavior. The learned Q-values show small differences but they are strongly magnified in the policy (due to high inverse temperature softmax parameter), so incremental RL produces value separation that is expressed in both choices and RTs, most visibly at higher set sizes and later trials where the WM contribution is largely exhausted. Ablation analyses confirm this: inflating α predicts RTs that decline faster than observed (Fig 3A, 3B and 3D), and setting α=0 fails to reproduce the empirical accuracy curves and predicts systematically slower RTs (Fig B in S1 Text)). Conversely, while WM exhibits fast learning, it is subject to interference/forgetting from previous trials (the posterior mean for the WM decay rate was recovered as $\mu_{\phi }^{HC}$ = 0.0964) and has limited capacity (the fitted WM capacity $\mu_{C}^{HC}$ = 2.6940 is smaller than the highest set size 5), such that the RL process contributes to a greater extent as set size exceeds capacity. We additionally estimated a separate learning rate for negative prediction errors, capturing the well-documented tendency for learning to be attenuated after negative outcomes and thus for agents to persist in previously chosen actions despite disconfirming feedback, sometimes termed perseveration. The recovered estimates for the perseveration ($\mu_{\gamma }^{HC}$ = 0.0966) also suggested that negative feedback was neglected relative to positive outcomes, and a substantial amount of behavior was attributed to undirected noise or attentional lapses ($\mu_{\epsilon }^{HC}$ = 0.2724) [19,42].

Next, we describe the key findings across participant groups afforded by the joint (PADDL) model. The model accurately characterizes the decision dynamics at both across-trial and within-trial timescales. We then show that the model accurately captures out-of-sample test phase choice distributions. Subsequently, we leverage this theoretical advance to summarize performance differences via interpretable model parameter distributions to understand clinical contrasts between different groups.

**Box 1. Glossary of model parameters**

**Table:** 

Model parameters		
Symbol	Module	Parameter details
α	Learning (RL) 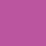	RL learning rate; governs incremental value updating (Eq. 1).
γ	Learning (RL & WM) 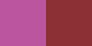	Perseveration; weighting of negative feedback to capture ‘stickiness’ in responses; applied in both RL and WM updates (Eqs. 1–2).
ϕ	Learning (WM) 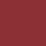	WM decay; reversion of WM values toward baseline across intervening trials (Eq. 3).
*C*	Learning (WM) 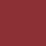	WM capacity (number of active memory slots) (Eq. 5).
ρ	Learning (WM) 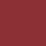	WM reliance; baseline propensity to use WM over RL (Eq. 5).
ϵ	Learning 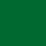	Undirected noise / lapse rate in the behavioral policy (Eq. 7).
*k*	Decision process (LBA Angle) 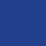	Baseline threshold (intercept) (Eq. 8).
*m*	Decision process (LBA Angle) 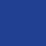	Proactive adjustment (threshold slope on set size) (Eq. 8).
*z*	Decision process (LBA Angle) 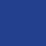	Bias (start-point variability of accumulation).
θ	Decision process (LBA Angle) 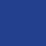	Boundary collapse angle.

### General findings across groups

#### PADDL model captures both choice and RT behavior in the RLWM paradigm.

We first evaluated the ability of the PADDL model to capture behavior of the participants in the RLWM task. Across both groups, posterior predictive checks revealed that the model shows good alignment with empirical data for both choices and RTs (Figs 1D and 5A). Accuracy increases, and RT decreases, as learning progresses (in terms of the encountered stimulus iterations), and both effects are attenuated with higher set sizes as the WM load increases. The model was particularly effective at capturing the set size-related RT variability during the initial stimulus iterations.

#### Joint modeling of choice and RT more accurately disentangles RL from WM components.

We next evaluated whether including RTs in the PADDL model imposes informative constraints to improve parameter estimation even for parameters of the model that are typically used to explain choice distributions alone. We hypothesized that fitting a choice-only model to the data generated from a joint choice-RT generative process could introduce systematic biases in parameter estimation. We evaluate this hypothesis by fitting the choice-only (softmax) as well as the PADDL model to empirical and synthetic datasets.

We first compared parameter estimates of the choice-only (softmax) and the PADDL model (with the evidence accumulation decision process), when fitted to the empirical data. Fig 2A shows that the softmax (choice-only) model overestimates RL learning rate, WM reliance and WM capacity parameters relative to the PADDL model. Although one cannot yet determine from this exercise alone which parameters are more accurate, we reasoned that maximization of softmax choice likelihood alone provides limited information to disentangle RL vs. WM processes. Conversely, RTs induce a powerful constraint on the joint probability p(choice=c,RT|parameters), fundamentally changing the estimation landscape. By requiring the model to simultaneously account for both what was chosen and how quickly it was chosen, the model is forced to estimate parameter values that are consistent with both aspects of the behavioral data. To validate this account, we generated synthetic choice and RT data using the PADDL model, and then fit that data with both the PADDL model and the choice-only model. Fig 2B shows a similar tendency of the softmax model to overestimate parameters such as RL learning rate, WM reliance, and WM capacity. In addition, the noise and perseveration parameters were consistently underestimated in the softmax model. This exercise confirms that when ground truth parameters from the PADDL model are known, the softmax model is less accurate at recovering them.

**Fig 2:**
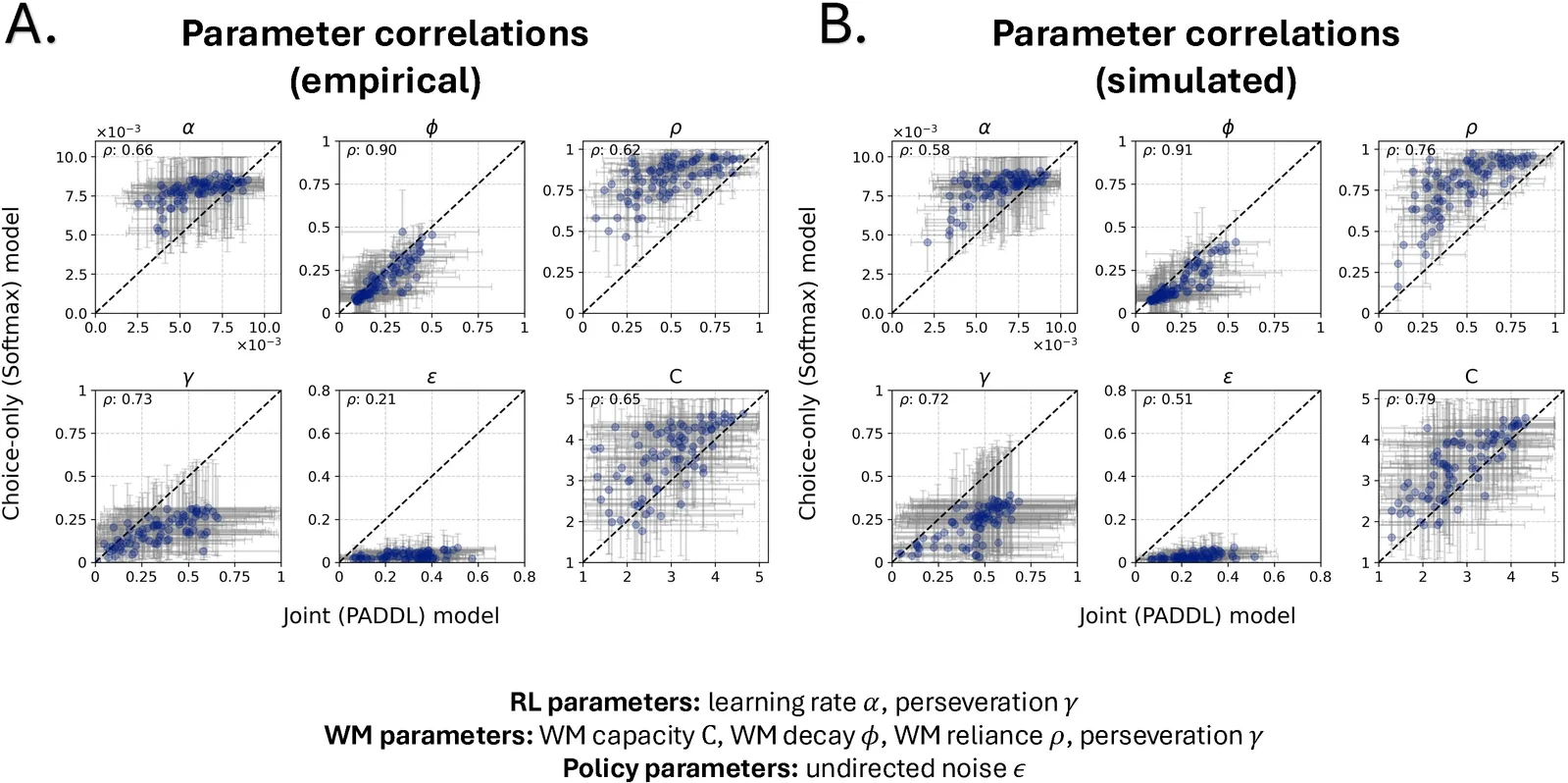
Comparison of the recovered parameters from the choice-only model and the joint PADDL model. The plots denote correlations between the recovered parameters on (A) empirical dataset and (B) simulated (synthetic) dataset. The simulated dataset was generated using the PADDL model. Error bars denote 94% HDI ranges for both axes.

How do RTs help inform parameter estimates? Note that although the RL module is more incremental than the WM module, the two will often agree as to which action has highest value in terms of the rank ordering of actions. In contrast with the choice distributions, RTs provide more continuous outputs, and in particular, RTs should decline more incrementally over time when the RL process is dominant (i.e., when capacity is exceeded). RTs are also informative about proactive control strategies, whereby participants may be more cautious with increasing WM demands, even before learning accumulates. To systematically explore the contributions of each mechanism to RTs, we performed two ablation experiments (Fig 3). Each removes or replaces a single mechanism while holding every other learning and decision parameter fixed, so that any resulting misfit to the RT data isolates the contribution of that one mechanism. The first ablation, presented here, targets the RL learning rate; the second, presented in the proactive control section below, targets the set size dependent decision threshold.

**Fig 3:**
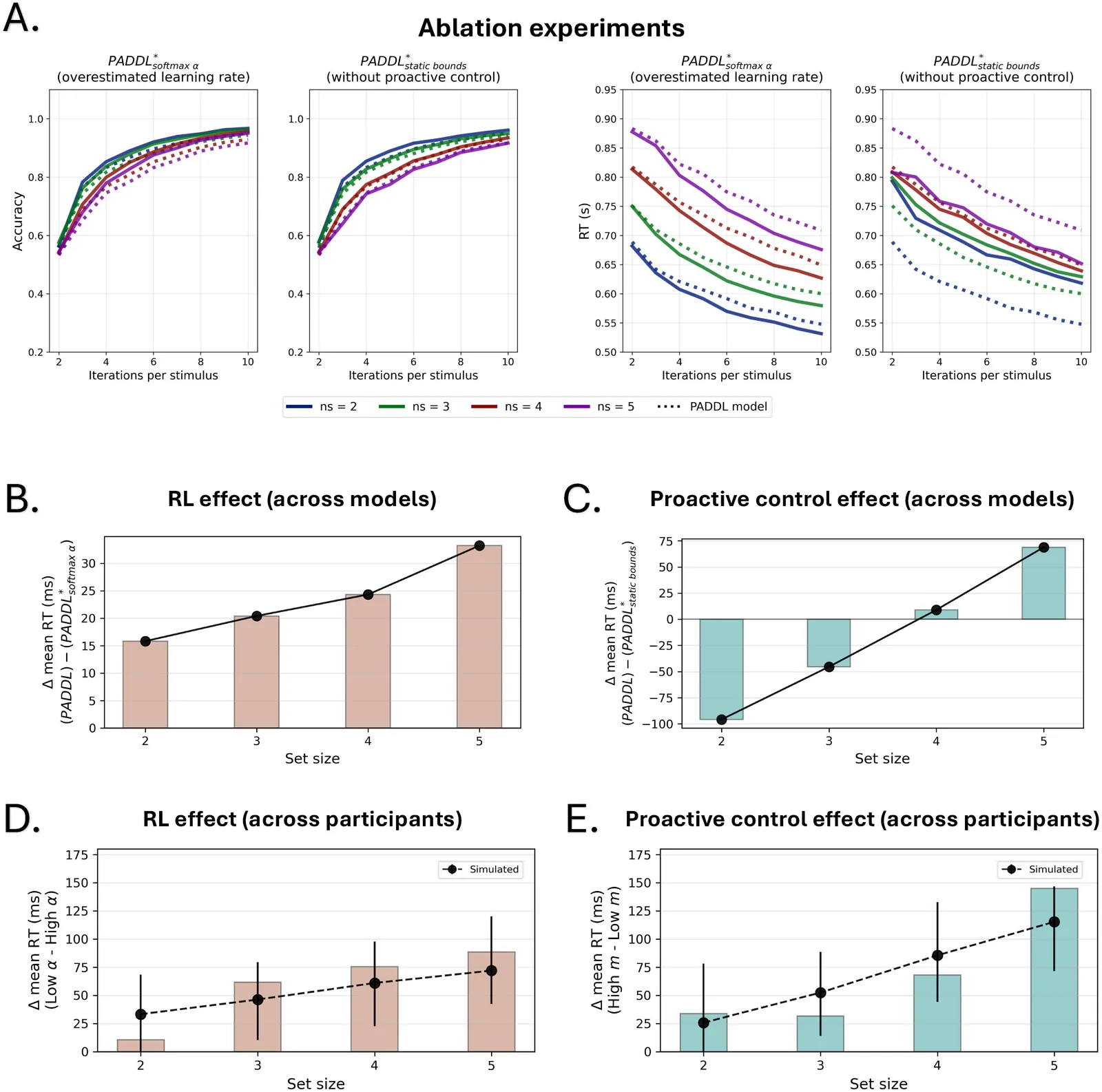
Each ablation removes or replaces a single mechanism of the joint model while holding all others fixed, isolating that mechanism’s contribution. **(A-C)** Ablation experiments show that PADDL provides better fits and identifies key diagnostic RT patterns. **(A)** Accuracy (*left*) and RT trends (*right*) for the $PADDL_{softmax\;\alpha }^{*}$ model which uses the estimated learning rate α from the choice-only softmax model and the $PADDL_{static\;bounds}^{*}$ model (model with static decision bounds, i.e., without proactive control). The PADDL trends are plotted in dashed lines for comparison and those from the ablation/alternate models are plotted in solid lines. Static bounds fail to capture the large differences in RTs by set size, whereas softmax α produces progressively faster RTs over learning particularly for higher set sizes. **(B)** Summary statistic showing RT discrepancy by set size in late trials for PADDL compared to the softmax-recovered, overestimated learning rate. **(C)** Summary statistic showing RT discrepancy by set size in early trials for PADDL compared to the static bounds model. **(D-E)** Posterior predictive checks show that PADDL captures empirical RT patterns diagnostic of RL learning rate and proactive decision threshold adjustment, as identified in panels B and **C. (D)** RT differences in empirical and simulated data between low- and high-α participant subgroups (in HC) during late/asymptotic learning phase. **(E)** RT differences in empirical and simulated data between high- and low-*m* participant subgroups (in HC) during early learning phase. The difference in RT is calculated between the median-split, high and low parameter participant groups as determined from the PADDL model fits. The error bars on simulated trends denote 94% HDI ranges.

The first ablation comparison is diagnostic of how the RL learning rate contributes to RTs. We focus on later trials and higher set sizes because that is where RL, and hence sensitivity to α, dominates. In particular, later trials allow RL sufficient experience to accumulate, and as set size increases, the WM contribution is weakened due to capacity limitations [12]; hence RTs in that regime are the most informative read-out of the learning rate. An overestimated α implies learned values that separate too quickly, which manifests as RTs that decline too fast over learning; a correctly estimated α does not. To test this notion, we simulated RTs from the joint PADDL model twice: once using the αs estimated by the PADDL model, and once using those from the softmax model. Posterior predictive checks in Fig 3A and 3B confirmed this interpretation about differential RT predictions from the RL and WM modules. We observed that when learning rates are estimated from the choice-only model, the simulated RTs decline faster than observed empirically, and moreover that this difference became increasingly apparent in the higher set sizes and in later trials. This is because RL is relatively more dominant under high WM load, and the (right) tail of the RT distribution constrains the RL parameter estimates. As predicted, the joint PADDL model more appropriately captures the RT dynamics, specifically at higher set sizes when RL is more dominant. This analysis revealed a novel signature of variations within RL that are manifest in terms of progressive changes in RT over learning in higher set sizes.

Posterior predictive checks further confirmed that this signature is apparent in those participants with high vs. low estimated PADDL learning rates: those with lower learning rates showed progressively slower RTs in high set sizes during the asymptotic learning phase, and these discrepancies were well captured by the PADDL model (Fig 3D). Finally, while this ablation experiment shows the consequences of omitting the decision dynamics during learning, leading to RL overestimation, we also confirmed that the small RL learning rates estimated from PADDL still contribute appreciably to observable behavior. To do so, we ran PADDL simulations setting RL α=0, effectively removing the RL process altogether; these simulations confirmed that the choices and RTs failed to match empirical data without RL contributions (Fig B in S1 Text).

#### PADDL model improves parameter recovery despite added complexity.

These conclusions about RL learning rate overestimation in choice-only models depended on the assumption that choices and RTs are generated by a common process (i.e., the synthetic data were generated from the LBA), and it is in theory possible that the altered estimates of RL learning rates resulted from model misspecification. We next turned to a stronger test: we compared parameter recovery for the choice-only model when it was used to generate the data (using a softmax decision policy) against recovery from the PADDL model. An improvement in recovery would be striking here because the joint model is more complex, having all the same parameters plus the decision process parameters. But as noted above, because RL and WM systems both converge on the same optimal discrete policy, they can partially mimic each other [22] and therefore hinder recovery when using choice-only models.

We simulated a synthetic dataset based on the parameters for each model that fit best to our empirical dataset, and attempted to recover the true data-generating parameters. We simulated the dataset using the mean of all participant-level parameter posterior distributions. Fig F (panel A) in S1 Text shows that the PADDL model performed well and was able to recover all parameters with reasonable accuracy. Notably, even for the parameters common between models, it recovered all parameters as well as or better than the choice-only model (Fig F (panel B) in S1 Text). The learning rate α was significantly better recovered with the PADDL model ($Z_{\alpha }=2.709,p_{\alpha }=0.0067$). These improvements in parameter recovery are notable given that they are applied to parameter ranges that best explain the empirical data across both clinical and non-clinical populations. (We note here that parameter recovery quality is expected to vary across parameters, reflecting differences in identifiability, interdependence, and noise levels. Non-identifiability can be localized to specific parameter subspaces, and critically, recovery is often less robust in parameter regions associated with poorer overall task performance, a characteristic typical of clinical datasets. Therefore, beyond the partial parameter trade-offs inherent in the model structure, the recovery statistics must be evaluated relative to the parameter distributions observed in the empirical (and clinical) data and cannot be numerically compared to those from studies of younger or healthier populations, where identifiability (even for choice-only models) may be higher.) An additional point to note here is that, in spite of the fewer free parameters, the choice-only softmax model was flexible enough to account for the group-level accuracy learning curves across set sizes (Fig A in S1 Text).

Taken together with the previous section, these results suggest that the PADDL model is a better generative model for the empirical data and reinforce the notion that modeling of choice and RT data can improve parameter identification in cognitive models [45]. The latter point is specifically important because the RTs can provide more refined continuous data that inform the RL learning rate over trials.

#### Proactive control over decision boundaries under varying WM load.

Beyond improving parameter recovery, modeling RTs also allows us to assess other neurocognitive processes not typically studied in RL (including the RLWM paradigm), such as speed-accuracy trade-offs. In sequential sampling models (including the linear ballistic accumulator used here), speed-accuracy trade-offs are well captured by adjustment of the decision boundary (sometimes referred to as the decision threshold). With a higher threshold/boundary, more evidence is accumulated before committing to a choice, overcoming the impact of accumulator variability and thus improving accuracy at the expense of speed [41,46]. Moreover, it is a well-established finding that when faced with tasks that require cognitive control to maintain high accuracy, participants adjust their decision boundaries as a way to impose response caution, and that this relates to mechanisms within prefrontal cortex and basal ganglia [27,47,48]. In the RLWM paradigm, we hypothesized that participants might proactively adjust the decision boundaries as a function of WM load. At the start of each block, participants were shown all the stimuli they would encounter in that block. This information about set size could potentially allow participants to proactively increase the decision thresholds – imposing more response caution when they know choices might be harder to remember.

To test this idea, the PADDL model allowed the decision boundary *a* to vary linearly with set size such that a=m·ns+k, with *k* reflecting the baseline threshold and *m* the set size dependent adjustment or slope. Note that the base model without set size-dependent decision bounds is a strictly nested model under the PADDL model because setting *m* to 0 recovers the base model with static decision bounds. This nesting is what makes the static bounds model the correct comparison for the proactive control claim: it shares every learning and decision parameter with PADDL and differs only in whether the threshold scales with set size, so a difference in fit cannot be attributed to anything else. The diagnostic signature is the difference in RTs across set sizes during the very first trials of each block, before any feedback in that block could have produced differential learning. Because set size is revealed to participants at block onset before any learning, a set size difference in RT at that point cannot reflect differences in learning or WM and must instead reflect a threshold set in anticipation of load. A static bound cannot produce this pattern, whereas the proactive mechanism can. Across all participants (Fig E in S1 Text and Table A in S1 Text), we found that the 94% highest density interval (HDI) of the threshold slope parameter *m* was recovered as a positive value and did not contain zero. Moreover, posterior predictive checks revealed that this adaptive threshold mechanism was necessary to capture the large differences in RTs across set sizes even from the very early learning phase, and that the corresponding static bounds model failed to do so (Fig 3A and 3C). Summary statistics showing set size-induced RT effects (as a result of proactive decision bound modulation) confirmed that proactive adaptation parameter *m* accurately characterizes the observed differences in RTs across set sizes (Fig 3E). We also confirmed that this proactive control model outperformed alternative models in which RTs were slowed due to policy uncertainty. (The $\pi_{H}$ model proposed in [18] did not improve the quality of fits over the PADDL model as assessed by posterior predictive checks. Moreover, when adding the proactive control mechanism within the $\pi_{H}$ model we also observed similar proactive adjustment of decision bounds; hence this effect is additional to any effects of policy uncertainty.) This suggests that the proactive control mechanism introduced in the PADDL model is critical to account for the empirical data.

It is conceivable that rather than proactive control, participants could instead be reactive [49] and instead of adjusting the initial height of the boundary, they might wait for evidence accumulation to detect conflict (e.g., among multiple items in WM), and then reactively adjust the boundary (e.g., [48]). While such models are typically difficult to estimate, our differentiable likelihood neural network methods rendered their estimation straightforward [38]. We tested this idea by allowing a dynamic decision boundary which collapsed over time (increasing the probability of responding), but where this collapse could be delayed or reduced when conflict (from the higher WM load in higher set sizes) is detected [48]. We thus augmented the LBA decision process model to allow for a collapsing decision threshold with an angle θ. Posterior distributions (see Table A in S1 Text) however suggested that this angle was close to zero in both groups, and models that allowed this collapse to change with set size also worsened fit, further supporting a proactive rather than reactive control account. This result is sensible here because WM load was blocked and hence participants could adopt a proactive strategy to accommodate the need for more evidence accumulation across the entire block; in contrast, previous findings of conflict-induced changes in collapsing bounds applied in perceptual tasks whereby task condition was unpredictable over trials [48].

### PADDL-estimated RL predicts out-of-sample test phase choices

The variant of the RLWM task used here [23,42] includes a test phase administered after the learning blocks/train phase, in which participants choose the more rewarding option between pairs of previously learned stimuli without feedback. Because the paired stimuli are drawn across different learning blocks and are encountered after long delays, working memory makes no appreciable contribution at test, and choices are instead governed by the slowly accrued reinforcement learning values, thought to reflect striatal learning [15,50]. The test phase therefore provides a WM-free read-out of retained stimulus-reward values, and a stringent out-of-sample criterion: the learning rate was estimated entirely from the train phase, so test phase performance was never part of the fitting objective. Moreover, performance was never evaluated on the test phase until final model comparison and selection was performed on the learning data.

Because the test phase is thought to reflect RL contributions, we evaluated whether the RL learning rates estimated from PADDL and choice-only models were more predictive of test phase accuracy. For each participant, we extracted the final Q-values from each model at the end of learning, which are fully determined by the history of choices and outcomes and the RL learning rates (α, γ). Because participants were asked which stimulus was more rewarded (not which response was rewarded), Q-values were weighted according to the frequency of the chosen action, such that each stimulus had an effective action-independent reward value. (Equivalently, these values can be obtained by updating a V-value for each stimulus based on its (stimulus-specific) reward history during learning, ignoring the response selected, but using the same RL learning rate, akin to a “critic” in actor-critic models). We then predicted test phase choices from these values, binned by the within-participant value difference between the two offered stimuli, and compared predicted to empirical accuracy. No parameters were estimated on the test phase data, and an identical softmax choice rule was applied for simulating test phase choices in both models (it is no longer sensible to use the LBA for the test phase; see Methods), so the only quantity differing between the two sets of predictions was the learned values each model implied.

Strikingly, only the PADDL model accorded well with test phase choices, and the two models diverged in a diagnostic way. Supporting the conclusion that it overestimated RL learning rates, the choice-only softmax model systematically overpredicted test accuracy, with predictions exceeding the 94% HDI of the empirical estimates at the medium and high value-difference bins and the discrepancy growing with value difference (Fig 4A). Its psychometric function was correspondingly too steep through the point of indifference (Fig 4B), the signature of over-separated learned values. The PADDL-derived values, in contrast, tracked the empirical curve closely, with only a slight underprediction at the largest value difference, substantially smaller than the softmax overprediction. Quantifying this as the signed deviation of predicted from empirical accuracy, the softmax model was substantially biased upward (mean deviation = 0.0488, 95 % bootstrap CI [0.0313, 0.0657]) whereas PADDL was approximately unbiased (mean deviation = −0.0190, 95% bootstrap CI [−0.0384, −0.0001]).

**Fig 4:**
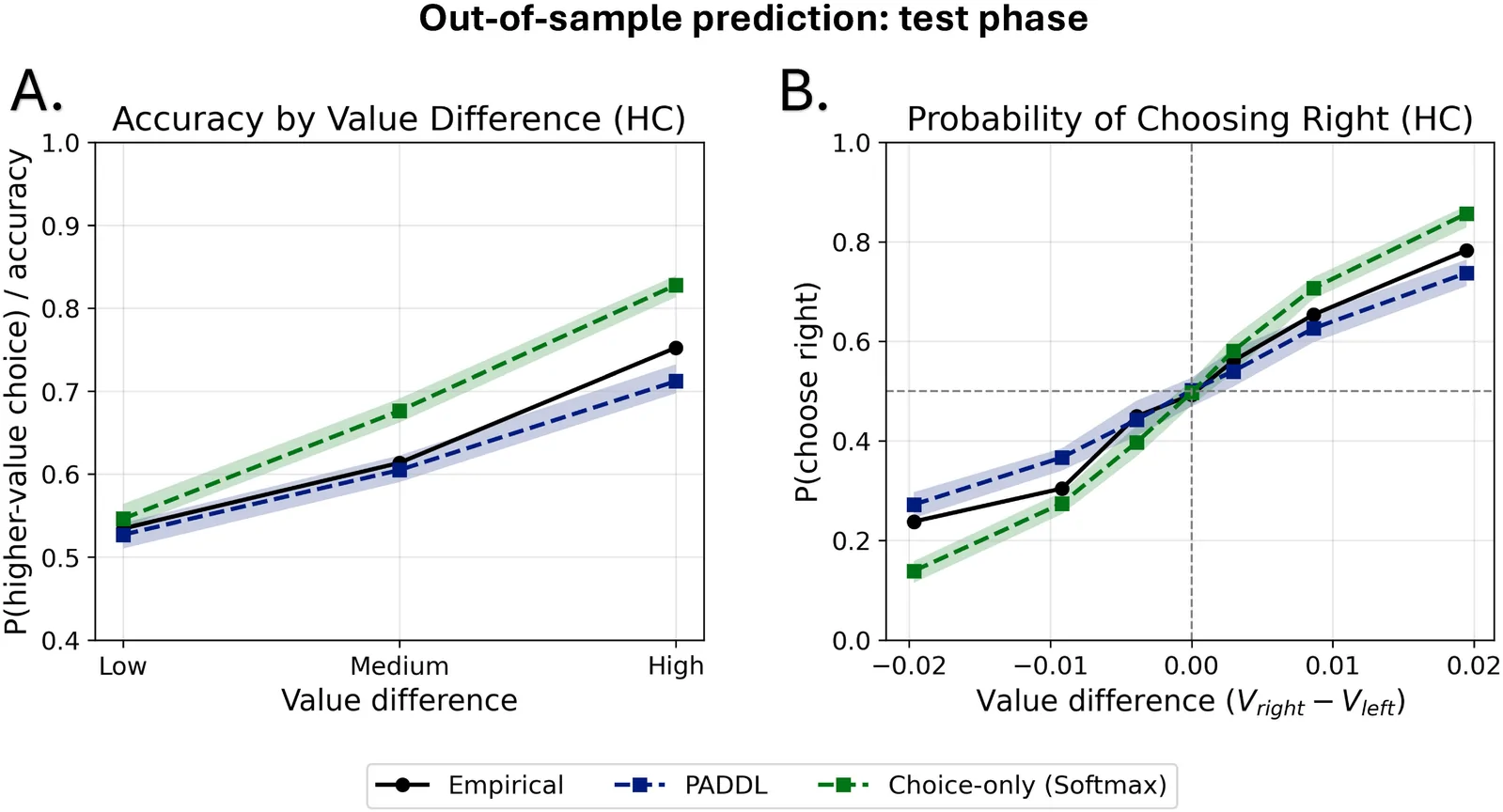
Out-of-sample validation of estimated parameters on the held-out test phase (HC group). Test phase choices were predicted from end-of-training RL values obtained from each participant’s learning phase, with no parameters fit to test data and an identical softmax rule applied to both models, so predictions differed only in the learned values each implied (see Methods). **(A)** Probability of choosing the higher-valued stimulus by within-participant value-difference tertile, for empirical data (black), PADDL (blue), and the choice-only softmax model (green). The choice-only model overpredicts accuracy, exceeding the empirical estimates at the medium and high bins, whereas PADDL tracks the empirical curve closely. **(B)** Psychometric function of choosing the right-hand stimulus against the signed value difference ($V_{\text{right}}-V_{\text{left}}$). The choice-only curve is too steep through indifference, the signature of over-separated learned values, while PADDL matches the empirical slope. Shaded bands denote 94% HDI.

To statistically test whether PADDL and the choice-only softmax model translate their internal predictions into empirically accurate choice probabilities during the test phase, we conducted a calibration analysis. This paired prediction analysis places both models in one fully interacted logistic calibration regression (see details in Methods). PADDL’s calibration slope was 1.1179 (95% CI [0.8969, 1.3390]), consistent with ideal calibration (slope = 1). The choice-only softmax slope was 0.6625 (95% CI [0.5527, 0.7722]), indicating overconfident probabilities. The direct choice-only-minus-PADDL slope difference was −0.4554 (95% CI [−0.5895, −0.3212], cluster-robust $p=2.87\times 10^{-11}$). Calibration intercepts did not differ. Thus, PADDL’s predicted choice probabilities closely matched participants’ observed behavior, whereas the softmax model was systematically overconfident, assigning probabilities that were more extreme than the data supported. Therefore, PADDL more accurately captured the strength of participants’ choice preferences. (When averaged across all test trials, the participant-level log loss favored PADDL only numerically and did not reach significance (difference 0.0048 ± 0.0067 SEM), but this analysis includes many trials in which RL value differences are small and the two models agree). The better calibration of the PADDL model is the direct out-of-sample consequence of the overestimation documented earlier (Figs 2 and 3): an inflated learning rate produces overly separated learned values that overstate discriminability at test. That PADDL predicts the held-out test phase accurately using parameters estimated solely from the learning phase indicates that its lower learning rate estimates reflect the true incremental RL signal rather than under-identification, and provides independent support for the learning rate estimates within-group (see section Test phase results for SCZ) (Fig C in S1 Text).

### Proactive Adaptation of Decision Dynamics in Learning (PADDL) model reveals differences in WM, RL and proactive control across clinical groups

Having established that the proactive control model improves parameter recovery and identifies novel neurocognitive processes, we next set out to determine whether it affords new conclusions about clinical differences between HC and SCZ groups. Previous RLWM studies suggested that SCZ patients show impaired WM but intact RL [19,23,44].

To understand the behavioral differences across clinical groups in terms of the cognitive parameters of the computational model, we compared the means of the group-level posterior distributions obtained from the parameter fitting procedure. We leverage HDI as the Bayesian test of credible difference between the two distributions (see Methods section Clinical group contrasts for details). Fig 5B shows three key findings from parameter estimation. First, replicating prior findings, the SCZ group showed higher rates of WM forgetting/interference as estimated by the WM decay parameter ϕ ($\mu_{\phi }^{HC}$ = 0.0964, $\mu_{\phi }^{SCZ}$ = 0.3658, $\phi_{contrast}$ HDI = [−0.3921, −0.1196]). While the SCZ group also had numerically reduced WM capacity, we did not find credible differences between the two groups ($\mu_{C}^{HC}$ = 2.6940, $\mu_{C}^{SCZ}$ = 2.0537, $C_{contrast}$ HDI = [−1.1827, 2.5255]). Note, however, that the larger ϕ reported above does reduce *effective* capacity.

**Fig 5:**
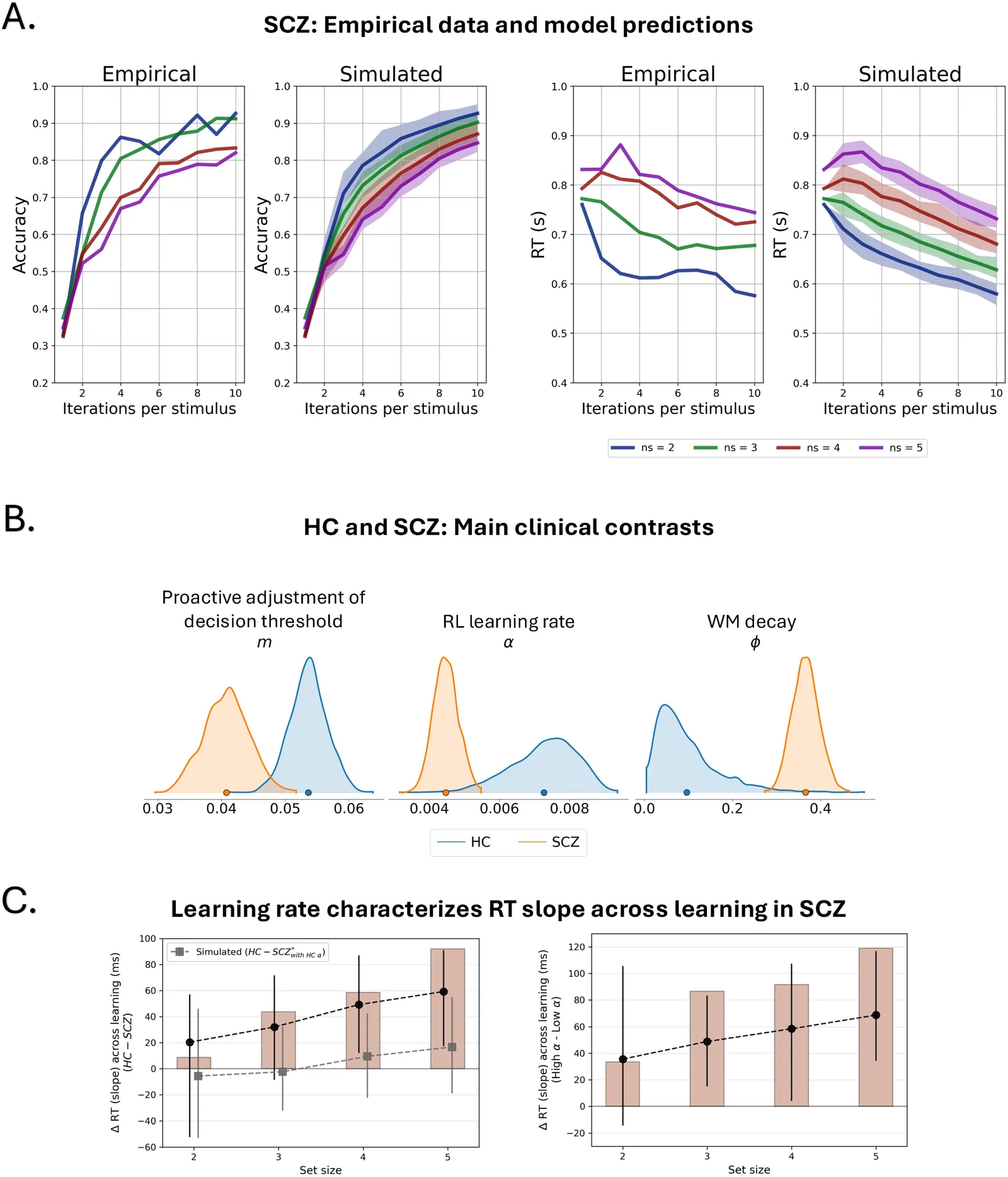
PADDL reveals RL, WM and proactive control differences between HC and SCZ. **(A)** Posterior predictive checks for SCZ. *(Left)* Learning curves for each set size plotted in terms of accuracy for empirical data and model simulations. The model simulations were generated by sampling the posterior and using the post hoc absolute fit method. *(Right)* Learning curves for each set size plotted in terms of RT trends for empirical data and model simulations. Shaded regions show 94% HDI ranges of the group mean. **(B)** Group-level differences between HC and SCZ. Posterior distributions of the group-level means are shown for the three main contrasts – proactive adjustment of decision threshold *m*, RL learning rate α and WM decay ϕ. Note that α denotes the back-transformed (via *exp* function) parameter space. **(C)** *(Left)* RT differences (or slope) in empirical and simulated data between HC and SCZ groups over the course of learning. The RT slopes across learning are characterized by learning rates – HC group with higher learning rate shows steeper slope than SCZ. The differences in slope increase with set size. The trend in gray shows the same effect on simulated data when SCZ learning rate is adjusted to match to the HC learning rate. Negating the learning rate differences between HC and SCZ ablates these learning rate induced RT slope effects across learning. *(Right)* RT differences (or slope) in empirical and simulated data between high- and low-α participant subgroups (in HC). The error bars on simulated trends denote 94% HDI ranges.

Second, unlike previous studies using choice-only models, PADDL revealed a lower RL learning rate in the SCZ group than in controls ($\mu_{\alpha }^{HC}$ = 0.0073, $\mu_{\alpha }^{SCZ}$ = 0.0045, $\alpha_{contrast}$ HDI = [0.0009, 0.0046]). In our previous study [44] with the same dataset, we found that fitting a choice-only model revealed differences only in WM decay (ϕ), but not in the RL learning rate (α). Using summary statistics on empirical and simulated datasets from HC and SCZ, we confirmed that the model was able to account for a relatively slower rate of decrease in RTs over the course of learning (i.e., less steep RT slopes) in the SCZ group than in the HC group (Fig 5C). The difference is more pronounced in higher set sizes because of a more prominent RL contribution. The model is able to account for these group differences in RT slopes owing to accurate characterization of the RL learning rate – removing HC-SCZ learning rate differences cancels out these RT slope effects (Fig 5C). A similar analysis of high- and low-α participants in the HC group revealed that the RL learning rate accounts for these differences in RT slopes. Overall, these conclusions were afforded by the better parameter recovery and identifiability of the RL learning rate enabled by our PADDL model, as fitting with the choice-only model again failed to reveal a difference in learning rate between SCZ and HC. As shown above (Fig 3A, 3B and 3D), the appropriate RL learning rate accounts for more incremental speed-ups in RTs over learning. Indeed, Fig 5A and 5C show that SCZ patients showed more gradual declines in RTs over trials compared to HC.

Third, SCZ patients showed reductions in proactive adjustment of the decision boundary as a function of WM load ($\mu_{m}^{HC}$ = 0.0536, $\mu_{m}^{SCZ}$ = 0.0408, $m_{contrast}$ HDI = [0.0049, 0.0218]). Qualitatively, this effect can be observed by inspecting the RTs during early learning trials: HC participants show more pronounced increases in RT with higher set sizes than do SCZ (Fig 5A). This result reveals a novel computational difference between SCZ and HC in the RLWM paradigm, but is generally consistent with prior literature suggesting cognitive control and performance monitoring deficits in SCZ [51,52].

## Discussion

We introduced a novel computational framework that makes fitting joint choice and response time models of learning computationally feasible, overcoming a long-standing methodological barrier. Using this framework, we provide the first mechanistic account of how individuals adapt to cognitive load during learning by proactively widening their decision boundary and show that a failure in this specific dynamic adjustment is a potential marker for learning deficits in clinical populations. Using our joint PADDL model, we identified specifically that this mechanism is altered in patients with schizophrenia. Moreover, the enhanced ability to disentangle WM from RL processes further identified a reduction in patients’ RL learning rate which had been masked by prior studies using choice-only models [19], including with the same dataset [44]. We validate this reduction out of sample against the held-out test phase, which the joint model predicts accurately and the choice-only model overpredicts.

Despite RT distributions being informative about the underlying generative processes, almost all previous studies using the RLWM paradigm only model the choice distributions in the data [12,19,23,43,44]. These studies use a choice-only softmax model, despite showing systematic effects of WM load on RT. The RTs bring additional constraints to the model and inform the characterization of the generative RL and decision processes underlying behavior. This is even more critical in the case of the RLWM model because the optimal action is largely convergent between both contributing learning processes. Therefore, the learning contributions from the RL process, governed as a function of the RL learning rate, can sometimes mimic the WM contributions and vice versa [22]. Consequently, we reasoned that modeling RTs would allow us to dissociate parallel and convergent contributions from both processes. For example, in higher set sizes under high WM demand, the relative contribution of the RL process increases, resulting in a gradual speeding of RTs over learning. A choice-only model, uncoupled from the RT constraints, would mischaracterize the RL contribution by overestimating the RL learning rates. The posterior predictives, instantiated as post hoc absolute fits, confirmed this by showing that the choice-only softmax model predicts progressively faster RTs in higher set sizes as learning progresses compared to the PADDL model. The choice-only model overestimates the learning rate, WM reliance, and WM capacity and underestimates the WM decay and perseveration parameters as compared to the PADDL model. We confirm that such a bias could emerge because choice-only models do not have the constraints from the RT distributions, especially the tail of the RT distribution that restricts the drift rates of the accumulators in the decision process. The net effect of the overestimation of learning (due to higher learning rate, WM reliance, and WM capacity) would result in an increase in drift rates. This interpretation is confirmed by follow-up parameter recovery studies where we recover the data-generating parameters for the synthetically generated PADDL model data. The recovery with the choice-only model shows a similar pattern of overestimation of learning rate, WM reliance, and WM capacity alongside underestimation of noise, WM decay and perseveration parameters, suggesting that the PADDL model better characterizes the learning processes in the brain. Our findings extend previous work [31,33,35,45,53] in showing that parameter identifiability and recovery improve when choices and RTs are jointly modeled in complex cognitive models with parallel learning systems.

In this work, we improve upon the model proposed in [18], hitherto the only work attempting joint modeling of the RLWM task. We address the key limitations of the previous work and hypothesize two additional speed-accuracy trade-offs that could arise at within-trial and between-trial levels. First, using our proposed computational approach, we enable hierarchical Bayesian inference for the RLWM joint model, which has certain methodological advantages over maximum likelihood estimation. In hierarchical inference, the individual participant-level parameters are drawn from group-level distributions, which mutually constrain the estimates at both levels and improve the parameter recovery of individual subjects [54]. Moreover, Bayesian inference quantifies uncertainty over the model parameter estimates, a critical advantage in the computational investigation of clinical group differences. Second, their model was unable to capture the participants’ RTs during the initial phase of learning. Since the model initializes all action values uniformly and does not incorporate an explicit mechanism to predict RTs across different set sizes, the model does not capture the set size-based RT differences observed in the empirical data. Therefore, we hypothesized a speed-accuracy trade-off operating at an across-trials timescale that could potentially influence the decision dynamics depending on factors such as cognitive or WM load. As task difficulty increases in higher set sizes when WM demands overwhelm WM capacity, such a proactive speed-accuracy mechanism could regulate the parameters of the decision process dynamically depending on the set size to address the need for more accurate responding. Specifically, we hypothesized that the decision boundaries are modulated as a linear function of the WM load (or set size).

Our hypothesis can be construed as a mechanistic implementation of proactive cognitive control in the context of the RLWM task. Proactive control is characterized by the anticipatory maintenance of goal-relevant information to prepare for expected cognitive demands [49]. In this context, when participants are informed of the current task context (set size) by the cue at the beginning of each block, they anticipate increased difficulty in higher set sizes and engage in a preparatory state. This proactive adjustment translates directly into a specific, low-level adjustment within the decision process. By proactively increasing the decision threshold, the evidence criterion for a choice rises, effectively prioritizing accuracy over speed. In higher set sizes, this strategic slowing is a canonical hallmark of proactive control, which is particularly effective for ensuring accurate performance in anticipated high WM demand or high-difficulty task contexts.

Our finding that proactive control is implemented via an increased decision threshold is consistent with evidence from other cognitive paradigms. For instance, in task-switching paradigms, performance on difficult task-switch trials is characterized by raising the evidence boundary to prevent errors due to high working memory load and interference effects [55,56]. Likewise, prospective memory tasks are also characterized by increased decision bounds [57,58], which are dynamically adjusted according to the specific demands of the secondary task [59]. A recent study examining neurocomputational underpinnings of subjective effort found that participants proactively increase the decision bounds based on anticipated difficulty of trials [60]. These convergent findings suggest that adjusting the decision threshold is a general-purpose mechanism for implementing proactive control in response to anticipated cognitive load or interference effects. The neural circuit-level mechanism responsible for adaptively increasing the decision bounds to maintain accuracy in the face of difficult decisions is thought to relate to connectivity between the dorsomedial prefrontal cortex, including the anterior cingulate cortex (ACC), and the subthalamic nucleus [27,47,48]. The finding of reduced proactive control in SCZ is also convergent with alterations in performance monitoring and ACC function in this population [51,61,62].

The modeling results in this work provide new insights into how the learning behaviors manifested via RL and WM interactions go awry in clinical groups. In the SCZ group, in line with previous results, we found evidence for lower WM capacity and faster WM decay, implicating profound deficits in WM that dominate learning impairments in SCZ [19,44]. These previous studies found no evidence for deficits in the RL learning rate. With the joint modeling approach, we were able to unravel the cause of less optimal learning in SCZ and identify a reduction in the RL learning rate in addition to the WM deficits. Our analysis further confirmed that this reduction in learning rate explains less steep RT slopes over the course of learning in SCZ than in HC – a previously undescribed RL signature that could be identified empirically, even within HC with high- and low-learning rate subgroups. These deficits in the SCZ group could in principle stem from the broadly reduced cognitive functioning in that group. We note, however, that such functioning is itself composed of separable primitives spanning RL, WM, and cognitive control, and that the PADDL model is intended to identify which of these are affected. Here, reduced RL learning rate, weakened proactive control, and faster WM decay emerge as the specific contributors, while capacity, perseveration, and noise remain intact (i.e., no credible difference is found), indicating a selective rather than global pattern.

Our study isolated the blunting of RL contributions via joint modeling of choice and RT data. This closes a methodological loop that has long shaped this literature. Historically, the slow incremental RL contribution was difficult to observe during learning because the fast WM system dominates trial-to-trial behavior, and it was recovered instead from a separate test phase administered after learning [15], an observation that motivated the RLWM design in the first place [12]. The present results show that the joint model recovers this slow contribution from the learning phase itself, with the test phase now serving as independent, out-of-sample confirmation rather than as the primary means of identification. Because the test phase indexes retained value with negligible WM involvement, the fact that the train phase parameters accurately predict test phase choice accuracy, whereas the choice-only model overpredicts it, provides converging evidence that the joint estimates reflect the true incremental RL contribution rather than under-identification. The calibration results on the test phase suggest that PADDL better captures the strength of participants’ choice preferences, whereas the softmax model produces systematically overconfident predictions. This calibration advantage may reflect a more realistic representation of behavioral uncertainty. The reduced RL learning rate in schizophrenia is supported by three converging lines of evidence: improved parameter recovery under the joint model, an RT-slope signature across set sizes that is abolished when the group learning rates are equalized, and out-of-sample prediction of the held-out test phase. Moreover, our finding that individuals with SCZ exhibit a reduced RL learning rate is consistent with a large literature implicating alterations in striatal dopamine signaling in these patients [63]. A reduced RL learning rate may also relate to negative symptoms due to the blunted dopamine transients for relevant stimuli in learning [63,64]. The reduction in learning rate could reflect dopamine dysregulation in the striatum either as a result of the disorder or due to the antagonist effect of the antipsychotic treatment (which blocks dopamine in the striatum). The resulting computational deficit reflects impaired reward prediction error signaling, which mechanistically leads to reduced ‘Go’ learning [63].

Our current findings also show substantial agreement with our previous work ([44]; see Supplement for group differences in the fitted model parameters) despite some differences in the model and the fitting procedure. Our previous work showed that there were differences in neural signatures of RL and WM in the clinical groups that were not observable from behavior or parameters estimated from model fitting alone, whereas in this work, we are focusing on how modeling of RTs can help inform processes without neural measures. As compared to the previously employed RLWM choice-only model, the PADDL model revealed additional differences in the learning rate and proactive control. Our previous study reported that both BP and SCZ showed more WM decay than HC and MDD, and our current study also found a similar difference in which HC showed lower WM decay than SCZ.

Technically, our proposed computational approach extends previous likelihood-free inference approaches [37,65–67] in dealing with a complex class of hybrid models where a computationally expensive trial-by-trial process informs the parameters of the decision process generating the observed variables – choice and RT. We combine a differentiable implementation of the learning process with the amortized likelihoods for the decision process to enable efficient likelihood evaluation as well as gradient computation of the likelihood with respect to the model parameters. This allows the use of efficient gradient-based Markov Chain Monte Carlo (MCMC) samplers (such as the No-U-Turn Sampler) and other optimizing techniques (such as VI) that allow approximating intractable probability densities. These gradient-based inference methods are more efficient than other MCMC approaches (such as Metropolis-Hastings or Slice sampling) that do not use the gradients for efficiently traversing the posterior space. In practice, this enables a Bayesian treatment of a variety of otherwise computationally expensive likelihoods and high-dimensional, hierarchical model definitions. In addition, the proposed approach is modular and extensible to a broad class of cognitive models. The amortized likelihood (or Likelihood Approximation Network (LAN)) for the decision process can be readily swapped with the likelihood function for other candidate decision processes to test alternative hypotheses regarding the underlying choice mechanisms. Similarly, alternative accounts of the learning process could be tested without the need to retrain the amortized decision process likelihoods. Beyond the kind of models considered in this article, the computational approach naturally extends to other classes of cognitive models that describe across-trial dynamics of decision making in terms of cognitive control, affect, etc. In sum, our approach allows testing and validation of cognitive theories that jointly account for complex across-trial learning dynamics alongside within-trial decision dynamics.

Furthermore, our choice of using a linear ballistic accumulator with collapsing bounds was deliberate, for two reasons. First, to address a limitation of the previous work [18] in capturing faster error RTs, we hypothesized an urgency-related signal that could be accounted for by a collapsing bound evidence accumulation process. This would enable the model to make a decision with a lesser amount of accumulated evidence as time progresses, thus accounting for the faster error RTs in the data. Second, the analytical likelihood function for the linear ballistic accumulator with collapsing bounds has not yet been derived, and so our approach illustrates the generality of using surrogate likelihoods (via LANs) in conjunction with differentiable RL likelihoods. Future work can build upon this to address a few limitations in computational modeling. Further studies can explore other alternative decision process models that take into account within-trial noise in evidence accumulation [68], leakage and inhibition in accumulators [69] or a neurally inspired diffusion process [70]. Our proposed computational approach no longer limits the choice of decision process models based upon the availability of methodologically convenient analytical likelihoods. Moreover, the model predicts a less steep decrease in RT in terms of stimulus iterations than empirically observed in lower set sizes. In higher set sizes, the model does not predict an initial increase in RTs before it decreases with stimulus iterations. Taken together, this suggests that the model fails to incorporate this cognitive aspect responsible for the distinctive slope of RT trends in lower and higher set sizes. A potential way to better account for these trends would involve explicit modeling of WM management by modeling common recency/primacy biases or participant-specific strategies. Exploring other non-linear interactions between set size and the WM module might also help capture the individual set size RT trends.

In conclusion, a complete understanding of human learning requires moving beyond models that focus solely on choice outcomes. Our work demonstrates the necessity of incorporating the decision process itself, providing a generalizable computational framework that jointly models both what choices are made and how they are made by accounting for response times. This work provides both a generalizable computational approach for joint modeling of choice and RT distributions as well as a clear demonstration of its value, revealing a fundamental mechanism of cognitive control that is crucial for adaptive behavior under high cognitive load. Such an integrative approach, combining methodological and theoretical advances, could be leveraged to accurately characterize the neurocognitive mechanisms of learning and reveal important contrasts across clinical groups to understand how these learning mechanisms go awry in clinical conditions.

**Box 2. Glossary of key concepts**

**Table:** 

Concepts	
RLWM paradigm	A task and model that vary memory load (set size, delay) so that a fast, capacity-limited working-memory (WM) system and a slow, incremental reinforcement-learning (RL) system make separable predictions, allowing their contributions to be estimated rather than confounded.
**Reinforcement learning (RL)**	Gradual, reward-prediction-error-driven strengthening of stimulus-response values across trials. Incremental and relatively slow learning process.
**Working memory (WM)**	Rapid but capacity-limited and decay-prone maintenance of recent stimulus-response associations. Contributes to rapid behavioral flexibility.
**Linear ballistic accumulator (LBA)**	A decision model (without within-trial noise) in which one independent accumulator per response option races to a threshold; the first to reach it determines the choice and the response time.
**Collapsing-bound LBA or LBA Angle**	An LBA variant with a linearly collapsing (“angled”) decision boundary, so the threshold declines within a trial, capturing time-dependent urgency.
**Proactive control**	A cognitive control mode that implements anticipatory adjustment of the decision process; here, widening the decision boundary under higher WM load to trade speed for accuracy.
**Simulation-based inference (SBI)**	A family of Bayesian methods that fit models lacking a closed-form likelihood by learning the likelihood (or posterior) from simulated data.
**Likelihood Approximation Network (LAN)**	A neural network trained on simulations to approximate the likelihood of an otherwise intractable decision model (here, the LBA Angle), enabling fast gradient-based inference.
**Hierarchical ADVI**	Automatic Differentiation Variational Inference: gradient-based approximate Bayesian inference that recasts posterior estimation as optimization. “Hierarchical” jointly estimates individual- and group-level parameters, with group-level priors regularizing individual estimates; “full-rank” preserves posterior correlations.
**Savage-Dickey density ratio**	A method to obtain a Bayes factor for a point hypothesis in a nested model (e.g., a parameter equals 0) as the ratio of posterior to prior density evaluated at that point; it quantifies evidence for or against the parameter being zero.

## Methods

### Ethics statement

The experimental procedures were approved by the Institutional Review Board at Washington University in St. Louis. All participants gave informed written consent prior to participation.

### Participants

This study was conducted as part of the Cognitive Neuroscience Test Reliability and Clinical Applications for Schizophrenia Consortium (CNTRaCS). The participants were recruited from five different US locations – the University of Maryland, the University of California, Davis, the University of Minnesota, the University of Chicago, and Washington University in St. Louis. As part of this larger study, we collected data for a total of 255 participants across the four diagnostic groups (HC = 87, SCZ = 67, MDD = 54, BP = 47). This dataset also included simultaneously recorded EEG. No participants were excluded within any analyzed group.

Diagnostic status across all study groups was determined using the Structured Clinical Interview for DSM-5, as detailed below. All patient participants were clinically stable, with no medication adjustments within the preceding month and none anticipated in the subsequent month. Individuals with MDD satisfied DSM-5 criteria for a minimum of two depressive episodes, with at least one episode occurring within the prior three years. Participants with SCZ were diagnosed with either schizophrenia or schizoaffective disorder, based on the SCID assessment described below. HC participants had no current psychiatric disorder, were not using psychiatric medication, and reported no family history of psychosis. All individuals were between 18 and 63 years, had no history of neurological injury, and met criteria for the absence of substance use disorder during the three months preceding study enrollment.

A detailed analysis of the behavioral and EEG data across clinical groups is reported in our previous work [44]. The main focus of this study is on the behavioral data from HC and SCZ groups because multiple studies in the past have contrasted these groups where SCZ deficits were shown to be limited to WM contributions. However, for completeness, we also report effects in the other populations (BP and MDD) in the supplementary – see section Clinical results for BP and MDD (Fig D and Table B in S1 Text). These groups required EEG-based phenotyping to dissociate specific neurocomputational mechanisms underlying performance deficits [44].

### Diagnostic assessment

All participants completed the Structured Clinical Interview for DSM-5 (SCID-5) with a trained researcher under the supervision of a doctoral-level clinician. Raters received training through remote webinars in which scoring procedures and anchor points were reviewed, and they also completed six standardized training videos followed by group discussions. After training, raters were required to reach agreement with “gold standard” ratings provided by experienced clinicians at the Maryland and St. Louis sites across at least six practice interviews. Agreement was defined as having no more than two items differing by more than one point from the reference ratings. To ensure reliability throughout the study, the St. Louis site recorded one interview every 2–4 weeks, which all raters evaluated. These were subsequently discussed in remote consensus meetings to resolve discrepancies and maintain inter-rater consistency.

### Reinforcement Learning - Working Memory (RLWM) task

The RLWM task [12,19] was developed to dissociate the respective contributions of reinforcement learning and working memory to stimulus-response acquisition. In this paradigm, participants acquire stimulus-response associations through trial-and-error learning within a three-alternative forced-choice design. Correct responses always yielded positive feedback, with reward magnitude taking a value of 1 or 2 points; incorrect responses yielded 0 points. The overall structure of the task is divided into two distinct phases: a learning phase and a test phase.

During the learning phase, participants complete multiple trial blocks in which WM demands are parametrically manipulated. These manipulations occur through variation of the set size – the number of unique stimuli per block, ranging from two to five, as well as through the manipulation of the delay, defined as the number of intervening trials before a stimulus is re-presented. These design features allow for systematic evaluation of WM-related learning by varying WM demands both within and across blocks. In contrast, the RL-related contributions to performance are reflected in progressive improvements in accuracy that accumulate as a function of reward history, reflecting the gradual strengthening of stimulus-response associations. In total, participants completed ten training blocks with varying set sizes, amounting to 360 trials overall. By jointly examining performance under varying WM loads and accuracy improvements driven by reinforcement history, the RLWM task offers a structured framework for parsing the unique and interactive effects of RL and WM on learning processes.

The subsequent test phase is intended to measure longer-term reward retention or value-based learning effects. We do not include the test phase as a constraint during model fitting, since parameter estimation can be conducted accurately using the learning phase data alone (see Computational Modeling). We instead reserve the test phase as an independent, out-of-sample validation of the fitted learning rates (see Results and Methods), for which it is well suited because working memory contributes negligibly to retention across blocks. However, we do note that our prior work has not identified clinically relevant behavioral differences in the test phase [44].

### PADDL model: Joint computational model of choice and RT

The RLWM model was utilized to characterize learning as the joint outcome of two concurrent mechanisms: reinforcement learning and working memory. This computational framework was applied to participants’ empirical data, with model fitting performed on both choice behavior and response time distributions.

Conceptually, the RLWM model integrates two systems that operate in parallel, each contributing uniquely to behavior. The WM system supports rapid acquisition of associations but is constrained by limited capacity and temporal decay, whereas the RL system provides slower yet more stable learning that enables long-term retention. Both mechanisms independently encode state-action value representations, which are updated through temporal-difference learning rules. To capture the characteristic one-shot updating of WM, its learning rate is fixed at 1, reflecting immediate encoding but without the iterative adjustments typical of RL. WM capacity is represented by the parameter *C*, which provides a probabilistic estimate of the number of active “memory” slots or capacity available at a given time. The natural decline of WM representations is modeled by a decay parameter ϕ, capturing the process of information loss across trials. The mixture decision policy is determined by a weighted integration of the RL and WM policies, with the parameter ρ indexing the individual’s general propensity to rely on the WM process over the RL process for decision-making.

**Temporal-difference updates in RL and WM processes.** Eqs. 1 and 2 define the trial-by-trial updating rules for the RL and WM components, respectively. In these formulations, *s* refers to the current trial’s stimulus or state, *a* denotes the action chosen, and $r_{t-1}$ indicates the reward outcome obtained on that trial. The RL component is further parameterized by α, the learning rate that governs the rate of incremental updating, and by γ, a perseveration parameter that captures the tendency to ignore negative feedback. For the purposes of model implementation, reward outcomes were binary coded, with 0 corresponding to an incorrect response and 1 to a correct response. Our prior analysis [17,23] has demonstrated that incorporating a three-level reward coding scheme (0–1–2 points) within the Q-learning updates did not improve model fit relative to the simpler binary coding. (The reason is probably that the participants know there is only one correct response for each stimulus, so the best they can do is find that rewarding response.)


$$\begin{array}{c} Q_{RL}(s,a{)}_{t}=\left\{ \begin{array}{cc} Q_{RL}(s,a{)}_{t-1}+\alpha \cdot (1-Q_{RL}(s,a{)}_{t-1}) & \text{if }r_{t-1}=1\\ Q_{RL}(s,a{)}_{t-1}+\alpha \cdot \gamma \cdot (-Q_{RL}(s,a{)}_{t-1}) & \text{if }r_{t-1}=0 \end{array}\right. \end{array}$$ (1)

$$\begin{array}{c} Q_{WM}(s,a{)}_{t}=\left\{ \begin{array}{cc} 1 & \text{if }r_{t-1}=1\\ Q_{WM}(s,a{)}_{t-1}+\gamma \cdot (-Q_{WM}(s,a{)}_{t-1}) & \text{if }r_{t-1}=0 \end{array}\right. \end{array}$$ (2)

**WM decay.** The model accounts for WM decay by progressively reverting the WM Q-values toward their baseline initialization of $1/n_{a}$, where $n_{a}$ denotes the total number of available actions. This decay process is parameterized by ϕ (Eq. 3), which quantifies the extent to which intervening trials disrupt or interfere with WM representations before the same stimulus is presented again.


$$\begin{array}{c} Q_{WM}(s,a{)}_{t}=Q_{WM}(s,a{)}_{t-1}+\phi \cdot (\frac{1}{n_{a}}-Q_{WM}(s,a{)}_{t-1}) \end{array}$$ (3)

**Behavioral policy formulation.** On each trial, the Q-values generated by the RL and WM components are converted into action choice probabilities through a softmax transformation, as specified in Eq. 4. The expression *p*(*a*|*s*) gives the individual probability mass assigned to each action. The tuple $\left[p_{a1},p_{a2},p_{a3}\right]$ is individually derived to get RL and WM policies. This function incorporates an inverse temperature parameter, β, which was fixed at 100 for all participants in order to minimize potential confounds and avoid undesirable trade-offs with other free parameters of the model.

The resulting policies, $\pi_{RL}$ and $\pi_{WM}$, are then combined into a weighted mixture policy, $\pi_{mix}$. The relative weight of this mixture depends on the degree of WM engagement in a given block, represented by ω, whose computation is described in Eq. 5. The parameter ρ reflects a participant’s baseline tendency to rely on WM across different task contexts, while *C* denotes the individual’s WM capacity. The variable $n_{delay,k}$ corresponds to the number of unique, intervening stimuli encountered since the last correct response for any given stimulus *k*. It is important to note here that delay, measured since the last correct response, scales with set size. Finally, Eq. 6 specifies how the combined policy $\pi_{mix}$ is derived as a weighted integration of $\pi_{RL}$ and $\pi_{WM}$.


$$\begin{array}{c} p(a|s)=\frac{\exp(\beta \cdot Q(s,a))}{\sum_{i}\exp(\beta \cdot Q(s,a_{i}))} \end{array}$$ (4)

$$\begin{array}{c} \omega =\rho \cdot \min(1,\frac{C}{n_{delay,k}}) \end{array}$$ (5)

$$\begin{array}{c} \pi_{mix}=(1-\omega )\cdot \pi_{RL}+\omega \cdot \pi_{WM} \end{array}$$ (6)

**Incorporation of undirected noise.** The final evaluation policy for the train phase, $\pi_{train}$, is obtained by combining the mixture policy with a uniform policy that assigns equal probability to all possible actions. This addition accounts for random noise or attentional lapses that may be reflected in participants’ behavioral data. The parameter ϵ specifies the extent to which such noise or lapses influence decision-making. Eq. 7 formalizes the computation of the final behavioral policy following the integration of these noise-related probabilities.


$$\begin{array}{c} \pi_{train}=(1-\epsilon )\cdot \pi_{mix}+\epsilon \cdot (\frac{1}{n_{a}}) \end{array}$$ (7)

**Evidence accumulation decision process.** The decision-making process is formalized through an evidence accumulation framework. Building on prior work [18], our approach replaces the standard softmax choice rule with a linear ballistic accumulator, allowing simultaneous modeling of choices and RTs. In the LBA, each of the three possible actions is represented by an independent accumulator, with accumulation rates proportional to the action values derived from the RL and WM processes. A decision is registered once one of the accumulators reaches its boundary. Because both RL and WM values evolve dynamically across trials, the associated RTs also shift over time. Additional trial-to-trial RT variability is captured through random variation in the starting points of accumulation.

While the basic LBA provides an analytical likelihood function, for the purposes of both methodological generalization and hypothesis testing, we employ a variant that incorporates linearly collapsing decision bounds (the LBA Angle). This extension allows us to test whether decision thresholds decrease as time progresses and also whether reactive control could be deployed to delay or reduce this collapse [48]. However, because an analytical likelihood function is not yet available for this variant, we employ surrogate likelihoods approximated through LANs [37]. The LAN approach accurately approximates the surrogate likelihood, allowing us to apply a larger class of candidate models without the need for elaborate mathematical derivations for such non-canonical variants of traditional evidence accumulation models. It is important to note that while we employ a model without within-trial accumulation noise (the ballistic LBA), where variability stems from across-trial noise in drift and starting point (as well as learning), our framework and method could be generally applied to a broader class of evidence accumulation models including sequential sampling models such as racing diffusion models.

Within this framework, each action’s accumulator is assigned a drift rate ($v_{0},v_{1},v_{2}$) proportional to the final evaluation policy ($\pi_{train}$). At the start of each trial, accumulation starting points are sampled from a uniform distribution with an upper bound set by the variability parameter (*z*). The first accumulator to cross the decision threshold (*a*) determines both the selected action and the RT. Importantly, the threshold *a* is adaptively modulated by set size (*ns*) on a trial-by-trial basis. This adjustment follows a linear function of threshold intercept (*k*) and threshold slope (*m*), as specified in Eq. 8.


$$\begin{array}{c} a=m\cdot (ns)+k \end{array}$$ (8)

It is worth noting that we initially evaluated a generalized logistic regression function of set size to model the dependence of decision threshold on *ns*. However, we ultimately adopted only the *a*-regression in its linear form, as the parameters recovered from the logistic model indicated that the threshold increased in a manner consistent with a straightforward linear relationship.

To make the inference tractable, we did not estimate the non-decision time in the decision process because it shows severe trade-offs with other learning and decision process parameters [18]. Different parameterizations of the model could be tested to alleviate the issue, or the non-decision time could be estimated by identifying the best-fit parameters over a sparse grid of values.

In summary, the PADDL model is parameterized by the following parameters – α,ϕ,ρ,γ,ϵ,C,m,k,z,θ.

### Model comparison

To perform model comparison, we employed the Savage-Dickey ratio test [71] since the candidate models were nested under the PADDL model. The Savage-Dickey method computes a Bayes factor for a nested (point) hypothesis, such as a parameter being zero, as the ratio of the posterior to the prior density evaluated at that point; a ratio below one favors the parameter being non-zero, and a ratio above one favors it being zero. We examined the two possible nested model alternatives that can account for speed-accuracy trade-offs on a within-trials and across-trials basis. First, we investigated the across-trial trade-offs accounted for by the proactive set size-dependent threshold adjustment. We compare the PADDL model with a Base model (model without the threshold adjustment) by examining the proactive adjustment slope parameter *m* and threshold intercept parameter *k* estimates to check if the introduction of the additional proactive mechanism was better able to account for the choice and RT distributions. Setting *m* = 0 trivially reduces the PADDL model to the Base model. With the PADDL model, the (HC) group-level *m* parameter was recovered with mean = 0.0536 and 94% HDI = [0.0482, 0.0590]. The 94% HDI does not contain 0, suggesting a credible effect of set size-dependency on the decision threshold. We then compared the null hypothesis *H*_0_: *m* = 0 against the alternative $H_{1}:m\neq 0$ using the Savage-Dickey density ratio test ($B_{01}=p(m=0|data)/p(m=0)$). The resulting Bayes factor *B*_01_ < 0.001 confirmed the result indicating strong evidence for the alternative. Second, to examine the trade-offs on a within-trial basis, we compare the PADDL model with an alternative model without a collapsing bound decision process by examining the collapse rate parameter θ. With the PADDL model, the group-level θ parameter was recovered with mean = 0.0007 and 94% HDI = [0.0000, 0.0023]. The Savage-Dickey density ratio test ($H_{0}:\theta =0$, $H_{1}:\theta \neq 0$) indicated strong evidence *B*_01_ = 688.5403 for the null, suggesting that the model without a collapsing bound better describes the data.

### Model fitting

For Bayesian parameter inference throughout this work we relied on Variational Inference (VI) [72]. VI approximates the posterior distribution by optimizing over a parameterized family of candidate distributions so as to minimize the KL divergence (or another divergence measure) from the true posterior. In our framework, the availability of differentiable surrogate likelihoods through LANs, in conjunction with a differentiable RL implementation, makes it possible to compute gradients of the evidence lower bound (ELBO) [73] with respect to the variational parameters. These gradients allow efficient parameter updates using gradient-based VI methods. We elected to use VI rather than MCMC for several reasons: (i) VI generally converges more rapidly than MCMC and often demonstrates more stable optimization behavior, and (ii) VI permits the exploitation of LANs in high-dimensional parameter spaces, thereby enabling a novel and tractable Bayesian inference strategy.

For all model-fitting procedures, we employed Full-rank Automatic Differentiation Variational Inference (ADVI; [74]) as implemented in PyMC [75]. Full-rank ADVI accounts for correlations within the posterior distribution, thereby yielding more reliable and accurate estimates of marginal variances. Parameters were estimated hierarchically with non-informative priors, and the optimization was run for 500,000 iterations with a learning rate of 0.001 using the Adagrad (window) optimizer. Following model fitting, we sampled 2,000 draws from the variational posterior for each parameter. To reduce the risk of converging to suboptimal local minima, each optimization was repeated three times, and the run yielding the highest log-probability was retained for subsequent analyses. Each run was initialized with a randomized starting point in the valid range of parameter space. All runs were similar and yielded similar results in terms of the estimated parameters and resulting posterior predictive checks (Fig H in S1 Text). The ELBO loss was continuously monitored to evaluate both convergence and stability. The same VI settings were applied consistently across all experiments reported in this study.

The likelihood functions were implemented in JAX [76] and wrapped in PyTensor [77] to interface with PyMC [75] for automatic differentiation and numerical computations. To approximate joint choice and RT likelihoods, we trained a multilayer perceptron with five hidden layers (each of size 120), following the procedures introduced in the LAN framework [37]. While the standard LBA has an analytical likelihood, here we relied on a variant with linearly collapsing decision bounds, parameterized by angle θ, which exemplifies a case where LAN-based surrogate likelihoods provide a clear advantage. The resulting neural network takes trial-level data and model parameters as inputs and produces approximate log-likelihood values as outputs. Training was carried out on $5\times 10^{6}$ parameter combinations, with $2\times 10^{5}$ simulations each using an efficient implementation of the LBA Angle model [78], using PyTorch [79].

The non-decision time was set to 150 ms [18]. This assumption helps constrain other parameter estimates and seems reasonable as it likely did not differ across groups because – first, the main clinical differences from our study are about how RTs change over learning and with set size whereas the non-decision time just shifts the entire RT distribution by some offset; and second, the participants from clinical groups did not show slower RTs in the first trial but rather just reduced learning. To avoid undue influence of trials with very fast or slow RTs on the parameter estimates, we masked out trial-wise likelihoods for trials with RTs < 300 ms and those with RTs > 2000 ms. The choice data from these trials were still used to update the state-action values though. Across groups, the proportion of masked trials was ~ 2% (HC: 1.7%; SCZ: 2.7%; BP: 1.6%; MDD: 1.7%).

Examining the posterior pair plots suggested that no severe parameter trade-offs exist at the group level (Fig I in S1 Text).

### Posterior predictive checks

After fitting, the posterior distribution from the best-fit run was sampled to generate 40 simulated datasets. The trial and feedback sequences were fixed to match the empirically observed sequences to control for variance in behavior attributed to different learning trajectories [80]. The behavior on the first stimulus encounter was also fixed to match the empirically observed choice and RT, which cannot be predicted before any learning. The mean and HDI of the group-averaged measures are computed across the simulation runs to denote the confidence in estimating the group-level means.

### Test phase analysis and modeling (Out-of-sample validation)

To validate the recovered parameter estimates from the train phase data against out-of-sample data, we analyzed choices in the test phase, which asked participants to select which of two stimuli had been more rewarding during learning (without feedback). Note that this is a response-independent test; the question is how often this stimulus was rewarded, which is a property of the history of rewards experienced, regardless of whether either stimulus was rewarded for any of the three responses. Because the paired stimuli were drawn from different learning blocks and presented after long delays, the WM system contributes none or negligibly at test, isolating the contribution of the slowly accrued RL values. The test phase was not used during model fitting; all parameters were estimated from the learning phase alone.

For each participant, we reconstructed the end-of-training RL values by re-simulating the trial-by-trial value updates over the observed learning phase sequence using that participant’s fitted learning rates α and γ (which reflects reduced learning from errors). The stimulus values were initialized to chance performance in the three-choice training task. Training trials were then replayed in their observed order. For a trial *t* containing stimulus $s_{t}$ and binary feedback $r_{t}$, the stimulus values were learned using the standard RL update rule, independent of each response, in Eq. 9.


$$\begin{array}{c} V_{s_{t}}^{\left(t+1\right)}=\left\{ \begin{array}{cc} V_{s_{t}}^{\left(t\right)}+\alpha \cdot (r_{t}-V_{s_{t}}^{\left(t\right)}) & \text{if }r_{t}=1\\ V_{s_{t}}^{\left(t\right)}+\alpha \cdot \gamma \cdot (r_{t}-V_{s_{t}}^{\left(t\right)}) & \text{if }r_{t}=0 \end{array}\right. \end{array}$$ (9)

In sum, we extracted the final learned stimulus values using the same update equations from the learning phase, but just averaged across responses. The final learned values are then used to simulate test phase choices.

For each test trial, we then used the learned values of the two stimulus options (presented on left and right for each test trial) and predicted the probability of choosing the higher-valued option based on value-differences (Eq. 10; value difference policy $\pi_{\Delta V}=[p_{s_{L}},p_{s_{R}}]$). The inverse temperature was fixed at β=100 for both models, identical to its treatment in the learning phase, so that the comparison reflected only the learned values and the shared temperature. This probability was mixed with a participant-specific lapse process estimated using undirected noise parameter ϵ from the train phase. The resulting mixture policy gives the final decision policy for the test phase (Eq. 11).


$$\begin{array}{c} \pi_{\Delta V}(s_{a})=\frac{\exp(\beta \cdot V(s_{a}))}{\sum_{i\in \{L,R\}}\exp(\beta \cdot V(s_{i}))} \end{array}$$ (10)

$$\begin{array}{c} \pi_{test}=(1-\epsilon )\cdot \pi_{\Delta V}+\epsilon \cdot (\frac{1}{2}) \end{array}$$ (11)

We binned test trials into within-participant absolute value difference tertiles (low, medium, and high) and compared model-predicted to empirical accuracy in each bin. To derive the psychometric curve, the signed differences were divided into seven equal-frequency bins across trials.

For the test phase decision rule we used a softmax choice function (Eq. 10) combined with the fitted undirected-noise parameter ϵ (Eq. 11), applied identically to both the PADDL and choice-only models. We used softmax rather than the LBA decision process employed during learning for two reasons. First, the analysis concerns whether the learned values predict out-of-sample choice, so the decision rule should be a minimal, assumption-light mapping from value to choice that is common to both models; holding the rule fixed across models ensures that any difference in predicted accuracy is attributable to the learned values rather than to the decision process. Second, it would not be sensible to use the learning phase LBA in the test phase, given that the former required selecting among three instrumental responses for a single stimulus, and the latter required assessing which of two stimuli was more rewarded. Accordingly, the train and test response time distributions differ substantially in their summary statistics (minimum, median, and maximum). (Additionally, the learning phase decision threshold was parameterized as an explicit function of set size (a=m·ns+k) for which the test phase has no analog, and the start-point bias would have to be assumed invariant across phases despite the distributional shift.) Because the analysis targets choice given learned value rather than within-trial decision dynamics, we did not model test phase response times.

We evaluated probabilistic calibration on held-out test choices using logistic regression. For each model, observed right choice was regressed on the logit of its posterior-predictive right-choice probability; ideal calibration corresponds to intercept 0 and slope 1. To compare models directly, trial outcomes were stacked once per model and regressed on predicted log odds, model indicator, and their interaction. The interaction estimated the choice-only-minus-PADDL calibration-slope difference. Sandwich standard errors were clustered by participant, accounting for repeated trials and the paired predictions for each outcome.

### Parameter recovery

To validate our modeling approach, we conducted a parameter recovery analysis. We generated a synthetic dataset for 87 simulated participants using the mean of all participant-level parameter posteriors obtained from fitting the empirical data. We then fit both the joint PADDL model and the choice-only model to this synthetic dataset using the same hierarchical structure and priors as in the model fitting step to assess whether the true data-generating parameters could be accurately recovered.

To assess recovery accuracy, we calculated Spearman’s rank correlation between the true data-generating parameters and the recovered estimates for each model. For statistical comparison, each correlation was converted to a Pearson-equivalent value via the sine transform. These values were then Fisher z-transformed. To formally compare the recovery performance of the joint and choice-only models, we contrasted their resulting *z*-values to obtain the *p*-values.

### Clinical group contrasts

We compared the group-level parameter distributions estimated from the model fit to link behavioral differences in clinical groups to the parameters of the generative cognitive process. To assess the significance of these differences, we estimate the 94% HDI of the contrast $\left\{\delta^{1},\delta^{2},...,\delta^{N}\right\}$ where $\delta^{i}=\vartheta_{HC}^{i}-\vartheta_{group}^{i}$, $\vartheta^{i}\sim p(\vartheta |data)$ and *N* is the number of posterior samples. If zero is contained in the contrast HDI range, we conclude that there is no credible difference between the two groups.

### Summary statistics

In our modeling results (see Fig 3B-3E), we examine two critical characteristics of the PADDL model – the proactive control effect and RL effect. The effects account for the subtle but crucial behavioral phenomena (in RT distributions) during early- and late-stage learning. The proactive control effect (characterized by parameter *m* – via proactively adjusted decision threshold as a function of WM load or set size) describes the model’s ability to capture the RTs during the initial stimulus iterations whereas the RL effect (characterized by parameter α – via the contribution of slow, incremental learning in the RL system) describes the ability to capture the late-stage RTs. To assess these effects, we plot the difference in mean RTs across set sizes for across-model comparison (e.g., ablation studies on PADDL vs. PADDL variants) and across-participant comparison (e.g., contrasting high- and low-parameter subgroups within HC). For proactive control effect, the difference in the mean RTs is plotted for stimulus iterations 2 and 3. For the RL effect, the difference in the mean RTs is plotted for the last two stimulus iterations (9 and 10). The mean and HDI of the group-averaged measures are computed across the simulation runs to denote the confidence in estimating the group-level means.

Similarly, to characterize the differences in RT slopes across set sizes in the HC and SCZ groups (see Fig 5C), we plot the slope (or change in RT) over the course of learning as a summary statistic. We compute the difference in RTs between stimulus iterations 1–2 and 9–10. The mean and HDI of the group-averaged measures are computed across the simulation runs to denote the confidence in estimating the group-level means.

## Supporting information

S1 Text Supplementary material. Single supporting file containing Figs A-I and Tables A-C, labeled as follows. **Fig A**, model predictions using posterior predictive checks for the PADDL and choice-only softmax models (HC group). **Fig B**, model predictions using posterior predictive checks for the PADDL model without the RL contribution (HC group). **Fig C**, out-of-sample validation of estimated parameters on the held-out test phase (SCZ group). **Fig D**, posterior predictive checks for the BP and MDD groups. **Fig E**, group-level posterior distributions across all clinical groups. **Fig F**, parameter recovery for the joint (PADDL) and choice-only (softmax) models. **Fig G**, comparison of empirical and simulated RT distributions. **Fig H**, VI loss as a function of iterations for multiple seeds, with convergence checks. **Fig I**, posterior pair plots for the group-level parameters. **Table A**, summary of posterior parameter estimates across all groups. **Table B**, summary of contrasts across all groups. **Table C**, demographic information about the groups.
(PDF)

## References

[1] Sutton RS, Barto AG. Reinforcement learning: an introduction. 2nd ed. Cambridge, Massachusetts: The MIT Press. 2018.

[2] Radulescu A, Niv Y, Ballard I. Holistic reinforcement learning: the role of structure and attention. Trends Cogn Sci. 2019;23(4):278–92. doi: 10.1016/j.tics.2019.01.010 30824227 PMC6472955

[3] Seymour B, O’Doherty JP, Dayan P, Koltzenburg M, Jones AK, Dolan RJ, et al. Temporal difference models describe higher-order learning in humans. Nature. 2004;429(6992):664–7. doi: 10.1038/nature02581 15190354

[4] Botvinick MM, Niv Y, Barto AG. Hierarchically organized behavior and its neural foundations: a reinforcement learning perspective. Cognition. 2009;113(3):262–80. doi: 10.1016/j.cognition.2008.08.011 18926527 PMC2783353

[5] Montague PR, Dayan P, Sejnowski TJ. A framework for mesencephalic dopamine systems based on predictive Hebbian learning. J Neurosci. 1996;16(5):1936–47. doi: 10.1523/JNEUROSCI.16-05-01936.1996 8774460 PMC6578666

[6] Schultz W, Dayan P, Montague PR. A neural substrate of prediction and reward. Science. 1997;275(5306):1593–9. doi: 10.1126/science.275.5306.1593 9054347

[7] Frank MJ. Adaptive cost-benefit control fueled by striatal dopamine. Annu Rev Neurosci. 2025;48(1):1–22. doi: 10.1146/annurev-neuro-112723-025228 39813735

[8] Collins AGE, Frank MJ. Opponent actor learning (OpAL): modeling interactive effects of striatal dopamine on reinforcement learning and choice incentive. Psychol Rev. 2014;121(3):337–66. doi: 10.1037/a0037015 25090423

[9] Jaskir A, Frank MJ. On the normative advantages of dopamine and striatal opponency for learning and choice. Elife. 2023;12:e85107. doi: 10.7554/eLife.85107 36946371 PMC10198727

[10] O’Reilly RC, Frank MJ. Making working memory work: a computational model of learning in the prefrontal cortex and basal ganglia. Neural Comput. 2006;18(2):283–328. doi: 10.1162/089976606775093909 16378516

[11] Rmus M, McDougle SD, Collins AGE. The role of executive function in shaping reinforcement learning. Curr Opin Behav Sci. 2021;38:66–73. doi: 10.1016/j.cobeha.2020.10.003 35194556 PMC8859995

[12] Collins AGE, Frank MJ. How much of reinforcement learning is working memory, not reinforcement learning? A behavioral, computational, and neurogenetic analysis. Eur J Neurosci. 2012;35(7):1024–35. doi: 10.1111/j.1460-9568.2011.07980.x 22487033 PMC3390186

[13] Niv Y, Daniel R, Geana A, Gershman SJ, Leong YC, Radulescu A, et al. Reinforcement learning in multidimensional environments relies on attention mechanisms. J Neurosci. 2015;35(21):8145–57. doi: 10.1523/JNEUROSCI.2978-14.2015 26019331 PMC4444538

[14] Frank MJ, Claus ED. Anatomy of a decision: striato-orbitofrontal interactions in reinforcement learning, decision making, and reversal. Psychol Rev. 2006;113(2):300–26. doi: 10.1037/0033-295X.113.2.300 16637763

[15] Frank MJ, Moustafa AA, Haughey HM, Curran T, Hutchison KE. Genetic triple dissociation reveals multiple roles for dopamine in reinforcement learning. Proc Natl Acad Sci U S A. 2007;104(41):16311–6. doi: 10.1073/pnas.0706111104 17913879 PMC2042203

[16] Hitchcock PF, Kim J, Frank MJ. How working memory and reinforcement learning interact when avoiding punishment and pursuing reward concurrently. J Exp Psychol Gen. 2025;154(11):2990–3009. doi: 10.1037/xge0001817 40892600 PMC12407242

[17] Rac-Lubashevsky R, Cremer A, Collins AGE, Frank MJ, Schwabe L. Neural index of reinforcement learning predicts improved stimulus-response retention under high working memory load. J Neurosci. 2023;43(17):3131–43. doi: 10.1523/JNEUROSCI.1274-22.2023 36931706 PMC10146488

[18] McDougle SD, Collins AGE. Modeling the influence of working memory, reinforcement, and action uncertainty on reaction time and choice during instrumental learning. Psychon Bull Rev. 2021;28(1):20–39. doi: 10.3758/s13423-020-01774-z 32710256 PMC7854965

[19] Collins AGE, Brown JK, Gold JM, Waltz JA, Frank MJ. Working memory contributions to reinforcement learning impairments in schizophrenia. J Neurosci. 2014;34(41):13747–56. doi: 10.1523/JNEUROSCI.0989-14.2014 25297101 PMC4188972

[20] Rmus M, He M, Baribault B, Walsh EG, Festa EK, Collins AGE, et al. Age-related differences in prefrontal glutamate are associated with increased working memory decay that gives the appearance of learning deficits. Elife. 2023;12:e85243. doi: 10.7554/eLife.85243 PMC10174689 37070807

[21] Eckstein MK, Wilbrecht L, Collins AGE. What do reinforcement learning models measure? Interpreting model parameters in cognition and neuroscience. Curr Opin Behav Sci. 2021;41:128–37. doi: 10.1016/j.cobeha.2021.06.004 34984213 PMC8722372

[22] Yoo AH, Collins AGE. How working memory and reinforcement learning are intertwined: a cognitive, neural, and computational perspective. J Cogn Neurosci. 2022;34(4):551–68. doi: 10.1162/jocn_a_01808 34942642

[23] Collins AGE, Albrecht MA, Waltz JA, Gold JM, Frank MJ. Interactions among working memory, reinforcement learning, and effort in value-based choice: a new paradigm and selective deficits in schizophrenia. Biol Psychiatry. 2017;82(6):431–9. doi: 10.1016/j.biopsych.2017.05.017 PMC5573149 28651789

[24] Hick WE. On the rate of gain of information. Q J Exp Psychol. 1952;4(1):11–26. doi: 10.1080/17470215208416600

[25] Peters J, D’Esposito M. The drift diffusion model as the choice rule in inter-temporal and risky choice: a case study in medial orbitofrontal cortex lesion patients and controls. PLoS Comput Biol. 2020;16(4):e1007615. doi: 10.1371/journal.pcbi.1007615 PMC7192518 32310962

[26] Pedersen ML, Frank MJ, Biele G. The drift diffusion model as the choice rule in reinforcement learning. Psychon Bull Rev. 2017;24(4):1234–51. doi: 10.3758/s13423-016-1199-y 27966103 PMC5487295

[27] Frank MJ, Gagne C, Nyhus E, Masters S, Wiecki TV, Cavanagh JF, et al. fMRI and EEG predictors of dynamic decision parameters during human reinforcement learning. J Neurosci. 2015;35(2):485–94. doi: 10.1523/JNEUROSCI.2036-14.2015 25589744 PMC4293405

[28] Lai L, Gershman SJ. Chapter five - policy compression: an information bottleneck in action selection. In: Federmeier KD, editor. Psychology of Learning and Motivation. Academic Press. 2021. p. 195–232.

[29] Fontanesi L, Palminteri S, Lebreton M. Decomposing the effects of context valence and feedback information on speed and accuracy during reinforcement learning: a meta-analytical approach using diffusion decision modeling. Cogn Affect Behav Neurosci. 2019;19(3):490–502. doi: 10.3758/s13415-019-00723-1 31175616 PMC6598978

[30] Fontanesi L, Gluth S, Spektor MS, Rieskamp J. A reinforcement learning diffusion decision model for value-based decisions. Psychon Bull Rev. 2019;26(4):1099–121. doi: 10.3758/s13423-018-1554-2 30924057 PMC6820465

[31] Miletić S, Boag RJ, Forstmann BU. Mutual benefits: Combining reinforcement learning with sequential sampling models. Neuropsychologia. 2020;136:107261. doi: 10.1016/j.neuropsychologia.2019.107261 31733237

[32] Bera K. An empirical and computational investigation of skill learning in internally-guided sequencing. Master’s thesis, IIIT/TH/2021/56. India: International Institute of Information Technology Hyderabad. 2021.

[33] Miletić S, Boag RJ, Trutti AC, Stevenson N, Forstmann BU, Heathcote A. A new model of decision processing in instrumental learning tasks. Elife. 2021;10:e63055. doi: 10.7554/eLife.63055 33501916 PMC7880686

[34] Luzardo A, Alonso E, Mondragón E. A Rescorla-Wagner drift-diffusion model of conditioning and timing. PLoS Comput Biol. 2017;13(11):e1005796. doi: 10.1371/journal.pcbi.1005796 PMC5685643 29095819

[35] Sewell DK, Jach HK, Boag RJ, Van Heer CA. Combining error-driven models of associative learning with evidence accumulation models of decision-making. Psychon Bull Rev. 2019;26(3):868–93. doi: 10.3758/s13423-019-01570-4 30719625

[36] Clithero JA. Improving out-of-sample predictions using response times and a model of the decision process. J Econ Behav Organ. 2018;148:344–75. doi: 10.1016/j.jebo.2018.02.007

[37] Fengler A, Govindarajan LN, Chen T, Frank MJ. Likelihood approximation networks (LANs) for fast inference of simulation models in cognitive neuroscience. Elife. 2021;10:e65074. doi: 10.7554/eLife.65074 33821788 PMC8102064

[38] Fengler A, Bera K, Pedersen ML, Frank MJ. Beyond drift diffusion models: fitting a broad class of decision and reinforcement learning models with HDDM. J Cogn Neurosci. 2022;34(10):1780–805. doi: 10.1162/jocn_a_01902 35939629

[39] Rmus M, Pan T-F, Xia L, Collins AGE. Artificial neural networks for model identification and parameter estimation in computational cognitive models. PLoS Comput Biol. 2024;20(5):e1012119. doi: 10.1371/journal.pcbi.1012119 38748770 PMC11132492

[40] Fengler A, Xu Y, Bera K, Paniagua C, Omar A, Frank MJ. HSSM: A widely applicable toolbox for hierarchical Bayesian neurocognitive modeling. Elife. 2026;15:RP112168. doi: 10.7554/elife.112168.1

[41] Brown SD, Heathcote A. The simplest complete model of choice response time: linear ballistic accumulation. Cogn Psychol. 2008;57(3):153–78. doi: 10.1016/j.cogpsych.2007.12.002 18243170

[42] Westbrook A, van den Bosch R, Hofmans L, Papadopetraki D, Määttä JI, Collins AGE, et al. Striatal dopamine can enhance both fast working memory, and slow reinforcement learning, while reducing implicit effort cost sensitivity. Nat Commun. 2025;16(1):6320. doi: 10.1038/s41467-025-61099-0 40634287 PMC12241355

[43] Collins AGE, Frank MJ. Within- and across-trial dynamics of human EEG reveal cooperative interplay between reinforcement learning and working memory. Proc Natl Acad Sci U S A. 2018;115(10):2502–7. doi: 10.1073/pnas.1720963115 29463751 PMC5877949

[44] Ging-Jehli NR, Rac-Lubashevsky R, Bera K, Boudewyn MA, Carter CS, Erickson MA, et al. Model-based electroencephalography phenotyping uncovers distinct neurocomputational mechanisms underlying learning impairments across psychopathologies. Biological Psychiatry Global Open Science. 2026;6(2):100660. doi: 10.1016/j.bpsgos.2025.100660 PMC12876727 41659278

[45] Ballard IC, McClure SM. Joint modeling of reaction times and choice improves parameter identifiability in reinforcement learning models. J Neurosci Methods. 2019;317:37–44. doi: 10.1016/j.jneumeth.2019.01.006 30664916 PMC8930195

[46] Ratcliff R, McKoon G. The diffusion decision model: theory and data for two-choice decision tasks. Neural Comput. 2008;20(4):873–922. doi: 10.1162/neco.2008.12-06-420 18085991 PMC2474742

[47] Cavanagh JF, Wiecki TV, Cohen MX, Figueroa CM, Samanta J, Sherman SJ, et al. Subthalamic nucleus stimulation reverses mediofrontal influence over decision threshold. Nat Neurosci. 2011;14(11):1462–7. doi: 10.1038/nn.2925 21946325 PMC3394226

[48] Ging-Jehli NR, Cavanagh JF, Ahn M, Segar DJ, Asaad WF, Frank MJ. Basal ganglia components have distinct computational roles in decision-making dynamics under conflict and uncertainty. PLoS Biol. 2025;23(1):e3002978. doi: 10.1371/journal.pbio.3002978 39847590 PMC11756759

[49] Braver TS. The variable nature of cognitive control: a dual mechanisms framework. Trends Cogn Sci. 2012;16(2):106–13. doi: 10.1016/j.tics.2011.12.010 22245618 PMC3289517

[50] Frank MJ, Seeberger LC, O’Reilly RC. By carrot or by stick: cognitive reinforcement learning in parkinsonism. Science. 2004;306(5703):1940–3. doi: 10.1126/science.1102941 15528409

[51] Lesh TA, Niendam TA, Minzenberg MJ, Carter CS. Cognitive control deficits in schizophrenia: mechanisms and meaning. Neuropsychopharmacology. 2011;36(1):316–38. doi: 10.1038/npp.2010.156 20844478 PMC3052853

[52] van Veen V, Carter CS. The anterior cingulate as a conflict monitor: fMRI and ERP studies. Physiol Behav. 2002;77(4–5):477–82. doi: 10.1016/s0031-9384(02)00930-7 12526986

[53] Shahar N, Hauser TU, Moutoussis M, Moran R, Keramati M, NSPN consortium, et al. Improving the reliability of model-based decision-making estimates in the two-stage decision task with reaction-times and drift-diffusion modeling. PLoS Comput Biol. 2019;15(2):e1006803. doi: 10.1371/journal.pcbi.1006803 30759077 PMC6391008

[54] Gelman A, Carlin JB, Stern HS, Dunson DB, Vehtari A, Rubin DB. Bayesian data analysis. 3rd ed. New York, NY, US: Chapman and Hall/CRC. 2014.

[55] Schmitz F, Voss A. Decomposing task-switching costs with the diffusion model. J Exp Psychol Hum Percept Perform. 2012;38(1):222–50. doi: 10.1037/a0026003 22060144

[56] Cohen Hoffing R, Karvelis P, Rupprechter S, Seriès P, Seitz AR. The influence of feedback on task-switching performance: a drift diffusion modeling account. Front Integr Neurosci. 2018;12:1. doi: 10.3389/fnint.2018.00001 29456494 PMC5801306

[57] Boag RJ, Strickland L, Heathcote A, Neal A, Loft S. Cognitive control and capacity for prospective memory in complex dynamic environments. J Exp Psychol Gen. 2019;148(12):2181–206. doi: 10.1037/xge0000599 31008627

[58] Anderson TF, Rummel J, McDaniel MA. Proceeding with care for successful prospective memory: do we delay ongoing responding or actively monitor for cues?. J Exp Psychol: Learn Mem Cogn. 2018;44(7):1036–50. doi: 10.1037/xlm0000504 29553768

[59] Strickland L, Loft S, Remington RW, Heathcote A. Racing to remember: a theory of decision control in event-based prospective memory. Psychol Rev. 2018;125(6):851–87. doi: 10.1037/rev0000113 30080068

[60] Corlazzoli G, Gevers W, Notebaert W, Desender K. Brace yourself: neural and computational insights into the experience of mental effort. Cereb Cortex. 2025;35(10):bhaf256. doi: 10.1093/cercor/bhaf256 41110134

[61] Barch DM, Sheffield JM. Cognitive control in Schizophrenia. The Wiley Handbook of Cognitive Control. Wiley. 2017. 556–80. doi: 10.1002/9781118920497.ch31

[62] Lesh TA, Westphal AJ, Niendam TA, Yoon JH, Minzenberg MJ, Ragland JD, et al. Proactive and reactive cognitive control and dorsolateral prefrontal cortex dysfunction in first episode schizophrenia. Neuroimage Clin. 2013;2:590–9. doi: 10.1016/j.nicl.2013.04.010 24179809 PMC3777717

[63] Maia TV, Frank MJ. An integrative perspective on the role of dopamine in Schizophrenia. Biol Psychiatry. 2017;81(1):52–66. doi: 10.1016/j.biopsych.2016.05.021 27452791 PMC5486232

[64] Strauss GP, Frank MJ, Waltz JA, Kasanova Z, Herbener ES, Gold JM. Deficits in positive reinforcement learning and uncertainty-driven exploration are associated with distinct aspects of negative symptoms in schizophrenia. Biol Psychiatry. 2011;69(5):424–31. doi: 10.1016/j.biopsych.2010.10.015 21168124 PMC3039035

[65] Papamakarios G, Sterratt D, Murray I. Sequential neural likelihood: fast likelihood-free inference with autoregressive flows. In: Proceedings of the Twenty-Second International Conference on Artificial Intelligence and Statistics, 2019. 837–48.

[66] Lueckmann J-M, Bassetto G, Karaletsos T, Macke J. Likelihood-free inference with emulator networks. ArXiv. 2018. doi: 10.48550/arXiv.1805.09294

[67] Radev ST, Mertens UK, Voss A, Köthe U. Towards end-to-end likelihood-free inference with convolutional neural networks. Br J Math Stat Psychol. 2020;73(1):23–43. doi: 10.1111/bmsp.12159 30793299

[68] Tillman G, Van Zandt T, Logan GD. Sequential sampling models without random between-trial variability: the racing diffusion model of speeded decision making. Psychon Bull Rev. 2020;27(5):911–36. doi: 10.3758/s13423-020-01719-6 32424622

[69] Usher M, McClelland JL. The time course of perceptual choice: the leaky, competing accumulator model. Psychol Rev. 2001;108(3):550–92. doi: 10.1037/0033-295x.108.3.550 11488378

[70] Smith PL. “Reliable organisms from unreliable components” revisited: the linear drift, linear infinitesimal variance model of decision making. Psychon Bull Rev. 2023;30(4):1323–59. doi: 10.3758/s13423-022-02237-3 36720804 PMC10482797

[71] Wagenmakers E-J, Lodewyckx T, Kuriyal H, Grasman R. Bayesian hypothesis testing for psychologists: a tutorial on the Savage-Dickey method. Cogn Psychol. 2010;60(3):158–89. doi: 10.1016/j.cogpsych.2009.12.001 20064637

[72] Blei DM, Kucukelbir A, McAuliffe JD. Variational inference: a review for statisticians. J Am Stat Assoc. 2017;112(518):859–77. doi: 10.1080/01621459.2017.1285773

[73] Kingma DP, Welling M. Auto-encoding variational Bayes. arXiv. 2013. doi: 10.48550/ARXIV.1312.6114

[74] Kucukelbir A, Tran D, Ranganath R, Gelman A, Blei DM. Automatic differentiation variational inference. J Mach Learn Res. 2017;18(14):1–45.

[75] Abril-Pla O, Andreani V, Carroll C, Dong L, Fonnesbeck CJ, Kochurov M, et al. PyMC: a modern, and comprehensive probabilistic programming framework in Python. PeerJ Comput Sci. 2023;9:e1516. doi: 10.7717/peerj-cs.1516 PMC10495961 37705656

[76] Bradbury J, Frostig R, Hawkins P, Johnson MJ, Leary C, Maclaurin D, et al. JAX: composable transformations of Python NumPy programs. http://github.com/jax-ml/jax 2018.

[77] PyTensor Developers. Pytensor. 2024. URL https://github.com/pymc-devs/pytensor

[78] Fengler A, Paniagua C, Bera K, Zhang A, Omar A, Hafner H, et al. lnccbrown/ssm-simulators: 0.14.0 (Version v0.14.0) [Computer software]. Zenodo. 2026. doi: 10.5281/zenodo.22459634

[79] Paszke A, Gross S, Massa F, Lerer A, Bradbury J, Chanan G, et al. Pytorch: an imperative style, high-performance deep learning library. Adv Neural Inf Process Syst. 2019;32.

[80] Steingroever H, Wetzels R, Wagenmakers E-J. Absolute performance of reinforcement-learning models for the Iowa Gambling Task. Decision. 2014;1(3):161–83. doi: 10.1037/dec0000005

