# Supplementary material for: Modeling decision dynamics disentangles working memory, cognitive control and reinforcement learning and reveals clinical differences
Source: PLoS Comput Biol. 2026 Sep 30;22(9):e1014796. doi: 10.1371/journal.pcbi.1014796 (PMC13660991; doi:10.1371/journal.pcbi.1014796)
Supplement: Supplementary file 1 [file pcbi.1014796.s001.pdf]

S1 Text. Supplementary material

Modeling decision dynamics disentangles working memory, cognitive control and reinforcement learning and reveals clinical differences

Krishn Bera, Alexander Fengler, Megan Boudewyn, Cameron Carter, Molly Erickson, James Gold, Steven Luck, J. Daniel Ragland, Andrew Yonelinas, Angus MacDonald III, Deanna Barch, Michael Frank

1 PADDL vs choice-only model: accuracy posterior predictive plots

We show the accuracy posterior predictive plots for the joint PADDL model and choice-only softmax model. Despite fixing the inverse temperature parameter $\beta = 100$ for both models, the choice-only fits remain flexible enough to capture the choice distributions, as is evident from comparing the PADDL and choice-only learning curves.

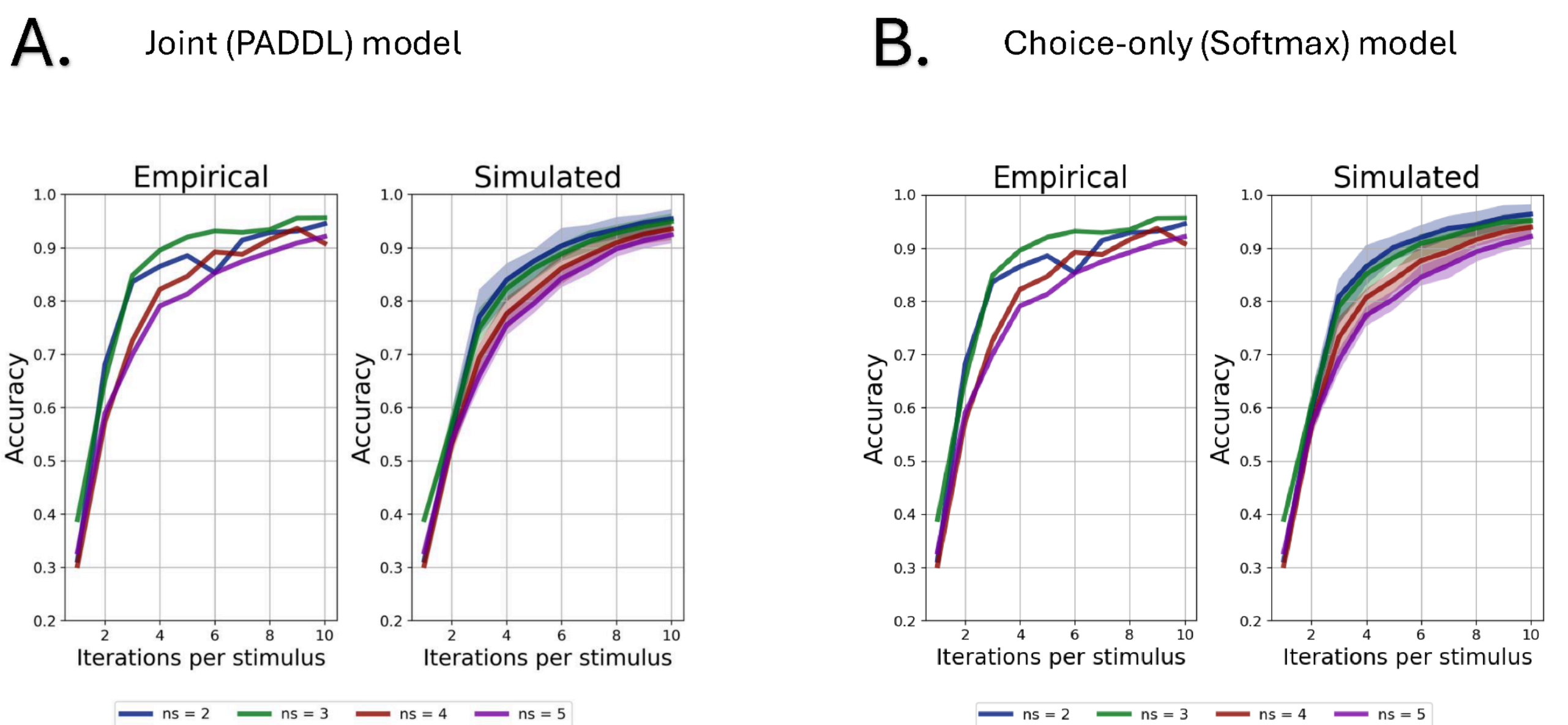

Fig A. Model predictions using posterior predictive checks for PADDL and choice-only softmax models (for HC group). Learning curves for each set size plotted in terms of accuracy for the model simulations. The model simulations were generated by sampling the posterior and using the post hoc absolute fit method. Shaded regions show 94% HDI ranges of the group mean.

2 Predictions from the PADDL model without RL contribution

Although the recovered learning rate α is small, it exerts a clear and measurable influence on behavior, which is what makes it identifiable. Because the softmax inverse temperature was fixed at $\beta = 100$, the effective change in the RL policy is appreciable (since the changes in Q values are magnified by 100), such that even a small learning rate produces a separation in learned values that is expressed in both the choice and RT distributions, and most visibly at higher set sizes and later trials, where the WM contribution is largely exhausted and the RL process dominates. The ablation experiments in Fig 3A, 3B and 3D make this concrete: overestimating α produces value separation that is too rapid and therefore predicts RTs that decline faster than observed, with the discrepancy growing at higher set sizes. To further demonstrate the RL contributions, we now show what happens when RL learning rates in PADDL are set to zero. With no RL contribution, the model fails to reproduce the empirical accuracy curves, with the largest discrepancy at higher set sizes, and predicts systematically slower RTs that depart from the observed trends. Together, these analyses show that the small but non-zero learning rate is necessary to account for the data and is well constrained by the joint choice-RT fit.

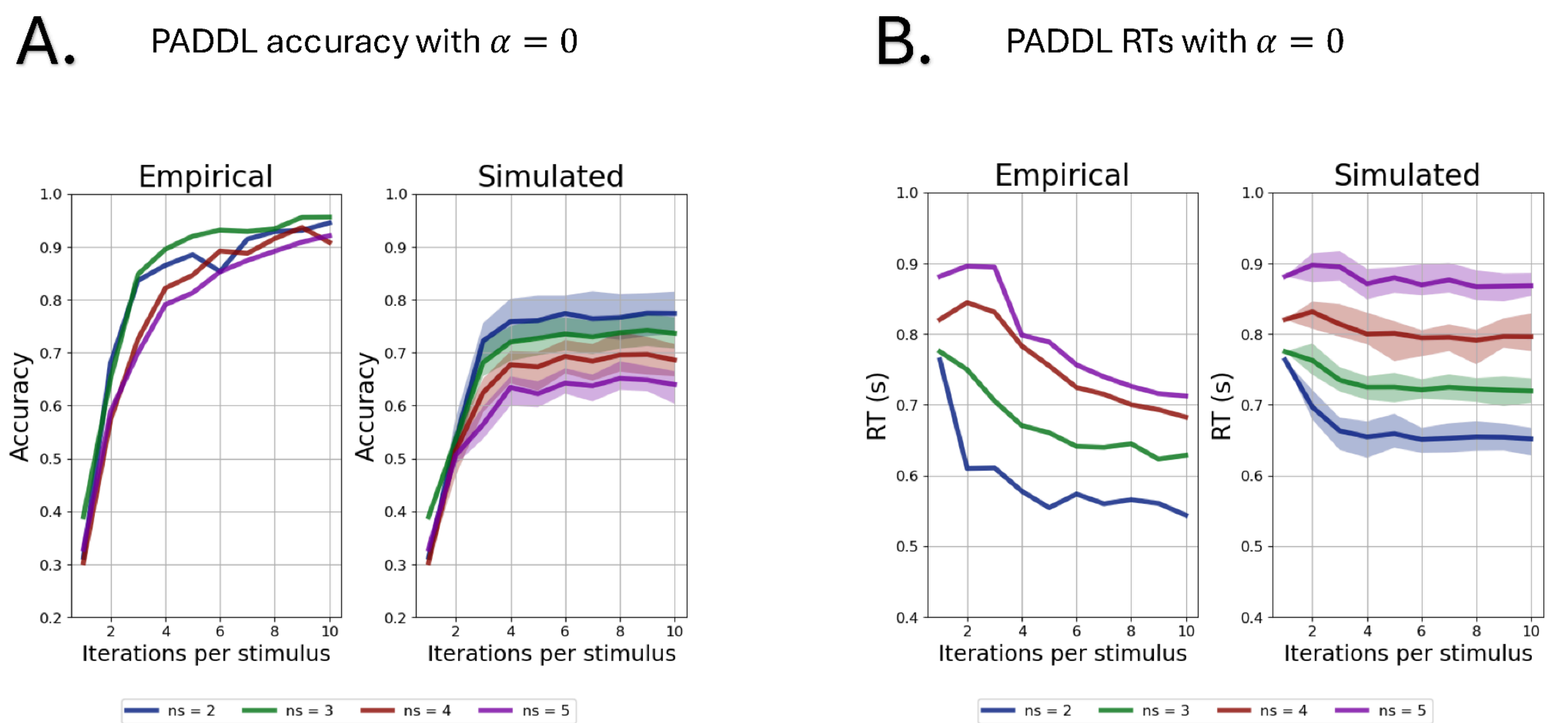

Fig B. Model predictions using posterior predictive checks for PADDL model without RL contribution (for HC group). Learning curves for each set size plotted in terms of accuracy and RT for the model simulations. The model simulations were generated by sampling the posterior and using the post hoc absolute fit method. Shaded regions show 94% HDI ranges of the group mean.

3 Test phase results for SCZ

Similar to the HC group, the PADDL-derived values tracked the empirical curve closely (Fig C), with only a slight underprediction at the largest value difference, substantially smaller than the softmax overprediction. Quantifying this as the signed deviation of predicted from empirical accuracy, the softmax model was substantially biased upward (mean deviation = 0.0670, CI [0.0480, 0.0873]) whereas PADDL was approximately unbiased (mean deviation = -0.0270, CI [-0.0465, -0.0060]). The PADDL model was favored in the participant-level log loss (mean difference = 0.0253 ± 0.0093 SEM). Note that this difference is significant in the case of SCZ, unlike HC.

To statistically test whether PADDL and the choice-only softmax model translate their internal predictions into empirically accurate choice probabilities during the test phase, we conducted a calibration analysis. This paired prediction analysis places both models in one fully interacted logistic calibration regression (see details in Methods). PADDL's calibration slope was 1.1558 (95% CI [0.8942, 1.4174]), consistent with ideal calibration (slope = 1). The choice-only softmax slope was 0.5394 (95% CI [0.4460, 0.6329]), indicating overconfident probabilities. The direct choice-only-minus-PADDL slope difference was -0.6164 (95% CI [-0.8012, -0.4316], cluster-robust $p = 6.27 \times 10^{-11}$). Calibration intercepts did not differ. PADDL's predicted choice probabilities closely matched participants' observed behavior, whereas the softmax model was systematically overconfident, assigning probabilities that were more extreme than the data supported. Thus, PADDL more accurately captured the strength of participants' choice preferences.

4 Clinical results for BP and MDD groups

We did not find other credible differences in model parameters for BP and MDD groups compared to the control group (see Table B). As in our prior study using choice-only RLWM models [1], BP showed increased WM decay (ϕ) compared to HC but the Bayesian analysis suggested a slightly greater degree of uncertainty in this result. The other key parameters such as the proactive adjustment of decision bounds and learning rate for the BP group were also estimated at intermediate values between the HC and SCZ groups but did not show a credible difference from either at the group level. The WM capacity for SCZ and BP groups was lower but not credibly different from the HC group. The MDD group did not show any credible contrast with the HC group (note this was also the case in our previous study, which required EEG biomarkers to dissociate MDD and BP; [1]). We note that these differences are in the group-level mean and with slightly overlapping HDI ranges as a consequence of Bayesian uncertainty

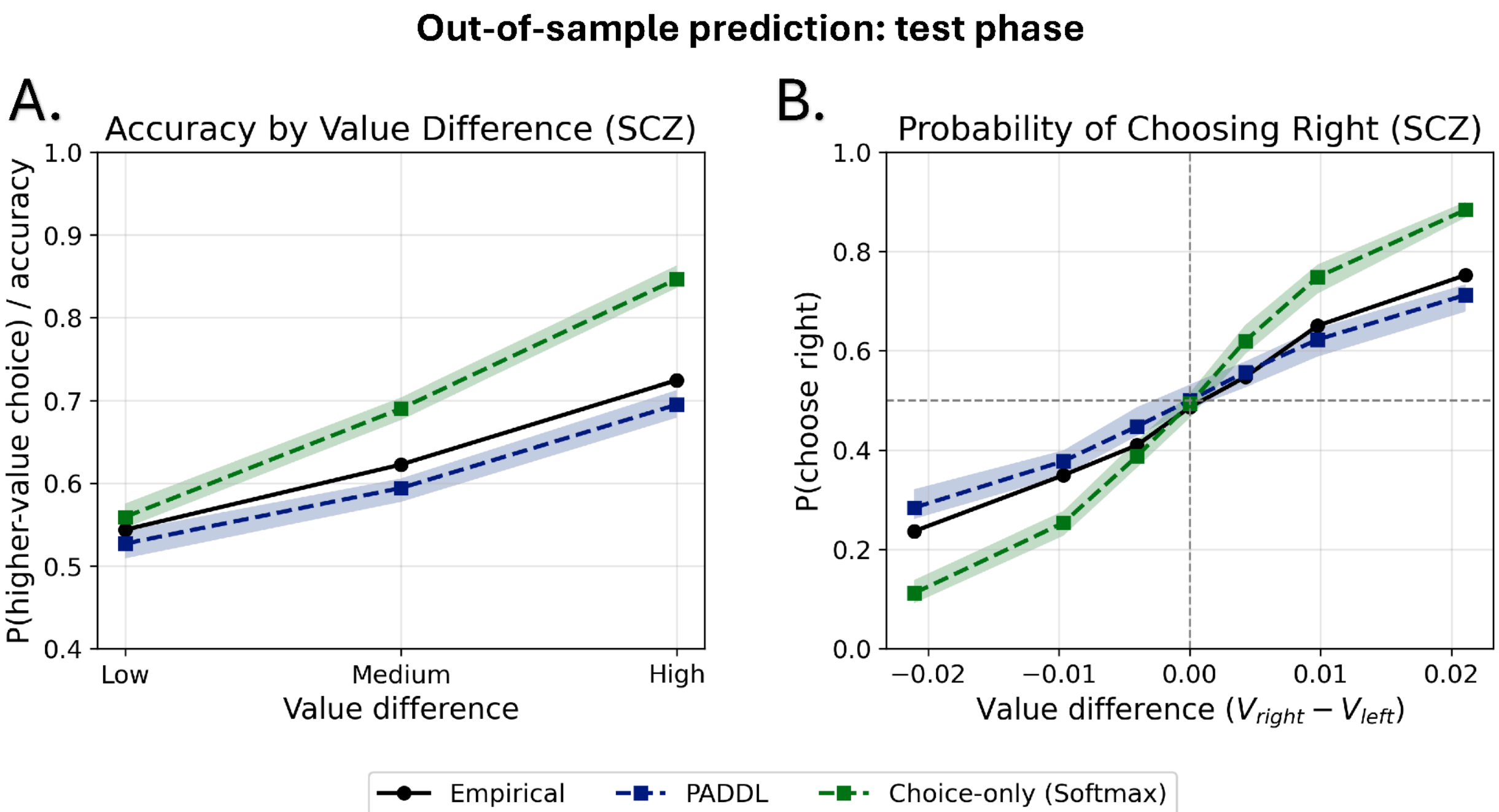

Fig C. Out-of-sample validation of estimated parameters on the held-out test phase (SCZ group). Test-phase choices were predicted from end-of-training RL values reconstructed from each participant's learning phase fit, with no parameters fitted to test data and an identical softmax rule applied to both models, so predictions differed only in the learned values each implied (see Methods). (A) Probability of choosing the higher-valued stimulus by within-participant value-difference tertile, for empirical data (black), PADDL (blue), and the choice-only softmax model (green). The choice-only model overpredicts accuracy, exceeding the empirical estimates at the medium and high bins, whereas PADDL tracks the empirical curve closely. (B) Psychometric function of choosing the right-hand stimulus against the signed value difference ($V_{\mathrm{right}} - V_{\mathrm{left}}$). The choice-only curve is too steep through indifference, the signature of over-separated learned values, while PADDL matches the empirical slope. Shaded bands denote 94% HDI.

quantification but the qualitative interpretation is unchanged. Future studies with larger sample sizes may be needed to evaluate whether there are individual differences or subgroups within each of these populations.

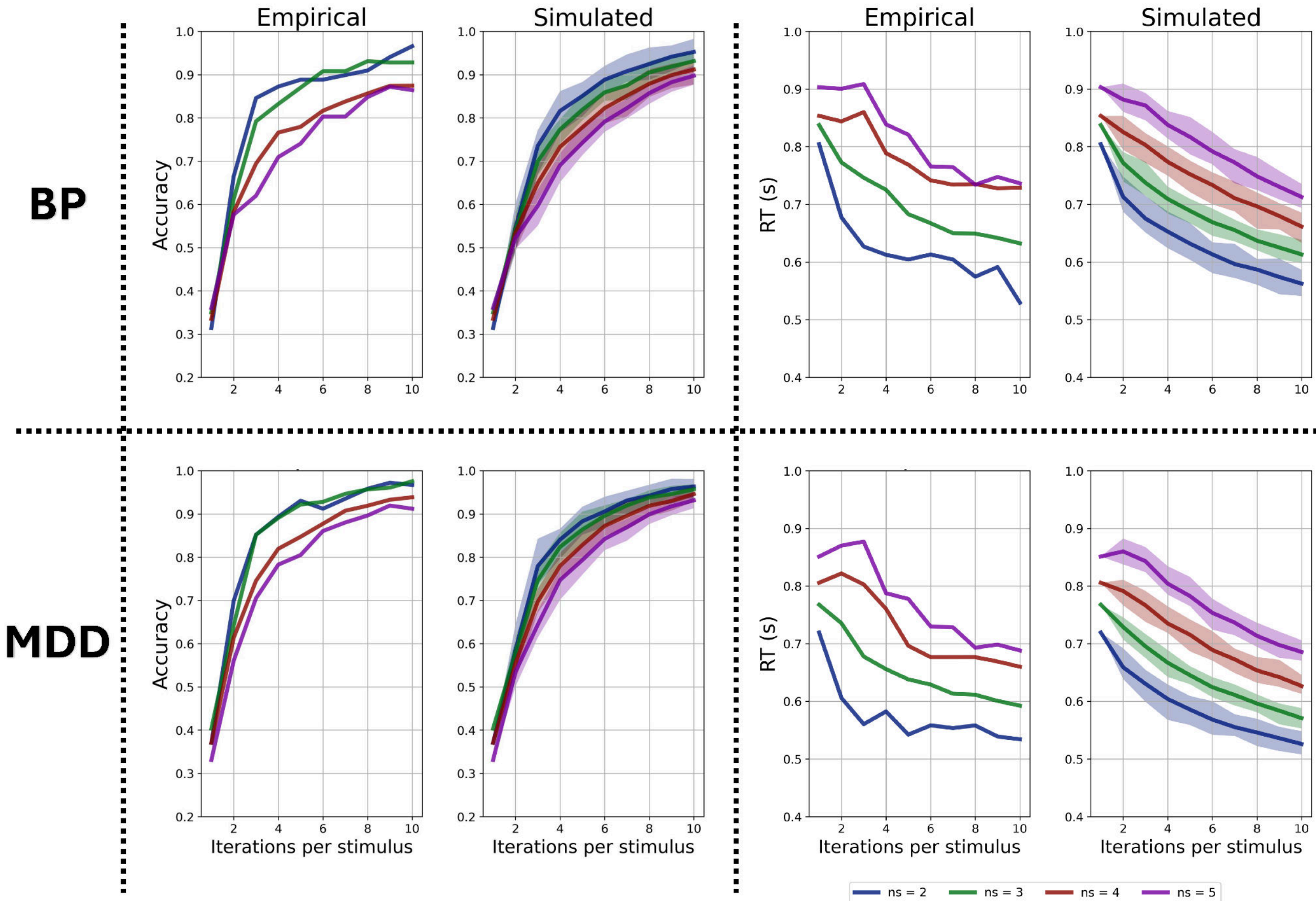

Fig D. Posterior predictive checks for BP and MDD. *(Left)* Learning curves for each set size plotted in terms of accuracy for empirical data and model simulations. The model simulations were generated by sampling the posterior and using the post hoc absolute fit method. *(Right)* Learning curves for each set size plotted in terms of RT trends for empirical data and model simulations. Shaded regions show 94% HDI ranges of the group mean.

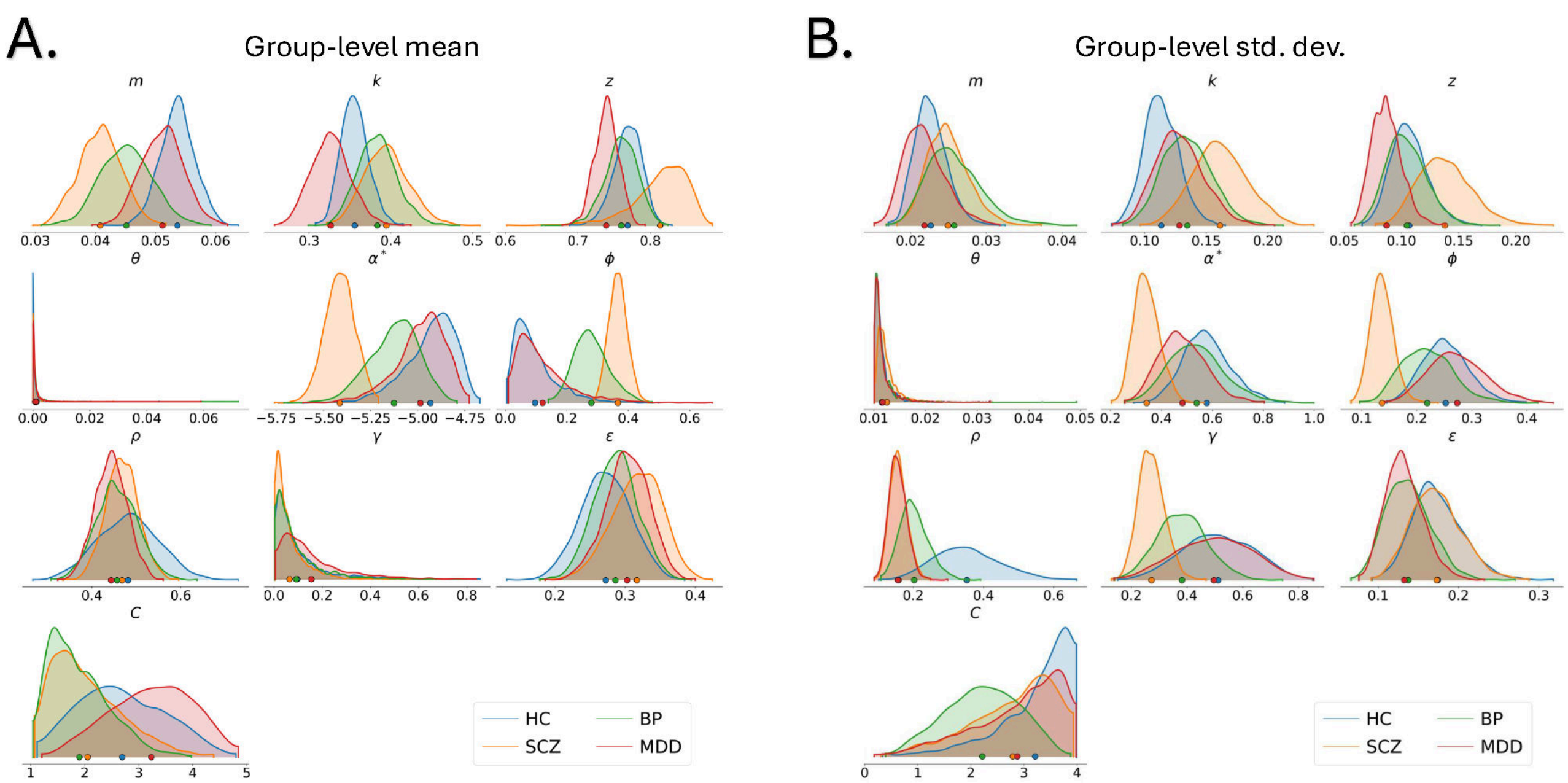

Fig E. Group-level posterior distributions across all clinical groups. (A) Posterior distributions of group-level means for each parameter. (B) Posterior distributions of group-level standard deviations for each parameter. Note that α^* denotes the log transformed parameter space of the learning rate α.

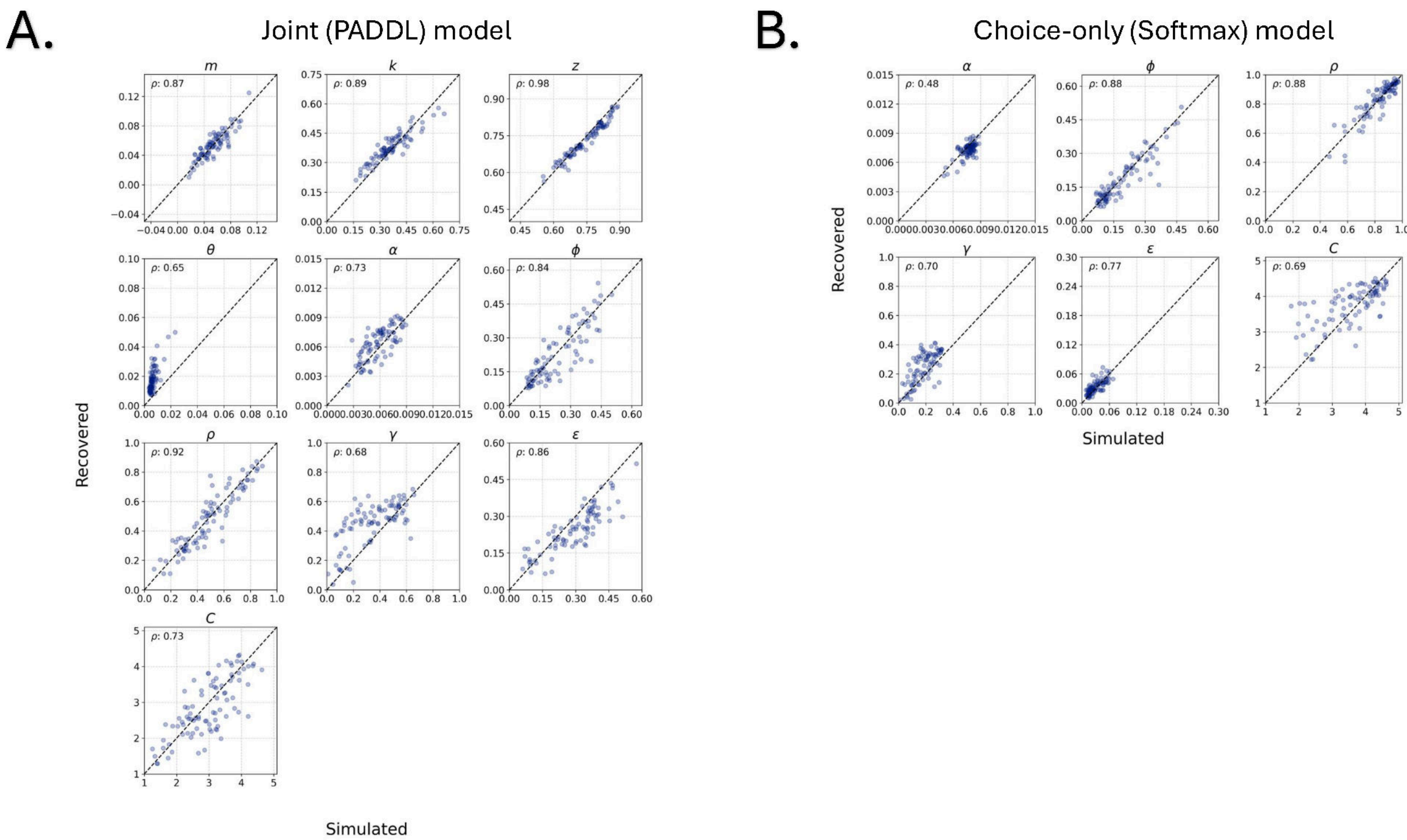

Fig F. Parameter recovery for the (A) joint (PADDL) model and (B) choice-only (softmax) model. Synthetic data were generated using participant-level parameter estimates obtained from fitting each model to the empirical HC data; recovery was then assessed by refitting the corresponding model to the simulated data.

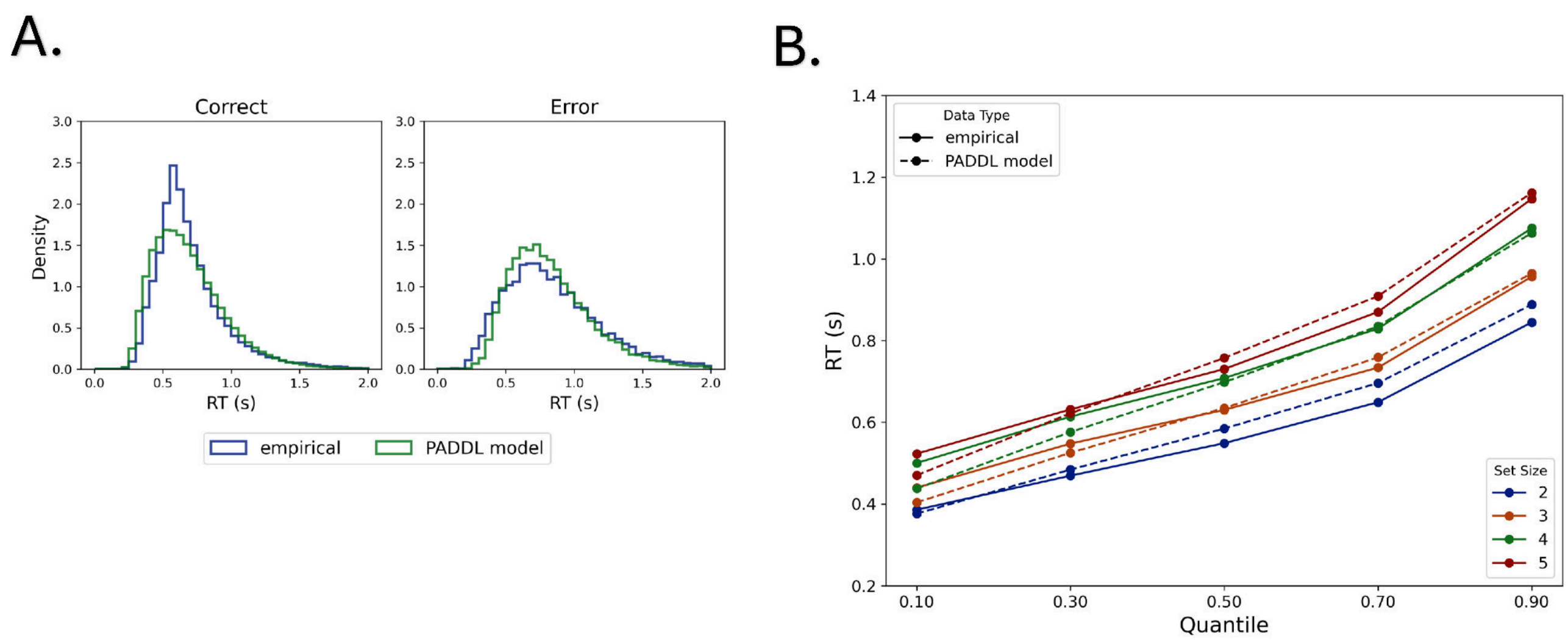

Fig G. Comparison of empirical and simulated RT distributions. (A) RT distributions for correct and error trials. (B) Quantile plots for the RT distributions.

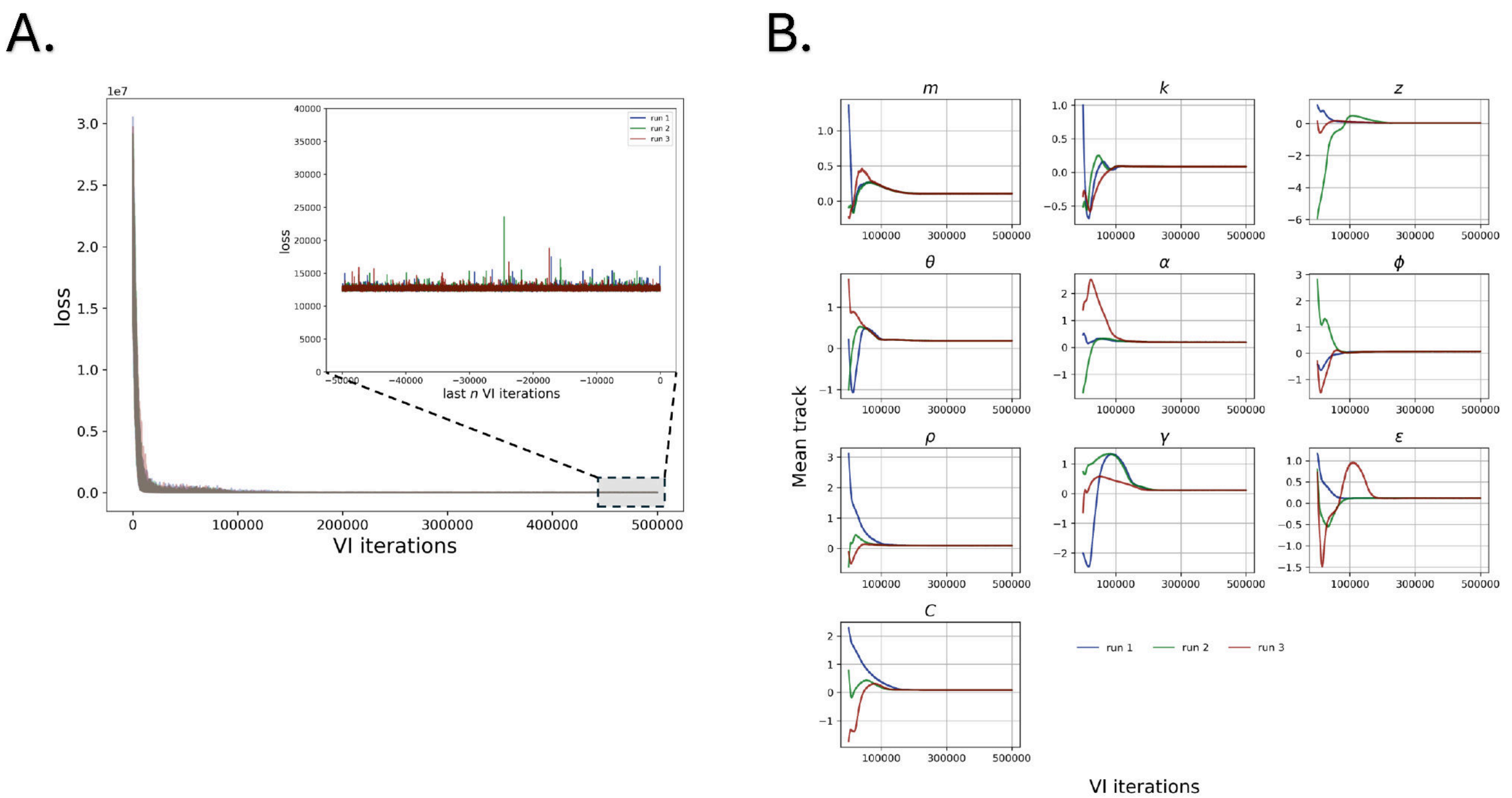

Fig H. (A) VI loss as a function of iterations for multiple seeds. Inset shows the stability of the loss for the last n iterations in the run. (B) VI track plotted for the group-level mean parameters for multiple seeds.

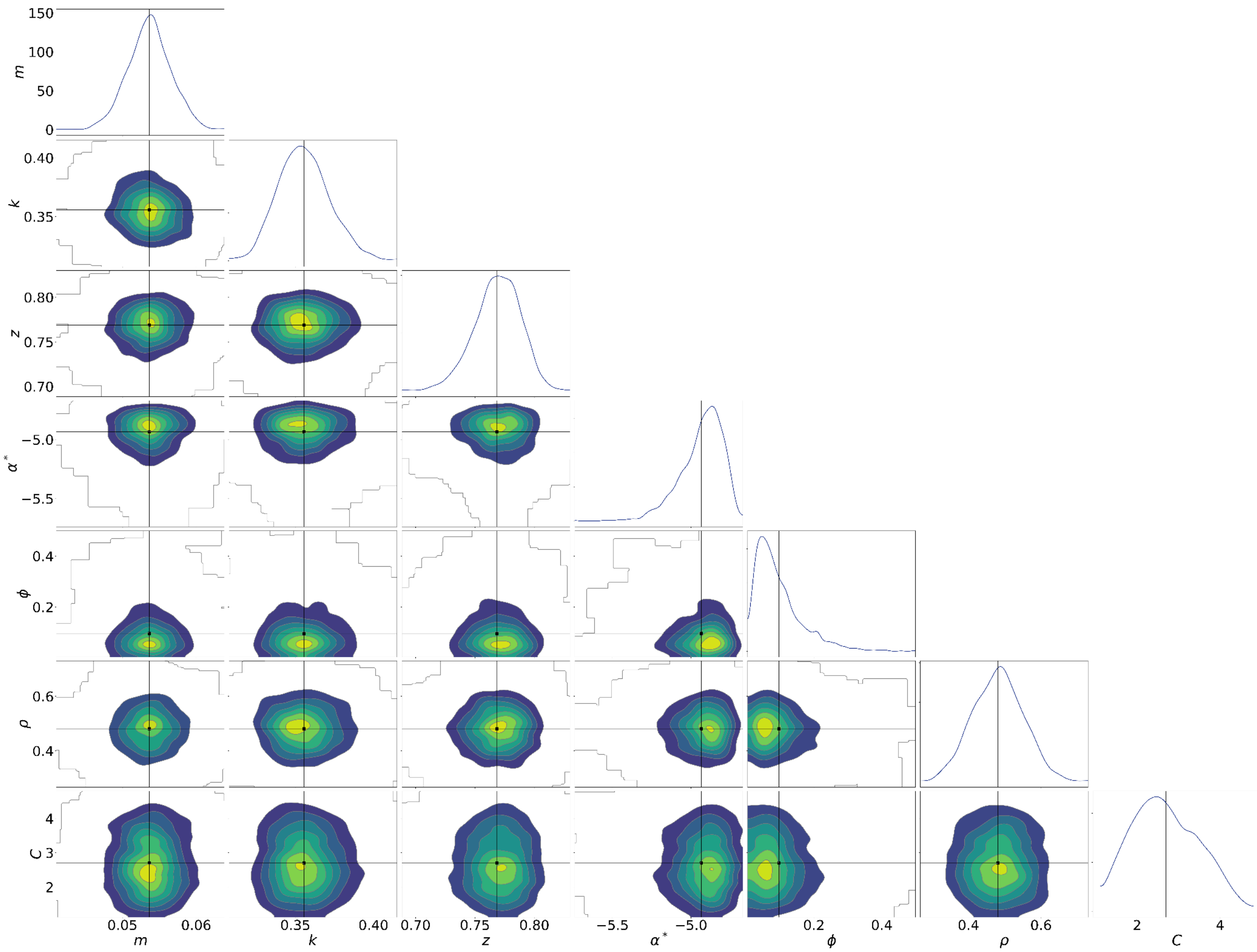

Fig I. Posterior pair plots for the group-level parameters. The contours show the density of the posterior pairs. The diagonal shows the marginal posteriors.

Table A. Summary of posterior parameter estimates across all groups. Note that α denotes the back-transformed (via *exp* function) parameter space.

Parameter	Mean	Std. dev.	94% HDI
**HC**			
m	0.0536	0.0029	[0.0482, 0.0590]
k	0.3556	0.0164	[0.3277, 0.3879]
z	0.7687	0.0194	[0.7301, 0.8016]
θ	0.0007	0.0017	[0.0000, 0.0023]
α	0.0073	0.0009	[0.0055, 0.0088]
ϕ	0.0964	0.0692	[0.0107, 0.2266]
ρ	0.4812	0.0687	[0.3552, 0.6119]
γ	0.0966	0.1061	[0.0010, 0.2971]
ϵ	0.2724	0.0356	[0.2038, 0.3371]
C	2.6940	0.7879	[1.3879, 4.1681]
**SCZ**			
m	0.0408	0.0035	[0.0341, 0.0472]
k	0.3946	0.0273	[0.3443, 0.4454]
z	0.8136	0.0387	[0.7432, 0.8725]
θ	0.0010	0.0019	[0.0000, 0.0036]
α	0.0045	0.0004	[0.0038, 0.0051]
ϕ	0.3658	0.0299	[0.3097, 0.4219]
ρ	0.4680	0.0364	[0.3950, 0.5326]
γ	0.0646	0.0677	[0.0017, 0.1857]
ϵ	0.3167	0.0356	[0.2514, 0.3836]
C	2.0537	0.6483	[1.1187, 3.2671]
**BP**			
m	0.0451	0.0043	[0.0378, 0.0536]
k	0.3833	0.0240	[0.3395, 0.4290]
z	0.7599	0.0226	[0.7188, 0.8012]
θ	0.0013	0.0029	[0.0000, 0.0042]
α	0.0060	0.0008	[0.0046, 0.0074]
ϕ	0.2794	0.0559	[0.1750, 0.3855]
ρ	0.4566	0.0473	[0.3662, 0.5437]
γ	0.0926	0.0982	[0.0019, 0.2673]
ϵ	0.2861	0.0311	[0.2250, 0.3407]
C	1.9004	0.5492	[1.0896, 2.9037]
**MDD**			
m	0.0511	0.0035	[0.0447, 0.0576]
k	0.3265	0.0238	[0.2822, 0.3692]
z	0.7392	0.0162	[0.7097, 0.7692]
θ	0.0010	0.0020	[0.0000, 0.0032]
α	0.0069	0.0009	[0.0053, 0.0085]
ϕ	0.1208	0.0875	[0.0096, 0.2861]
ρ	0.4431	0.0357	[0.3798, 0.5149]
γ	0.1550	0.1338	[0.0061, 0.4133]
ϵ	0.3028	0.0305	[0.2449, 0.3586]
C	3.2337	0.7565	[1.7758, 4.4999]

Table B. Summary of contrasts across all groups. Note that α^* denotes the transformed (via *log* function) parameter space.

Parameter	Mean	Std. dev.	94% HDI
**Contrast: HC - SCZ**			
m	0.0128	0.0045	[0.0049, 0.0218]
k	-0.0390	0.0324	[-0.1005, 0.0204]
z	-0.0449	0.0433	[-0.1173, 0.0395]
θ	-0.0003	0.0025	[-0.0046, 0.0028]
α^*	0.4805	0.1588	[0.1856, 0.7629]
ϕ	-0.2695	0.0756	[-0.3921, -0.1196]
ρ	0.0132	0.0784	[-0.1328, 0.1538]
γ	0.0320	0.1265	[-0.1865, 0.3156]
ϵ	-0.0443	0.0504	[-0.1411, 0.0486]
C	0.6402	1.0060	[-1.1827, 2.5255]
**Contrast: HC - BP**			
m	0.0085	0.0051	[-0.0014, 0.0178]
k	-0.0276	0.0293	[-0.0806, 0.0286]
z	0.0088	0.0300	[-0.0508, 0.0627]
θ	-0.0005	0.0034	[-0.0049, 0.0031]
α^*	0.1929	0.1894	[-0.1535, 0.5509]
ϕ	-0.1831	0.0899	[-0.3356, 0.0079]
ρ	0.0246	0.0842	[-0.1328, 0.1795]
γ	0.0040	0.1451	[-0.2819, 0.2983]
ϵ	-0.0138	0.0476	[-0.1073, 0.0702]
C	0.7936	0.9606	[-1.0528, 2.5669]
**Contrast: HC - MDD**			
m	0.0025	0.0046	[-0.0059, 0.0110]
k	0.0291	0.0285	[-0.0225, 0.0836]
z	0.0295	0.0255	[-0.0202, 0.0755]
θ	-0.0003	0.0026	[-0.0041, 0.0025]
α^*	0.0534	0.1878	[-0.2954, 0.4047]
ϕ	-0.0245	0.1127	[-0.2480, 0.1943]
ρ	0.0381	0.0775	[-0.1028, 0.1901]
γ	-0.0584	0.1692	[-0.4060, 0.2810]
ϵ	-0.0304	0.0471	[-0.1165, 0.0595]
C	-0.5398	1.0871	[-2.5651, 1.4968]

Table C. Demographic information about the groups.

	Groups			
	**HC**	**MDD**	**BP**	**SCZ**
**Mean (SD) age in years**	34 (10)	32 (9)	37 (11)	36 (11)
**Gender (M:F:other)**	40:46:01	23:27:04	19:27:01	40:19:06
**Race**				
white	48	37	34	33
black/african american	23	11	10	26
indian (american)	1	1	2	2
asian	8	1	1	1
**Mean (SD) school years subject**	16 (2)	16 (2)	16 (3)	14 (3)
**Mean (SD) school years mother**	14 (4)	14 (3)	14 (3)	15 (3)
**Mean (SD) school years father**	14 (4)	15 (3)	15 (4)	15 (3)
**Medication status**				
antipsychotic	0	3	21	51
mood stabilizer	0	3	23	12
antidepressant	1	37	25	26
antianxiety	3	14	13	13
**Mean (SD) standardized IQ scores derived from the Wechsler Test of Adult Reading (WTAR-FSIQ)**	113 (12)	112 (12)	112 (11)	104 (16)

References

1. Ging-Jehli NR, Rac-Lubashevsky R, Bera K, Boudewyn MA, Carter CS, Erickson MA, et al. Model-based electroencephalography phenotyping uncovers distinct neurocomputational mechanisms underlying learning impairments across psychopathologies. Biol Psychiatry Glob Open Sci. 2026;6(2):100660. doi:10.1016/j.bpsgos.2025.100660
