# Supplementary material for: Modeling decision dynamics disentangles working memory, cognitive control and reinforcement learning and reveals clinical differences
Source: PLoS Comput Biol. 2026 Sep 30;22(9):e1014796. doi: 10.1371/journal.pcbi.1014796 (PMC13660991; doi:10.1371/journal.pcbi.1014796)
Supplement: Supplementary file 2 [file pcbi.1014796.s003.docx]

We were pleased with the positive and constructive reviews. We have addressed all the comments, and the manuscript has benefited substantially from this process.

**Editor:**

In particular, I suggest you carefully motivate your experimental design (cf. RLWM task) for the specific aim of discriminating schizophrenic patients from control participants w.r.t. their RL learning rate. I also believe (as R2 highlights) that you should report results from (model-free and/or model-based) analyses of the test phase. You will find the reviewer's reports below.

**Response to the Editor:**

We thank the Editor for these suggestions, both of which we have addressed in the revision.

First, we have clarified why the RLWM paradigm is well suited to discriminate differences in patient groups with respect to RL learning rate. The central issue is that there are many “reinforcement learning” tasks but the vast majority of them do not discriminate whether RL adaptations are due to incremental learning from reward prediction errors (as is often assumed either explicitly or implicitly) versus those that are mediated by WM contributions to RL. Our previous work had suggested that such WM contributions can dominate trial-by-trial adjustments during learning, thought to reflect prefrontal activation dynamics, and that slower RL adaptations (e.g. due to corticostriatal synaptic plasticity) are better revealed by a test phase (Frank & Claus 2006; Frank et al., 2007). This observation motivated the development of the RLWM paradigm to explicitly manipulate WM load during learning, so that one can simultaneously estimate WM and RL processes jointly during learning (Collins & Frank, 2012 and subsequent papers). Previous work in schizophrenia found that SCZ learning impairments were primarily due to WM contributions, with relatively spared RL learning rate. However, the current report shows that despite this design and explicit model, when the RLWM model is fit only to choices, there remains a confounding bias to estimate RL learning rates. The PADDL model addresses this concern by using RT distributions to further disentangle the processes, improving RL recovery, and allowing us to reveal clinical differences not only in WM but also in RL. We now make this rationale explicit and scope the clinical claim to the incremental acquisition rate, as detailed in our response to Reviewer 2 (point 1b) and in the revised Introduction and Results.

Second, we very much agree that the test phase is an independent measure of RL (based also on our earlier work highlighted above). We have now added an out-of-sample analysis of the held-out test phase, which provides a WM-free read-out of retained RL value. Strikingly, we find that the RL learning rates estimated from PADDL provide an excellent fit to test phase choices even though these were not used for fitting. In contrast, the choice-only model overestimates participants’ sensitivity to RL values during the test phase, consistent with our findings that the choice-only model inflates RL learning rates. This provides independent validation of the PADDL learning rate estimates that underlie the clinical contrast. The analysis appears in the Results (section “PADDL estimated RL predicts out-of-sample test phase choices,” Fig. 4) and Methods (section “Test phase analysis and modeling (Out-of-sample validation)”), with the SCZ counterpart in the Supplement (Fig. S3). Full details are given in our responses to Reviewer 2 (points 1b and 1c).

The point-by-point responses to the reviewers follow.

**Reviewer #1:**

The study entitled “Modeling decision dynamics disentangles working memory, cognitive control and reinforcement learning and reveals clinical differences” by Bera and colleagues combines computational models of reinforcement learning, evidence accumulation, and working memory to examine decision-making in healthy controls and clinical groups. The study uses an established reinforcement learning working memory (RLWM) task and extends previous joint modeling of these two components by adding a linear ballistic accumulator to integrate response times into the model. Model estimation relies on advanced simulation-based inference. Next to replicating previous results on the interplay of learning and working memory, the study identifies a novel proactive control process with more cautious responding under higher working memory load. This mechanism, in addition to lower learning rates, is suggested to be altered in patients with schizophrenia compared to healthy controls.

Strengths

(1) Advanced computational model that combines elements of reinforcement learning, working memory, and evidence accumulation

(2) Advanced simulation-based inference approach and convincing validation analyses including parameter recovery and posterior-predictive checks

(3) The study includes patients and control subjects and provides evidence for computational differences in schizophrenia patients

Weaknesses

(1) Fixed beta parameter of softmax function

The beta parameter of the softmax was fixed at 100. This is only very briefly justified, and raises the question of whether this artificially lowers the goodness-of-fit of the choice-only model compared to models with LBA. Was the parameter also fixed for the choice-only model, and if so, why?

Yes, the inverse-temperature parameter β was fixed for the choice-only model as well, for several reasons. First, this is actually common practice for the RLWM model (see previous papers cited in the manuscript), given that the choice function (in both the standard model and PADDL) already contains an epsilon parameter for decision noise, and allowing β to vary severely degrades parameter recovery as it trades off with other parameters such as ε and ρ. Second, despite fixing β, the choice-only fits remain flexible enough to capture the choice distributions, as is evident from comparing the PADDL and choice-only posterior predictive checks. Third, we additionally ran a parameter recovery for a choice-only model with free β; recovery remained poor across all parameters relative to the PADDL model (shown below).

Figure 1: Posterior predictive checks for PADDL and choice-only models with fixed inv. temp. parameter.

Figure 2: Parameter recovery comparison: PADDL and choice-only softmax model with freely estimated inv. temp. parameter.

(2) Relationship between p(a|s) and pol

On page 16, the authors explain the model. I was wondering whether eq. 4 showing p(a|s) is basically used for computing the “pol_RL” and “pol_WM” variables?

Yes, that is correct – expression p(a|s) gives individual probability mass assigned to each action. The tuple [p_a1, p_a2, p_a3] gives the policy. We now elaborate to provide this clarification in the text.

(3) Parameter symbols versus names

Especially in the main manuscript (e.g., Figure 2), the authors do not clearly link the parameter symbols and the function of the parameters. Although symbols and functions are both mentioned in Figure 1, I found it difficult to understand Figure 2 before I had read the methods in detail.

We have now introduced parameter names alongside the symbols in the figure to improve readability.

(4) Low learning rates

I was surprised that RL learning rates were quite small (e.g., 0.0087, 0.0047). Can the authors please explain this, and whether behavioral flexibility is more strongly driven by the working memory component?

We thank the reviewer for this question, which has helped us to clarify this issue. Standard RL models often estimate RL learning rates to account for trial-by-trial adaptation without distinguishing the WM contribution to RL. But here, the small learning rates are expected given that the RLWM paradigm was constructed specifically to dissociate a slow, incremental reinforcement learning process from fast, flexible WM. In this framework, the learning rate α governs only the slow, model-free component, canonically supported by incremental value updating in the striatum, whereas the rapid trial-to-trial flexibility of behavior is carried by the faster PFC-based WM system (O'Reilly & Frank, 2006). The reviewer is therefore correct that rapid behavioral flexibility is driven more strongly by WM, and this observation in previous work (e.g. Frank et al., 2007) motivated the RLWM paradigm in the first place, whereby the slow RL component is expected to contribute appreciably only once WM capacity is exceeded, at higher set sizes and later trials (Collins & Frank, 2012 and subsequent papers). (We also note that the specific values cited by the reviewer (0.0087 and 0.0047) reflected a transcription error at a single location in the originally submitted manuscript; the correct estimates, 0.0073 (HC) and 0.0045 (SCZ), were reported at two other locations and are now stated consistently throughout the revised manuscript and Table S1. The statistics do not change as they were reported using the correct posterior distributions).

Importantly, although α is small, it exerts a clear and measurable influence on behavior, which is what makes it identifiable. Because the softmax inverse temperature was fixed at β = 100, the effective change in the RL policy is appreciable (since the changes in Q values are magnified by 100), such that even a small learning rate produces a separation in learned values that is expressed in both the choice and RT distributions, and most visibly at higher set sizes and later trials, where the WM contribution is largely exhausted and the RL process dominates. The ablation experiments in Figure 3 (A,B,D) make this concrete: overestimating α produces value separation that is too rapid and therefore predicts RTs that decline faster than observed, with the discrepancy growing at higher set sizes. To further demonstrate the RL contributions, we now show what happens when RL learning rates in PADDL are set to zero. With no RL contribution, the model fails to reproduce the empirical accuracy curves, with the largest discrepancy at higher set sizes, and predicts systematically slower RTs that depart from the observed trends. Together, these analyses show that the small but non-zero learning rate is necessary to account for the data and is well constrained by the joint choice-RT fit.

Figure 3: Posterior predictives (accuracy and RT curves) without the learning rate contributions (alpha=0).

(5) The results in schizophrenia patients are convincing. However, I was wondering what role their usually reduced cognitive functioning could play here? Might the results be driven by lower cognitive ability in patients? I think it would be relevant to add a paragraph on this to the discussion.

We agree that schizophrenia is associated with broadly reduced cognitive functioning, and that this raises the legitimate question of whether the parameter differences we report are specific or instead a downstream reflection of general impairment. We would first note that reduced cognitive functioning in schizophrenia is itself a composite, characterized along separable dimensions including reinforcement learning, working memory, and cognitive control. A central aim of the RLWM paradigm and the PADDL model is to decompose this broad construct into candidate computational primitives, so that cognitive function can be characterized in terms of specific, mechanistically interpretable processes rather than a single global factor.

Critically, the pattern we observe is selective rather than uniform. The SCZ group shows credible reductions in RL learning rate (α) and proactive control (m), and elevated WM decay (φ), but no credible differences in WM capacity (C), WM reliance (ρ), perseveration (γ), or undirected noise/lapse rate (ε). A purely general-ability account would predict diffuse impairment across parameters, and in particular an elevated lapse rate, which is the canonical computational signature of global disengagement or reduced effort. The absence of any credible difference in ε, alongside intact ρ, γ, and C, argues against a uniform decline and instead localizes the deficit to specific learning and control mechanisms. We have added a paragraph to the Discussion making this point.

We now extend the discussion of clinical contrasts with the following –

These deficits in SCZ group could in principle stem from the broadly reduced cognitive functioning in that group. We note, however, that such functioning is itself composed of separable primitives spanning RL, WM, and cognitive control, and that the PADDL model is intended to identify which of these are affected. Here, reduced RL learning rate, weakened proactive control, and faster WM decay emerge as the specific contributors, while capacity, perseveration, and noise remain intact (i.e., no credible difference is found), indicating a selective rather than global pattern.

(6) The data are only available upon reasonable request. Is there a particular reason why the authors don’t share their pseudonymized data in a repository? If there are no data protection issues that speak against this, I would recommend that they openly share the data.

Our previous data availability statement was incorrect since the dataset is already publicly available. The data are publicly available on the NIMH national data archive and can be accessed there at <https://nda.nih.gov/edit_collection.html?id=3321>. We have updated the data availability statement to reflect this.

**Reviewer #2:**

In this study, the authors present a novel computational framework for jointly modeling choice and response time in the Reinforcement Learning + Working Memory (RLWM) paradigm. Using a 3AFC task on a dataset of 255 participants including controls and patients with schizophrenia, the authors report three main findings: joint modeling improves parameter recovery over choice-only models, participants proactively adjust decision thresholds as a function of working memory load, and schizophrenia patients show a reduced reinforcement learning rate that was missed by prior choice-only analyses. The question of jointly modeling choices and RTs is timely and will be of broad interest to the decision-making community. The work has many strong positive features, including a real methodological contribution, a large sample size, and careful recovery procedures. I therefore have only a few concerns about the modeling and its interpretation, which in its current form leave a few questions open.

1) The RLWM paradigm was designed to dissociate RL from WM under WM-load manipulation, but it is not designed to interrogate RL itself.

1a) A few things about the outcome contingency structure are unclear. The Methods state that correct responses are "probabilistically reinforced, yielding either one or two points," but it is not clear whether this means (a) correct responses are always rewarded with a probabilistic magnitude (1 or 2 points), or (b) reward delivery itself is probabilistic and some correct responses get zero. The two have very different implications for what the learning rates are actually learning from. The model collapses outcomes to binary coding (0/1), but this disregards the magnitude information the participants actually saw (see the broader literature on outcome normalization). Could the authors describe the contingency structure clearly, and give a stronger reason for the binary coding than "prior analysis showed no improvement"?

The (a) interpretation is correct, the correct responses were always rewarded with a probabilistic magnitude (1 or 2 points) and the incorrect responses always yielded zero points. We have now clarified this in the manuscript. We assume a binary reward coding for modeling because our previous Collins et al. 2017 study showed no performance difference with high, medium, or low probability of reward 2 points vs. 1 point when correct choice is made (Fig 2D, Collins et al. 2017). The reason is probably that the participants know there is only one correct response for each stimulus, so the best they can do is find that rewarding response. In theory, they could have prioritized some stimulus in WM if they had higher p(reward=2 pts) but the data does not support that account. We now clarify this in the manuscript.

1b) If I understand correctly, the task consists of 10 iterations per stimulus, deterministic stimulus-action mappings, no reversals or volatility, and a test phase that is excluded from modeling. This raises the concern that the data available to constrain learning rates come from only a small part of the task, which would fit with how small the recovered values are. The methodological contribution that joint modeling improves identifiability under RL/WM competition does not need an ideal RL task, and RLWM is the right design for that claim. But the clinical claim about the RL learning rate in schizophrenia depends on the learning rates being well-identified, and the way the task is built makes that hard. Could the authors say more about what RLWM does and does not imply about RL, and explain why they did not use a task with richer RL structure (probabilistic contingencies, reversals, and so on) in which the design would constrain the learning rates across more than just a few early trials?

We thank the reviewer for this thoughtful comment, which helped us to clarify our contributions and assumptions, and in particular for distinguishing the methodological claim, which RLWM is well suited to support, from the clinical claim, which requires the learning rates to be identified. We address the terminology, what RLWM does and does not imply about RL, why a richer RL design would not better serve the present aim, and the evidence that the rates are in fact accurately identified.

First, a clarification of terms (see also response to R1). Throughout this work, "RL" refers specifically to the slow, incremental, model-free component supported by reward prediction errors (canonically, dopaminergic striatal learning), as distinct from the fast, flexible component we attribute to WM. This is narrower than "RL" as an umbrella for value-learning tasks as a whole, in which both fast and slow contributions are present. The reviewer is correct that the recovered learning rates for this slow component are small, but this is expected by construction rather than a symptom of weak constraint: in this paradigm the fast WM system handles most early and low-load adaptation, and the incremental RL component contributes appreciably only once WM capacity is exceeded at higher set sizes and later trials.

This bears directly on why a task with richer RL structure (probabilistic contingencies, reversals, volatility) would not better constrain this particular component. Such manipulations are thought to preferentially recruit fast, flexible adaptation, including WM-based updating, which dominates trial-to-trial behavior and can mask, rather than isolate, the slow incremental rate. This conflation is precisely the problem that motivated the RLWM paradigm. In earlier work using probabilistic contingencies, behavior reflected both fast and slow learning components that were difficult to disentangle during acquisition, because most trial-to-trial adaptation is driven by the fast system, leaving the slow RL contribution hard to observe directly (Frank et al., 2007). The RLWM task was developed specifically to address this by manipulating WM load through set size, exposing the slow RL contribution during learning itself (Collins & Frank, 2012), and a substantial subsequent literature has used it to this end (Collins et al., 2014; Collins & Frank, 2018; Rac-Lubashevsky et al., 2023). In this sense RLWM is not a compromise relative to a richer RL design for our purpose; it is the design that makes the incremental component estimable in the first place.

On the concern that the rates are constrained by "only a small part of the task": while there are only 10 trials per stimulus, there are multiple stimuli and multiple set sizes, each of which have multiple blocks of trials, and it is this design that allows the RLWM model to isolate the RL contributions. With joint modeling, we can see that the RL process influences the RT slopes across the full learning trajectory in higher set sizes, where RL is relatively more dominant, providing an additional and continuous source of constraint (Figs 3, 5), which is one reason joint modeling improves identifiability over choice-only fits.

Moreover, in response to this comment, **we now provide a strong independent test that the PADDL model has identified the RL process** despite fitting only to the learning phase (which we view as a feature rather than a bug in that earlier work required a separate “fit-to-test” procedure to identify RL (Frank et al., 2007), which again motivated the RLWM paradigm). Following these suggestions, **we have now added an analysis of the held-out test phase**, which provides a WM-free, out-of-sample criterion that the model was never fit to (see details below). Strikingly, we find that the RL process implied by the PADDL learning rate can be used to closely predict test phase choice accuracy, whereas those implied by the choice-only model, with its inflated rate, systematically overpredict test accuracy (new Fig 4 and Results). Because the test phase indexes retained value with negligible WM involvement, this independently confirms that the lower learning rate estimates reflect the true incremental RL signal rather than under-identification. This result complements our other findings showing that (i) parameter recovery shows that the learning rate is recovered significantly better under the joint than the choice-only model (Z = 2.709, p = 0.0067), and (ii) that the RL process can be linked to a specific signature of RT slopes across set sizes, which is altered in SCZ, and is abolished when the HC and SCZ rates are equalized (Fig 5 C).

Figure 4: Out-of-sample validation of estimated parameters on the held-out test phase (HC group). (A) Probability of choosing the higher-valued stimulus. (B) Psychometric function of choosing the right-hand stimulus against the signed value difference.

Finally, on what RLWM does and does not imply: it supports estimation of the rate of incremental acquisition during learning under controlled WM load, and our clinical claim is scoped to that quantity. It does not speak to RL flexibility under change (reversals, volatility) or to a domain-general RL deficit, and we do not interpret it as such.

1c) Excluding the test phase from modeling is justified on the grounds that prior work found no clinical effects there. But the test phase provides the cleanest read-out of long-term RL retention without WM contributions and adding it to the joint modeling could provide an independent validation of the learning rates estimates.

We thank the reviewer for this suggestion, which we agree improves the paper as noted above. We share the reviewer's view that the test phase offers a WM-free read-out of retained RL value, and the revised manuscript now uses it for exactly the purpose the reviewer identifies: an independent validation of the learning rate estimates. This analysis appears in the Results (section " PADDL estimated RL predicts out-of-sample test phase choices," Fig. 4) and the Methods (section "Test phase analysis and modeling"). The headline result is that the learning rates estimated from the learning phase alone predict held-out test phase choices well for PADDL but not for the choice-only model, providing independent support for the lower PADDL learning rates that underlie the clinical contrast.

We would highlight one distinction relative to the reviewer's framing of "adding it to the joint modeling." We deliberately did not jointly fit the train and test phases, because doing so would allow the test data to influence the learning rate estimates and would therefore no longer constitute an independent validation of those estimates. Instead we hold all parameters fixed at their learning phase values and ask whether they predict the test phase out of sample, which is a stricter and more genuinely independent criterion (especially in that we only did this after the reviewer’s suggestion and using the same model that was fitted for the first version of the manuscript without any alteration). In particular, for each participant we used the final stimulus values (or Q_RL values weighted by the frequency of chosen action) learned by the RL system at the end of training (based on their learning rates), and used those to predict test choices from these values with no parameters fit to the test data (Methods, Eq. 9).

We did not fit the RTs of the test phase given that it required a different kind of choice altogether (i.e., it did not require participants to select one of three responses for each stimulus but instead presented pairs of stimuli and asked them to choose the one they believed to have been more rewarding (without feedback). It is therefore naturally modeled by mapping the learned stimulus values through a decision rule. We use a softmax choice rule applied identically to both PADDL and the choice-only model, for the following reasons:

- The test phase RT distribution differs substantially from the learning phase in its summary statistics (minimum, median, maximum), so learning phase LBA-Angle parameters are unlikely to transfer directly. This distributional shift is also consistent with the involvement of additional processes at test, (speculatively) for example retrieval or episodic contributions, distinct from the within-trial dynamics estimated during learning.
- The accumulator architecture changes from a three-alternative race during learning to a two-alternative comparison at test, so the learning phase decision geometry has no direct analogue.
- The learning phase threshold is an explicit function of set size (a = m·ns + k), but test stimuli are drawn across blocks, leaving no principled value of ns and hence no defined (m, k) parameterization at test.
- A softmax mapping raises its own open questions that we judged premature to resolve, for example whether the inverse temperature should differ at test and whether it should be jointly estimated with the learning phase. Rather than introduce these free choices, we simply applied the same choice rule that had been used during learning fixing the parameters without alteration (including β = 100 and the estimated lapse parameter ε from learning), for both models.

In short, we chose the minimal, assumption-light decision rule common to both models. Because the rule is held fixed across models, any difference in predicted test accuracy is attributable to the learned values each model implies rather than to the decision process. This is precisely the property needed for the validation the reviewer requests.

The results confirm the value of this analysis. The choice-only softmax model systematically overpredicts test accuracy, with predictions exceeding the 94% HDI of the empirical estimates at the medium and high value-difference bins and a psychometric function that is too steep through indifference, the signature of over-separated learned values. PADDL tracks the empirical curve closely. Quantified as the signed deviation of predicted from empirical accuracy, the choice-only model is biased upward (mean deviation = 0.0488) whereas PADDL is approximately unbiased and in the opposite direction (mean deviation = -0.0190). Aggregate per-trial log-loss favors PADDL but does not reach significance (mean difference = 0.0048 ± 0.0067). This pattern is the direct out-of-sample consequence of the learning rate overestimation by the choice-only model documented earlier (Figs. 2 and 3): an inflated learning rate yields overly separated values that overstate discriminability at test. That PADDL predicts the held-out test phase accurately, using parameters estimated solely from the learning phase, indicates its lower learning rate estimates reflect the true incremental RL signal rather than under-identification.

2) Figure 1C is currently hard to read. It would benefit from more explicit labeling and a consistent color code linking each parameter to the visual element it governs. More generally, the manuscript in its current form will be difficult for any reader who is not already fluent in all of: the RLWM paradigm, the LBA-Angle variant, LANs and simulation-based inference, hierarchical ADVI, Savage–Dickey ratios (which I myself was not familiar with), and the clinical literature on SCZ-RL. A short glossary box or plain-language opening paragraphs would make the paper much easier to get into, without making it any less rigorous.

We thank the reviewer for this suggestion. We agree the manuscript drew on several technical literatures without orienting non-specialist readers, and we have addressed this by adding two explicit reading aids without altering any analysis. We added a model parameters box (Box 1, end of Introduction) to introduce a model legend that shares its symbols and color key with the revised Figure 1C. We also add a glossary box (Box 2, end of Discussion) defining every flagged concept (RLWM, LBA and LBA-Angle, LANs and simulation-based inference, hierarchical ADVI, Savage–Dickey ratio). The figure now has more explicit labeling and a consistent color palette. The figure makes it easy to identify the three modules and the parameters therein in the computational model – RL, WM and decision process.

We also added brief plain-language definitions at the first occurrence of each term:

- **RLWM dissociation** (Results, ll. 150-152): now states that WM, but not RL, is sensitive to set size and delay.
- **LBA / LBA-Angle** (Results, ll. 169-170/175-178): abbreviation introduced at first mention; Angle variant glossed as a within-trial collapsing boundary.
- **LANs / SBI** (Introduction, ll. 125-127): defined as simulation-trained networks approximating likelihoods that lack a closed form.
- **Hierarchical estimation** (Discussion, ll. 490-492): clause added on joint individual- and group-level estimation with regularization.
- **Savage–Dickey ratio** (Methods, ll. 762-765): self-contained definition added, with the directional reading needed to interpret the reported B01 values.
- **SCZ-RL literature** (Introduction, ll. 66-68/138-141): now signals that joint modeling qualifies the prior consensus by additionally revealing a reduced RL learning rate.

3) Several methodological choices carry a lot of weight but are introduced in one or two sentences. The ablation experiments in Figure 3 are the main evidence for the paper's claims, but the reader has to work out from the figures why each ablation is the right comparison to run.

We thank the reviewer for this observation. We agree that the ablations in Figure 3 carry much of the paper's argument and that their rationale was under-explained. We have revised the Results so that the logic of each ablation is stated explicitly rather than left to the figures. We now introduce both ablations with a single design principle: each removes or replaces one mechanism of the joint model while holding all other parameters fixed, so that any misfit to the RT data isolates that mechanism (Results, ll. 254-258; Fig. 3 caption). For the learning rate ablation we now state that the two simulations differ only in α, and that later trials and higher set sizes are the diagnostic window because RL, and therefore sensitivity to α, dominates when WM capacity is exceeded (Results, ll. 259-266). For the proactive-control ablation we now make the nesting argument explicit, namely that the static-bound model is the strict m = 0 special case of PADDL and therefore the correct comparison, and we explain why set-size differences in RT during the earliest trials of a block, before any feedback could have driven learning, can only arise from a threshold set in anticipation of load (Results, ll. 326-336). The figure caption now states the design principle and the mechanism each panel isolates.

4) While the authors acknowledge that joint choice-RT modeling has been attempted before and explain why their model improves on it, some relevant literature, both in learning (e.g., Miletić et al., eLife 2021) and non-learning (e.g., Clithero, J. Econ. Behav. Organ. 2018) contexts, complements the authors' claims and should be cited.

We have added the recommended references, along with two further complementary works, and cited them in the Introduction and Discussion: Miletić et al. (2021), Luzardo et al. (2017) and Sewell et al. (2019) on joint choice-RT modeling in learning contexts, and Clithero (2018) on the out-of-sample predictive value of response times in non-learning, value-based choice.

**References:**

O'Reilly, R. C., & Frank, M. J. (2006). Making working memory work: a computational model of learning in the prefrontal cortex and basal ganglia. Neural computation, 18(2), 283-328.

Frank, M. J., Moustafa, A. A., Haughey, H. M., Curran, T., & Hutchison, K. E. (2007). Genetic triple dissociation reveals multiple roles for dopamine in reinforcement learning. Proceedings of the National Academy of Sciences, 104(41), 16311-16316.

Frank, M. J., & Claus, E. D. (2006). Anatomy of a decision: striato-orbitofrontal interactions in reinforcement learning, decision making, and reversal. Psychological review, 113(2), 300.

Collins, A. G., & Frank, M. J. (2012). How much of reinforcement learning is working memory, not reinforcement learning? A behavioral, computational, and neurogenetic analysis. European Journal of Neuroscience, 35(7), 1024-1035.

Collins, A. G., Brown, J. K., Gold, J. M., Waltz, J. A., & Frank, M. J. (2014). Working memory contributions to reinforcement learning impairments in schizophrenia. Journal of Neuroscience, 34(41), 13747-13756.

Collins, A. G., Albrecht, M. A., Waltz, J. A., Gold, J. M., & Frank, M. J. (2017). Interactions among working memory, reinforcement learning, and effort in value-based choice: A new paradigm and selective deficits in schizophrenia. *Biological psychiatry*, *82*(6), 431-439.

Collins, A. G., & Frank, M. J. (2018). Within-and across-trial dynamics of human EEG reveal cooperative interplay between reinforcement learning and working memory. Proceedings of the National Academy of Sciences, 115(10), 2502-2507.

Rac-Lubashevsky, R., Cremer, A., Collins, A. G., Frank, M. J., & Schwabe, L. (2023). Neural index of reinforcement learning predicts improved stimulus–response retention under high working memory load. Journal of Neuroscience, 43(17), 3131-3143.

Miletić, S., Boag, R. J., Trutti, A. C., Stevenson, N., Forstmann, B. U., & Heathcote, A. (2021). A new model of decision processing in instrumental learning tasks. eLife, 10, e63055.

Luzardo, A., Alonso, E., & Mondragón, E. (2017). A Rescorla-Wagner drift-diffusion model of conditioning and timing. PLOS Computational Biology, 13(11), e1005796.

Sewell, D. K., Jach, H. K., Boag, R. J., & Van Heer, C. A. (2019). Combining error-driven models of associative learning with evidence accumulation models of decision-making. Psychonomic Bulletin & Review, 26(3), 868-893.

Clithero, J. A. (2018). Improving out-of-sample predictions using response times and a model of the decision process. Journal of Economic Behavior & Organization, 148, 344-375.
