# Supplementary material for: Modeling decision dynamics disentangles working memory, cognitive control and reinforcement learning and reveals clinical differences
Source: PLoS Comput Biol. 2026 Sep 30;22(9):e1014796. doi: 10.1371/journal.pcbi.1014796 (PMC13660991; doi:10.1371/journal.pcbi.1014796)
Supplement: Peer review history

# Peer review: Decision Letter 0

**DOI:** 10.1371/journal.pcbi.1014796.r001

**Authors:** Jean Daunizeau, Daniele Marinazzo

1 Jun 2026

Modeling decision dynamics disentangles working memory, cognitive control and reinforcement learning and reveals clinical differences

Dear Dr. Bera,

Please submit your revised manuscript by Aug 01 2026 11:59PM. If you will need more time than this to complete your revisions, please reply to this message or contact the journal office at ploscompbiol@plos.org. When you're ready to submit your revision, log on to https://www.editorialmanager.com/pcompbiol/ and select the 'Submissions Needing Revision' folder to locate your manuscript file.

Please include the following items when submitting your revised manuscript:

* A letter that responds to each point raised by the editor and reviewer(s). You should upload this letter as a separate file labeled 'Response to Reviewers'. This file does not need to include responses to formatting updates and technical items listed in the 'Journal Requirements' section below.

* A marked-up copy of your manuscript that highlights changes made to the original version. You should upload this as a separate file labeled 'Revised Manuscript with Track Changes'.

* An unmarked version of your revised paper without tracked changes. You should upload this as a separate file labeled 'Manuscript'.

We look forward to receiving your revised manuscript.

Kind regards,

Jean Daunizeau

Academic Editor

PLOS Computational Biology

Daniele Marinazzo

Section Editor

PLOS Computational Biology

**Journal Requirements:**

1) Please ensure that the CRediT author contributions listed for every co-author are completed accurately and in full.

At this stage, the following Authors/Authors require contributions: Krishn Bera, Alexander Fengler, Megan Boudewyn, Cameron Carter, Molly Erickson, James Gold, Steven Luck, J. Daniel Ragland, Andrew Yonelinas, Angus MacDonald III, Deanna Barch, and Michael J. Frank. Please ensure that the full contributions of each author are acknowledged in the "Add/Edit/Remove Authors" section of our submission form.

The list of CRediT author contributions may be found here: https://journals.plos.org/ploscompbiol/s/authorship#loc-author-contributions

2) We ask that a manuscript source file is provided at Revision. Please upload your manuscript file as a .doc, .docx, .rtf or .tex. If you are providing a .tex file, please upload it under the item type u2018LaTeX Source Fileu2019 and leave your .pdf version as the item type u2018Manuscriptu2019.

3) Please upload all main figures as separate Figure files in .tif or .eps format. For more information about how to convert and format your figure files please see our guidelines:

https://journals.plos.org/ploscompbiol/s/figures

4) We notice that your supplementary Figures, and Tables are included in the manuscript file. Please remove them and upload them with the file type 'Supporting Information'. Please ensure that each Supporting Information file has a legend listed in the manuscript after the references list.

5) Please amend your detailed Financial Disclosure statement. This is published with the article. It must therefore be completed in full sentences and contain the exact wording you wish to be published.

- State the initials, alongside each funding source, of each author to receive each grant. For example: "This work was supported by the National Institutes of Health (####### to AM; ###### to CJ) and the National Science Foundation (###### to AM)."

- State what role the funders took in the study. If the funders had no role in your study, please state: "The funders had no role in study design, data collection and analysis, decision to publish, or preparation of the manuscript.".

If you did not receive any funding for this study, please simply state: u201cThe authors received no specific funding for this work.u201d

**Reviewers' comments:**

Reviewer's Responses to Questions

**Comments to the Authors:**

**Please note here if the review is uploaded as an attachment.**

Reviewer #1: The study entitled “Modeling decision dynamics disentangles working memory, cognitive control and reinforcement learning and reveals clinical differences” by Bera and colleagues combines computational models of reinforcement learning, evidence accumulation, and working memory to examine decision-making in healthy controls and clinical groups. The study uses an established reinforcement learning working memory (RLWM) task and extends previous joint modeling of these two components by adding a linear ballistic accumulator to integrate response times into the model. Model estimation relies on advanced simulation-based inference. Next to replicating previous results on the interplay of learning and working memory, the study identifies a novel proactive control process with more cautious responding under higher working memory load. This mechanism, in addition to lower learning rates, is suggested to be altered in patients with schizophrenia compared to healthy controls.

Strengths

(1) Advanced computational model that combines elements of reinforcement learning, working memory, and evidence accumulation

(2) Advanced simulation-based inference approach and convincing validation analyses including parameter recovery and posterior-predictive checks

(3) The study includes patients and control subjects and provides evidence for computational differences in schizophrenia patients

Weaknesses

(1) Fixed beta parameter of softmax function

The beta parameter of the softmax was fixed at 100. This is only very briefly justified, and raises the question of whether this artificially lowers the goodness-of-fit of the choice-only model compared to models with LBA. Was the parameter also fixed for the choice-only model, and if so, why?

(2) Relationship between p(a|s) and pol

On page 16, the authors explain the model. I was wondering whether eq. 4 showing p(a|s) is basically used for computing the “pol_RL” and “pol_WM” variables?

(3) Parameter symbols versus names

Especially in the main manuscript (e.g., Figure 2), the authors do not clearly link the parameter symbols and the function of the parameters. Although symbols and functions are both mentioned in Figure 1, I found it difficult to understand Figure 2 before I had read the methods in detail.

(4) Low learning rates

I was surprised that RL learning rates were quite small (e.g., 0.0087, 0.0047). Can the authors please explain this, and whether behavioral flexibility is more strongly driven by the working memory component?

(5) The results in schizophrenia patients are convincing. However, I was wondering what role their usually reduced cognitive functioning could play here? Might the results be driven by lower cognitive ability in patients? I think it would be relevant to add a paragraph on this to the discussion.

(6) The data are only available upon reasonable request. Is there a particular reason why the authors don’t share their pseudonymized data in a repository? If there are no data protection issues that speak against this, I would recommend that they openly share the data.

Reviewer #2: In this study, the authors present a novel computational framework for jointly modeling choice and response time in the Reinforcement Learning + Working Memory (RLWM) paradigm. Using a 3AFC task on a dataset of 255 participants including controls and patients with schizophrenia, the authors report three main findings: joint modeling improves parameter recovery over choice-only models, participants proactively adjust decision thresholds as a function of working memory load, and schizophrenia patients show a reduced reinforcement learning rate that was missed by prior choice-only analyses. The question of jointly modeling choices and RTs is timely and will be of broad interest to the decision-making community. The work has many strong positive features, including a real methodological contribution, a large sample size, and careful recovery procedures. I therefore have only a few concerns about the modeling and its interpretation, which in its current form leave a few questions open.

1) The RLWM paradigm was designed to dissociate RL from WM under WM-load manipulation, but it is not designed to interrogate RL itself.

1a) A few things about the outcome contingency structure are unclear. The Methods state that correct responses are "probabilistically reinforced, yielding either one or two points," but it is not clear whether this means (a) correct responses are always rewarded with a probabilistic magnitude (1 or 2 points), or (b) reward delivery itself is probabilistic and some correct responses get zero. The two have very different implications for what the learning rates are actually learning from. The model collapses outcomes to binary coding (0/1), but this disregards the magnitude information the participants actually saw (see the broader literature on outcome normalization). Could the authors describe the contingency structure clearly, and give a stronger reason for the binary coding than "prior analysis showed no improvement"?

1b) If I understand correctly, the task consists of 10 iterations per stimulus, deterministic stimulus-action mappings, no reversals or volatility, and a test phase that is excluded from modeling. This raises the concern that the data available to constrain learning rates come from only a small part of the task, which would fit with how small the recovered values are. The methodological contribution that joint modeling improves identifiability under RL/WM competition does not need an ideal RL task, and RLWM is the right design for that claim. But the clinical claim about the RL learning rate in schizophrenia depends on the learning rates being well-identified, and the way the task is built makes that hard. Could the authors say more about what RLWM does and does not imply about RL, and explain why they did not use a task with richer RL structure (probabilistic contingencies, reversals, and so on) in which the design would constrain the learning rates across more than just a few early trials?

1c) Excluding the test phase from modeling is justified on the grounds that prior work found no clinical effects there. But the test phase provides the cleanest read-out of long-term RL retention without WM contributions and adding it to the joint modeling could provide an independent validation of the learning rates estimates.

2) Figure 1C is currently hard to read. It would benefit from more explicit labeling and a consistent color code linking each parameter to the visual element it governs. More generally, the manuscript in its current form will be difficult for any reader who is not already fluent in all of: the RLWM paradigm, the LBA-Angle variant, LANs and simulation-based inference, hierarchical ADVI, Savage–Dickey ratios (which I myself was not familiar with), and the clinical literature on SCZ-RL. A short glossary box or plain-language opening paragraphs would make the paper much easier to get into, without making it any less rigorous.

3) Several methodological choices carry a lot of weight but are introduced in one or two sentences. The ablation experiments in Figure 3 are the main evidence for the paper's claims, but the reader has to work out from the figures why each ablation is the right comparison to run.

4) While the authors acknowledge that joint choice-RT modeling has been attempted before and explain why their model improves on it, some relevant literature, both in learning (e.g., Miletić et al., eLife 2021) and non-learning (e.g., Clithero, J. Econ. Behav. Organ. 2018) contexts, complements the authors' claims and should be cited.

**********

**Have the authors made all data and (if applicable) computational code underlying the findings in their manuscript fully available?**

The PLOS Data policy requires authors to make all data and code underlying the findings described in their manuscript fully available without restriction, with rare exception (please refer to the Data Availability Statement in the manuscript PDF file). The data and code should be provided as part of the manuscript or its supporting information, or deposited to a public repository. For example, in addition to summary statistics, the data points behind means, medians and variance measures should be available. If there are restrictions on publicly sharing data or code —e.g. participant privacy or use of data from a third party—those must be specified.

Reviewer #1: **No:**  Data are not publicly available. This might be related to data protection for clinical subjects. However, this remains elusive.

Reviewer #2: **No:**  Data available upon request; computational code linked but not accessible

**********

PLOS authors have the option to publish the peer review history of their article (what does this mean?). If published, this will include your full peer review and any attached files.

If you choose “no”, your identity will remain anonymous but your review may still be made public.

**Do you want your identity to be public for this peer review?**  For information about this choice, including consent withdrawal, please see our Privacy Policy.

Reviewer #1: No

Reviewer #2: No

[NOTE: If reviewer comments were submitted as an attachment file, they will be attached to this email and accessible via the submission site. Please log into your account, locate the manuscript record, and check for the action link "View Attachments". If this link does not appear, there are no attachment files.]

**Figure resubmission:**

While revising your submission, we strongly recommend that you use PLOS’s NAAS tool (https://ngplosjournals.pagemajik.ai/artanalysis) to test your figure files. NAAS can convert your figure files to the TIFF file type and meet basic requirements (such as print size, resolution), or provide you with a report on issues that do not meet our requirements and that NAAS cannot fix.

After uploading your figures to PLOS’s NAAS tool - https://ngplosjournals.pagemajik.ai/artanalysis, NAAS will process the files provided and display the results in the "Uploaded Files" section of the page as the processing is complete. If the uploaded figures meet our requirements (or NAAS is able to fix the files to meet our requirements), the figure will be marked as "fixed" above. If NAAS is unable to fix the files, a red "failed" label will appear above. When NAAS has confirmed that the figure files meet our requirements, please download the file via the download option, and include these NAAS processed figure files when submitting your revised manuscript.

**Reproducibility:**

?>

# Author comment: Author response to Decision Letter 1

**DOI:** 10.1371/journal.pcbi.1014796.r002

22 Jul 2026

Attachment Submitted filename: Response to Reviewers.docx

# Peer review: Decision Letter 1

**DOI:** 10.1371/journal.pcbi.1014796.r003

**Authors:** Jean Daunizeau, Daniele Marinazzo

1 Sep 2026

Dear Mr Bera,

We are pleased to inform you that your manuscript 'Modeling decision dynamics disentangles working memory, cognitive control and reinforcement learning and reveals clinical differences' has been provisionally accepted for publication in PLOS Computational Biology.

Before your manuscript can be formally accepted you will need to complete some formatting changes, which you will receive in a follow up email. A member of our team will be in touch with a set of requests.

Please note that your manuscript will not be scheduled for publication until you have made the required changes, so a swift response is appreciated.

IMPORTANT: The editorial review process is now complete. PLOS will only permit corrections to spelling, formatting or significant scientific errors from this point onwards. Requests for major changes, or any which affect the scientific understanding of your work, will cause delays to the publication date of your manuscript.

Should you, your institution's press office or the journal office choose to press release your paper, you will automatically be opted out of early publication. We ask that you notify us now if you or your institution is planning to press release the article. All press must be co-ordinated with PLOS.

Thank you again for supporting Open Access publishing; we are looking forward to publishing your work in PLOS Computational Biology.

Best regards,

Jean Daunizeau

Academic Editor

PLOS Computational Biology

Daniele Marinazzo

Section Editor

PLOS Computational Biology

***********************************************************

Reviewer's Responses to Questions

**Comments to the Authors:**

**Please note here if the review is uploaded as an attachment.**

Reviewer #1: Thank you for carefully addressing all of my previous comments. Congratulations on this nice work!

Reviewer #2: The authors have presented a thorough reply to my comments and have addressed my concerns on the original version of their manuscript. I am satisfied with the authors' revision and congratulate them on their interesting work.

**********

**Have the authors made all data and (if applicable) computational code underlying the findings in their manuscript fully available?**

The PLOS Data policy requires authors to make all data and code underlying the findings described in their manuscript fully available without restriction, with rare exception (please refer to the Data Availability Statement in the manuscript PDF file). The data and code should be provided as part of the manuscript or its supporting information, or deposited to a public repository. For example, in addition to summary statistics, the data points behind means, medians and variance measures should be available. If there are restrictions on publicly sharing data or code —e.g. participant privacy or use of data from a third party—those must be specified.

Reviewer #1: Yes

Reviewer #2: None

**********

PLOS authors have the option to publish the peer review history of their article (what does this mean?). If published, this will include your full peer review and any attached files.

If you choose “no”, your identity will remain anonymous but your review may still be made public.

**Do you want your identity to be public for this peer review?**  For information about this choice, including consent withdrawal, please see our Privacy Policy.

Reviewer #1: No

Reviewer #2: No

# Editor report: Acceptance letter

**DOI:** 10.1371/journal.pcbi.1014796.r004

**Authors:** Jean Daunizeau, Daniele Marinazzo

PCOMPBIOL-D-26-00491R1

Modeling decision dynamics disentangles working memory, cognitive control and reinforcement learning and reveals clinical differences

Dear Dr Bera,

I am pleased to inform you that your manuscript has been formally accepted for publication in PLOS Computational Biology. Your manuscript is now with our production department and you will be notified of the publication date in due course.

The corresponding author will soon be receiving a typeset proof for review, to ensure errors have not been introduced during production. Please review the PDF proof of your manuscript carefully, as this is the last chance to correct any errors. Please note that major changes, or those which affect the scientific understanding of the work, will likely cause delays to the publication date of your manuscript.

Soon after your final files are uploaded, unless you have opted out, the early version of your manuscript will be published online. The date of the early version will be your article's publication date. The final article will be published to the same URL, and all versions of the paper will be accessible to readers.

For Research, Software, and Methods articles, you will receive an invoice from PLOS for your publication fee after your manuscript has reached the completed accept phase. If you receive an email requesting payment before acceptance or for any other service, this may be a phishing scheme. Learn how to identify phishing emails and protect your accounts at https://explore.plos.org/phishing.

Thank you again for supporting PLOS Computational Biology and open-access publishing. We are looking forward to publishing your work!

With kind regards,

Sharmila Kamatchi

PLOS Computational Biology | Carlyle House, Carlyle Road, Cambridge CB4 3DN | United Kingdom ploscompbiol@plos.org | Phone +44 (0) 1223-442824 | ploscompbiol.org | @PLOSCompBiol
